# A novel approach for predicting protein S-glutathionylation

**DOI:** 10.1186/s12859-020-03571-w

**Published:** 2020-09-14

**Authors:** Anastasia A. Anashkina, Yuri M. Poluektov, Vladimir A. Dmitriev, Eugene N. Kuznetsov, Vladimir A. Mitkevich, Alexander A. Makarov, Irina Yu. Petrushanko

**Affiliations:** 1 Engelhardt Institute of Molecular Biology, Russian Academy of Sciences, Vavilov St. 32, 119991 Moscow, Russia; 2 V. A. Trapeznikov Institute of Control Sciences of Russian Academy of Sciences, 65 Profsoyuznaya street, Moscow, 117997 Russia

**Keywords:** S-glutathionylation, Peptide sequence, Protein glutathionylation, Redox modification, Prediction method, Glutathionylation propensity score

## Abstract

**Background:**

S-glutathionylation is the formation of disulfide bonds between the tripeptide glutathione and cysteine residues of the protein, protecting them from irreversible oxidation and in some cases causing change in their functions. Regulatory glutathionylation of proteins is a controllable and reversible process associated with cell response to the changing redox status. Prediction of cysteine residues that undergo glutathionylation allows us to find new target proteins, which function can be altered in pathologies associated with impaired redox status. We set out to analyze this issue and create new tool for predicting S-glutathionylated cysteine residues.

**Results:**

One hundred forty proteins with experimentally proven S-glutathionylated cysteine residues were found in the literature and the RedoxDB database. These proteins contain 1018 non-S-glutathionylated cysteines and 235 S-glutathionylated ones. Based on 235 S-glutathionylated cysteines, non-redundant positive dataset of 221 heptapeptide sequences of S-glutathionylated cysteines was made. Based on 221 heptapeptide sequences, a position-specific matrix was created by analyzing the protein sequence near the cysteine residue (three amino acid residues before and three after the cysteine). We propose the method for calculating the glutathionylation propensity score, which utilizes the position-specific matrix and a criterion for predicting glutathionylated peptides.

**Conclusion:**

Non-S-glutathionylated sites were enriched by cysteines in − 3 and + 3 positions. The proposed prediction method demonstrates 76.6% of correct predictions of S-glutathionylated cysteines. This method can be used for detecting new glutathionylation sites, especially in proteins with an unknown structure.

## Introduction

S-glutathionylation is the reversible formation of disulfide bonds between cysteine residues of a protein and glutathione tripeptide associated with cell response to changes in redox conditions, including hypoxia, ischemia and reperfusion. This mechanism is an important part of the co- and post-translational regulation of the function of various proteins [1–3]. Glutathionylation of proteins is a protective and adaptive mechanism. It affects a number of transport proteins, such as Serca, RyR, VGCC, which allow maintaining normal calcium flow in the myocardium under various pathological conditions [4]. Undoubtedly, glutathionylation also has a regulatory effect, affecting protein functionality depending on the redox status in the cell [5–7]. For example, glutathionylation of Na,K-ATPase occurs during hypoxia, which leads to Na,K-ATPase partial inhibition and helps to save ATP for cell survival [3, 8].

Thus, the determination of cysteine residues, which are potential targets for glutathionylation, is important for understanding the mechanisms of glutathionylation effect on proteins functioning and confirmation of targets by mutagenesis. Prediction of glutathionylating cysteine residues allows finding new target proteins that alter function in pathologies associated with impaired redox status.

There are a number of studies based on these collected data on the prediction of glutathionylation sites in proteins [9–13]. An analysis of flanking regions in some studies showed the presence of negatively charged residues around S-glutathionylated cysteines [10], while in others, the preference for positively charged ones [6, 12] was substantiated. In all papers published in this field, the cysteines that were not found to be glutathionylated were selected as negative control [9–14]. Namely, those cysteines that did not react in the specific conditions of the glutathionylation experiment. However, the reason for the non-interaction of such cysteines with glutathione can have a different cause. Firstly, the cysteine can be located inside the protein or in transmembrane region and not be accessible to the solvent. Secondly, reactivity of thiol group concerns with its deprotonation which depends on pKa group and environmental conditions. So it is difficult to create true negative dataset. We suggested that the ability of cysteines to react with glutathione can be affected by amino acids located quite close in the chain. To test our hypothesis, we analyzed short (3 aa) sequences of flanking regions of a non-redundant set of 221 experimentally confirmed glutathionylation sites in 140 proteins and proposed a simple method for predicting glutathionylation.

## Materials and methods

### Existing databases and services on S-glutathionylation prediction

RedoxDB was the first protein glutathionylation database [14]. It included 242 experimentally verified S-glutathionylation sites on 153 S-glutathionylation proteins, which subsequently, together with data from SGDB [9], became the basis for the dbGSH database [10]. The dbGSH collection also includes 19 experimentally verified S-glutathionylation sites (GSH) in UniProt release 2013–03, 75 experimentally verified S-glutathionylation sites on 37 S-glutathionylation proteins from paper [10] and 1816 experimentally verified S-glutathionylation sites in 1011 S-glutathionylation proteins from paper [12]. A server PGluS for predicting glutathionylation sites was presented in [11]. Nowadays there is only one GSHSite server [12], which stores data on known glutathionylation sites and allows predicting glutathionylation sites in protein (http://csb.cse.yzu.edu.tw/GSHSite/index.php).

### S-glutathionylated cysteines positive non-redundant dataset

S-glutathionylathion sites were obtained from the reviews dedicated to regulatory glutathionylation of proteins [2, 15, 16] and from databases RedoxDB [14] and hprd.org. Sequences of animal and human proteins, where glutathionylation was confirmed by methods of molecular biology (mass spectrometry and site-directed mutagenesis) were selected for further analysis. Some sequences were obtained from uniprot.org, search was performed using the keyword “Glutathionylation [KW-0318]”. Sequences were taken as heptapeptides, containing three aminoacid residues to the left and to the right from the target cysteine residue.

Repeating heptapeptides were excluded from S-glutathionylation positive dataset. Thus, 221 non-redundant S-glutathionylation sites of 140 proteins with experimental verification were found (see Additional file 1).

### Negative dataset

For a negative dataset, we needed to find experimentally proven cases where the cysteine residue of protein, being available to the solvent and glutathione, does not react with glutathione. We have found cases where only a part of cysteines is glutathionylated in a protein. However, it turned out to be impossible to prove that non-S-glutathionylated cysteines are freely available to the solvent and glutathione. Therefore non-S-glutathionylated cysteines were taken for the negative dataset, as in the other studies [9–14]. We excluded from the negative sample 33 cysteines located, according to Uniprot, in transmembrane domains, since such cysteines are not accessible to the solvent. So, negative dataset contains 1047 non-S-glutathionylated cysteine residues from 140 proteins.

### Position specific matrix (PSM)

Non-redundant positive dataset of 221 heptapeptides became the basis for position specific matix (Table 1). Elements of the table represent the occurrence of amino acid residues at a given position. For example, in the third position to the left from cysteine, alanine was found 20 times and in the first position to the right from cysteine, glycine was found 24 times in a set of 221 heptapeptides.

**Table 1:** The occurrence of amino acid residues in short flanking regions around glutathionylation sites calculated from the 221 heptapeptide sequences (position-specific matrix, PSM)

AminoAcid	Positions in heptapeptide sequence						
	L3	L2	L1	C	R1	R2	R3
–	2	0	0	0	2	4	4
A	20	14	28	0	14	19	18
C	2	4	5	221	4	4	3
D	11	9	14	0	13	12	12
E	16	15	10	0	13	16	20
F	9	9	7	0	11	7	5
G	16	17	20	0	24	14	15
H	5	7	7	0	5	8	5
I	12	10	15	0	8	4	6
K	14	18	14	0	14	13	18
L	15	17	26	0	18	22	21
M	3	5	3	0	8	4	8
N	8	7	7	0	5	6	13
P	10	18	8	0	16	15	12
Q	9	3	5	0	2	11	8
R	13	8	9	0	9	12	11
S	15	10	13	0	19	14	18
T	11	25	11	0	13	17	9
V	14	14	13	0	14	12	8
W	4	2	1	0	1	1	2
Y	12	9	5	0	8	6	5

### Glutathionylation propensity score S

**Fig. 1:**
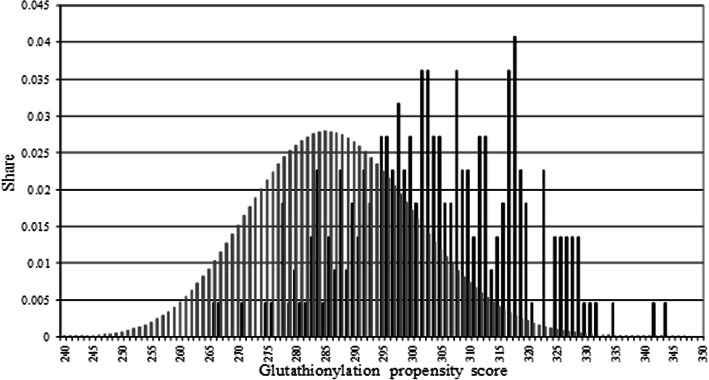
Relative frequencies of score S for all combinatorially possible heptapeptide sequences (gray) and for heptapeptides with experimentally shown glutathionylation of cysteine (black) in the central position

We proposed a glutathionylation propensity score S, calculated by the position-weight matrix as the sum of the elements equal to the occurrence of these residues at the corresponding positions. For example, for the WRVCALL heptapeptide, the S value will be 4 + 8 + 13 + 221 + 14 + 22 + 21 = 303 (Table 1). Such values are given for each heptapeptide from our datasets (Fig. 1; Additional file 1).

### Glutathionylation propensity score distribution for all combinatorially possible heptapeptide sequences with cysteine in the central position

The distribution of the score for all possible sequences of heptapeptides with cysteine in the central position is calculated by enumerating 20 aminoacids in three positions to the right and three positions to the left from the cysteine residue (Fig. 1). Thus, this gives 20^6^ possible variants of such sequences (since is only the cysteine in the central position of all such sequences).

### Glutathionylation propensity score cut-off value

Prediction with respect to glutathionylation was done on the basis of S score. Cut off value splits graphs of the distributions for all combinatorially possible heptapeptide sequences and for positive dataset sequences so that areas under the curves are equal (Fig. 1). Сut off value was found at S = 294. Sequences having S > 294 were predicted to undergo glutathionylation. Sequences with S ≤ 294 were predicted not to undergo glutathionylation. Prediction was carried out for positive dataset as well as negative dataset (see Additional file 1). The predicted glutathionylation status was compared with the actual glutathionylation status by calculating sensitivity, specificity, accuracy, balanced accuracy and Mathew’s correlation coefficient (as in [12], for example).

### Validation using three control elements procedure (jackknife resampling)

To perform a validity test we removed three randomly selected heptapeptides from non-redundant positive dataset, then we constructed PSM for the remaining 218 heptapeptides from the dataset (by subtracting from the original matrix the occurrence of residues of the selected peptide). Next, using a modified position-specific matrix, we calculated Glutathionylation Propensity Scores for all possible heptapeptide sequences with cysteine in the central position and for new positive dataset of 218 heptapeptides. For excluded peptides, we calculated the Glutathionylation Propensity Score and compared it with a threshold value. In each experiment, it was determined how many of the three heptapeptides were recognized correctly (3, 2, 1, 0). Jackknife resampling was repeated 200 times. The overall accuracy of the method was determined as the number of correctly defined sequences of 3 * 200 = 600 control points.

## Results

### The frequency of occurrence of amino acids in short flanking regions around cysteines susceptible to glutathionylation

**Fig. 2:**
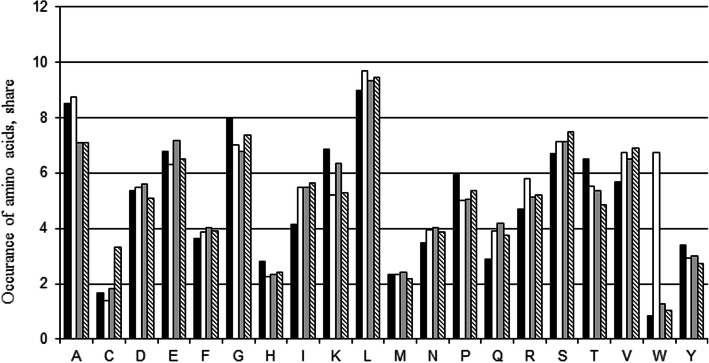
The occurrence of amino acid residues in the flanking sites of glutathionylation sites. Occurrence in the positive dataset (black), in 5029 proteomes (white), in this dataset of 140 proteins (gray) and in the negative dataset (striped)

We selected 140 proteins containing cysteines susceptible to glutathionylation to analyze the effect of short flanking regions (Additional file 1). All cysteines susceptible to glutathionylation were extracted as heptapeptides with cysteine in the center. Duplicated heptapeptide sequences were excluded. A non-redundant set of 221 heptapeptide sequences with S-glutathionylated cysteine in the center was used for analysis. A matrix reflecting the occurrence of various types of amino acid residues in three positions to the right and left of such cysteine (Table 1, Fig. 2) was compiled.

Figure 2 shows that all types of amino acids are found in flanking regions. The occurrence of amino acid residues in flanking regions is slightly different from the occurrence of amino acid residues in 5029 proteomes. The exception is the significantly lower occurrence of tryptophan around S-glutathionylation sites than in 5029 proteomes. But one can see that tryptophan is less common in our protein set than in 5029 proteomes. Slightly more often than in 5029 proteomes, histidine, glycine, lysine, proline, and threonine are found around the S-glutathionylated cysteine residues. A little less often - isoleucine, asparagine, glutamine, arginine, serine, valine.

**Fig. 3:**
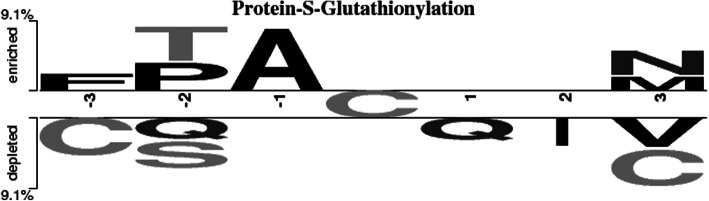
TwoSampleLogo presents the compositional biases of amino acids around S-glutathionylation sites compared to non-S-glutathionylation sites in the same 140 proteins. The significant amino acids around S-glutathionylated cysteine residue is enriched in the positive dataset and presented in upper panel (*p* < 0.05). Relatively, the high frequency around non-S-glutathionylated cysteines is depleted in the negative dataset and presented in lower panel

Comparison of the occurrence of amino acid residues in the positive and negative heptapeptide datasets by the TwoSampleLogo method showed that cysteines are often found in non-S-glutathionylated heptapeptides at positions + 3 and − 3 from the central cysteine residue (Fig. 3). We suggest that in such pairs of cysteines a disulfide bond forms that protects cysteine residues from oxidation under conditions of oxidative stress. Such a mechanism is implemented in the ribonuclease inhibitor protein [17].

### Protein-S-Glutathionylation prediction method by amino acid context

To construct a simple method for predicting glutathionylation, we proposed to assign a glutathionylation propensity score S, calculated by a position-specific matrix as the sum of the elements equal to the occurrence of these residues at the corresponding positions, to each heptapeptide. So, for example, for the heptapeptide WRVCALL, this S value will be 4 + 8 + 13 + 221 + 14 + 22 + 21 = 303 (Table 1).

We calculated the S value for each of the 221 heptapeptides analyzed with the cysteine residue in central position and examined the distribution of its values (Fig. 1). One can see that the distribution is normal-like shape and unimodal.

Strictly speaking, in order to construct a prediction method, it is necessary to have data of two equal samples: positive and negative.

However, we believe that it is possible to find experimental conditions that any cysteine available to the solvent will interact with glutathione. Thus, the set of non-S-glutathionylated cysteines is fundamentally absent. Indeed, it was not possible to find experimentally confirmed cases in the literature that cysteines, being accessible to the solvent and glutathione, do not react with it. In the absence of confirmed data on non-S-glutathionylated cysteines, we used the distribution of S score for all combinatorially possible heptapeptides with cysteine in the central position as the base sample (the total number of such heptapeptides is 20^6^).

We compared the distribution of S score for the 221 S-glutathionylated cysteine heptapeptides with the S score distribution for all combinatorially possible heptapeptide sequences with cysteine in the central position. Figure 1 shows the relative frequencies of score S values for all possible heptapeptides and for 221 heptapeptides with S-glutathionylated cysteine.

For the formal separation of these two distributions, we used the criteria of Mann-Whitney [18] and Wilcoxon [19] nonparametric statistics. These criteria do not require the distribution law of the compared values and can be used in situations where two unimodal distributions differ in mathematical expectations.

The calculated Mann-Whitney statistics U = 1050 (the number of degrees of freedom df = 51). The probability that these two distributions differ is *P* = 0.953 (i.e., they differ with a significance level of 0.047). The Wilcoxon test also gives a similar result (a sample value of Wilcoxon statistics W = 1.692, the number of degrees of freedom df = 51, the probability of differences in distributions *P* = 0.955, *p* = 0.045). Thus, both tests confirm the formal difference between these distributions.

In order to make the simplest decisive rule for predicting S-glutathionylation of central cysteine of heptapeptide, it is necessary to determine the cut-off value with respect to the areas under the graphs of the two compared distributions that are equal. Having determined the area under the distribution curves, we found that this boundary is S = 294. Thus, the decision on whether the heptapeptide belongs to the class of S-glutathionylated is made if the value of the calculated glutathionylation propensity score S exceeds the threshold value of 294.

Conclusions on this decision rule, as when testing any statistical hypothesis, are formulated with varying degrees of confidence. Namely, we make the prediction of cysteine S-glutathionylation with greater confidence than non-S-glutathionylation. This is due to the lack of experimentally proven negative control, i.e. such cysteines on the surface of the protein, which, being accessible to the solvent and glutathione, do not react with it.

We applied the proposed decision rule to 1282 cysteines from 140 S-glutathionylated proteins (see Additional file 2, lines 1284–1293). It can be seen that in 180 cases a truly positive forecast was obtained, 628 false positives, 55 false negative and 419 true negative forecasts (Table 2). We compared our results with the prediction computed by the method proposed by Pal et.al [13]. It can be seen that the scores of Sensitivity and Matthew’s correlation coefficient(MCC) in our method turned out better. Specificity and Accuracy are approximately equal. Note that for our purposes, it is important not to miss the S-glutathionylated cysteine residues, which means maximizing the Sensitivity score.

**Table 2:** PSM prediction results and comparison with Pal et al. [14] in a sample of 1282 cysteines from 140 proteins

	PSM	Pal et al. [14]
TruePositive	180	161
FalsePositive	628	612
FalseNegative	55	74
TrueNegative	419	435
Sensitivity	0.766	0.685
Specificity	0.400	0.415
Accuracy	0.467	0.465
Balanced accuracy	0.583	0.550
MCC	0.133	0.080

To verify the reliability of the proposed method for the recognition of S-glutathionylated cysteines, we used modern resampling methods. The main advantage of this popular approach is the absence of the need to make and verify any assumptions about the distribution law of the variables. We made 200 Jackknife resampling experiments as described in the Materials and Methods section. Jackknife resampling with a random exclusion of three elements showed that in 6 cases out of 200, no control heptapeptide (out of the three excluded when calculating the PSM matrix) was correctly recognized by the proposed method. In 25 cases out of 200, one control peptide of the three was correctly recognized, in 102 cases two control peptides were recognized, and in 68 cases all three control peptides were recognized correctly. This indicates a good predictive ability of the proposed method on real data. The percentage of correct classification is 72.2% (Sensitivity).

## Discussion

Glutathionylation plays an indispensable role in protecting cell viability during oxidative stress. S-glutathionylation prevents irreversible oxidation of cysteines and protects proteins from subsequent inactivation and degradation. Some glutathionylated proteins significantly change their function, which helps the cell adapt to altered redox conditions. For example, inhibition of Na,K-АТPase under hypoxia prevents ATP depletion and promotes cell survival [3].

The prediction of S-glutathionylated cysteine residues in proteins is very important. It will allow checking the predicted residues by site-directed mutagenesis, analyzing structural changes and understanding how the function of these proteins changes during oxidative stress. It is necessary to have as full as possible prediction of S-glutathionylated cysteine residues. In this case, it is more important not to lose potential candidates than to receive false positive predictions. This requirement corresponds to maximization of the Sensitivity score, which reflects the completeness of prediction of the positive dataset.

The interaction of glutathione with cysteine residues of proteins is possible due to at least two critical factors. The first is the accessibility of the cysteine residue for the solvent, and, accordingly, for interaction with glutathione. The flanking regions around cysteine indirectly characterize its position in the protein structure. Thus, the presence of hydrophobic residues may suggest the location of this cysteine inside the protein. The presence of charged, polar or residues with hydrogen bonds may indicate the location of such cysteine on the surface and accessibility to the solvent. However, it is impossible to check the accessibility of cysteine to solvent in the absence of the structure of this protein. Even the presence of an experimentally determined protein structure cannot guarantee the absence of steric difficulties in the interaction of glutathione with cysteine in vitro and in vivo. This is evident, because there are local and even global differences in the three-dimensional structure of protein under different conditions.

The second factor is the reactivity of cysteine residue. Reactivity is determined by many factors, such as the properties of the solution (in particular pH, ionic strength, the presence of ions, the redox status of the cell) and amino acid context of the sequence, which affects the shift in electron density. Adjacent positively charged amino acids can facilitate deprotonation of cysteines SH group (pKa usually 8.3–8.5; ranges from 3.4 to 9.5) and increase their reactivity [6]. It is possible to select appropriate experimental conditions for the reaction of any accessible protein cysteine with glutathione. A pure experiment to evaluate the influence of amino acid context and environmental factors is the experiment with short peptides containing cysteine. However, no such experiments have been carried out so far. Thus it is necessary to predict cysteine reactivity for glutathionylation in physiological conditions. Thus, it is impossible to create true negative dataset until we discover influence of aminoacid environment on cysteines reactivity.

We have created S-glutathionylation predictor that does not take into account the negative control (Table 1, Fig. 1). Choosing a cut-off value of 294, which corresponds to the equality of the right and left parts of graph 3, we obtained 76.6% of the correctly predicted positive dataset. By reducing the cut-off value, we can increase the Sensitivity. Other quality indicators naturally get worse. By lowering the cut-off value to 279, we can get the correct prediction of more than 95% of glutathionylated cysteines. Naturally, the number of false positive predictions increases. However, as mentioned above, in this case it is more important not to miss possible glutathionylation sites. False positive sites can be excluded experimentally.

In addition, we suggest that the spatial context may affect the ability to react with glutathione. That is, amino acid residues that are distant in sequence but close in space can, in our opinion, affect the reactivity of this cysteine to glutathionylation. This assumption requires further study.

Possibly, recognition indicators of glutathione-cysteine can be improved if not all theoretically possible sequences of seven residues (heptapeptides) with cysteine in the central position were considered for comparison with S-glutathionylated heptapeptide sequences dataset, but only those that really do not react with glutathione when accessible to solvent and glutathione. In addition, it is evident that not all combinatorially possible heptapeptide sequences exist in nature. It can also be expected that the accuracy of the prediction will increase with an increase in the number of known of S-glutathionylation sites.

In this article, we have presented simple successful predictor for identifying S-glutathionylated sites from short flanking sequences around cysteine residues. The proposed predictor is based on position-specific matrix, S-glutathionylation propensity score calculation and cut-off value of 294. The performance of the method was measured with a sensitivity of 76.6% and MCC of 0.13. Additionally, the proposed method was evaluated using a Jackknife resampling experiment resulting in a sensitivity of 72.2%. The proposed method may be useful for detecting new glutathionylation sites, especially in proteins with an unknown structure. Sensitivity achieved with our position-specific matrix is greater than when calculations were performed using the method of Pal et al. [13]. In this paper authors implemented a very similar approach. Pentapeptides were used (sequences with two amino acid residues around glutathionylation site) to calculate position dependent F-scores, which measure how a particular amino acid at a particular position may affect the likelihood of glutathionylation event. Glutathionylation-score, indicating propensity of a sequence to undergo glutathionylation, was calculated using position-dependent F-scores for each amino-acid. We demonstrated that taking into account not two, but three amino acids around the glutathionylation site improves prediction (Table 2). Another existing prediction tool, GSHSite server [12], is based on the analysis of long flanking areas (ten residues on each side). An orthogonal binary coding scheme was adopted to transform amino acids into numeric vectors, in the so-called 20-dimensional binary coding. For the composition of 20 amino acids surrounding the S-glutathionylation sites, the vector had 20 elements for the amino acid composition and 441 elements for the amino acid pair composition. High dimensional vectors are analyzed by support vector machine and maximal dependence decomposition methods. GSHSite server functions well on those proteins that are included in its own database. However, it does not function for proteins not included in this database. In addition, if the target cysteine residue is located at a distance of less than 10 amino acids from the N- or C-terminus of the protein, then analysis is not performed.

## Conclusions

Here the new method for prediction of glutathionylation sites in proteins and peptides has been developed. High sensitivity of the method allows not to lose potential glutathionylation sites in proteins. The matrix reflecting the occurrence of various types of amino acid residues in three positions to the right and left of such cysteine was created. Non-S-glutathionylated sites were enriched by cysteines in − 3 and + 3 positions. The proposed method is useful for detecting new glutathionylation sites, especially in proteins with an unknown structure.

## Supplementary information

**Additional file 1.**

**Additional file 2.**

## Data Availability

Part of the data generated or analyzed during this study are included in this published article and its supplementary information files. Other data are available from the corresponding author on reasonable request.

## Abbreviations

PSM: Position specific matrix
GPS: Glutathionylation propensity score
