# Supplementary material for: A novel approach for predicting protein S-glutathionylation
Source: BMC Bioinformatics. 2020 Sep 14;21(Suppl 11):282. doi: 10.1186/s12859-020-03571-w (PMC7489215; doi:10.1186/s12859-020-03571-w)
Supplement: Supplementary file 1 — Additional file 1. [file 12859_2020_3571_MOESM1_ESM.doc]

**Calculation of sensitivity, specificity, accuracy, balanced accuracy and Mathew’s correlation coefficient**

The predicted glutathionylation status was compared with the actual glutathionylation status by calculating the following parameters:

$\text{Sensitivity=}\frac{\text{TP}}{\text{TP}+\text{FN}}$ (1)

$\text{Specificity=}\frac{\text{TN}}{\text{TN}+\text{FP}}$ (2)

$\text{Accuracy=}\frac{\text{TP+TN}}{\text{TP+TN+FP+FN}}$ (3)

$BalancedAccuracy=\frac{Sensitivity+Specificity}{2}$ (4)

Mathew’s correlation coefficient

$\text{MCC=}\frac{\text{TP*TN-FP*FN}}{\sqrt{\text{(TP+FP)*(TP+FN)*(FP+TN)*(TN+FN)}}}$ (5)

where TP is true positive, TN is true negative, FP is false positive, FN is false negative, MCC is Mathew’s correlation coefficient.

Table 1. A list of 140 proteins with S-glutathionylated cysteines.

| **N** | **Protein** | **Organizm** | **Uniprot ID** | **Reference** | **All Cys** | **Cys-SG** |  |  | **Cys-SG** | **S score** | **PSM** | **Pal score** | **Pal** |
| --- | --- | --- | --- | --- | --- | --- | --- | --- | --- | --- | --- | --- | --- |
| **1** | Aldose reductase | Homo sapiens | P15121 | [1] | 7 | 1 | 45 | HIDCAHV | 0 | 280 | 0 | 1.1248 | 1 |
|  |  |  |  |  |  |  | 81 | KLWCTYH | 0 | 277 | 0 | 0.8958 | 0 |
|  |  |  |  |  |  |  | 93 | KGACQKT | 0 | 304 | 1 | 0.9850 | 0 |
|  |  |  |  |  |  |  | 187 | QIECHPY | 0 | 275 | 0 | 1.0498 | 1 |
|  |  |  |  |  |  |  | 200 | IQYCQSK | 0 | 275 | 0 | 0.9008 | 0 |
|  |  |  |  |  |  |  | **299** | **WRVCALL** | **1** | **303** | 1 | 0.9263 | 0 |
|  |  |  |  |  |  |  | 304 | LLSCTSH | 0 | 298 | 1 | 1.0015 | 1 |
| **2** | ATP-binding cassette sub-family C member 8 | Homo sapiens | Q09428 | [1] | 28 | 1 | 6 | LAFCGSE | 0 | 315 | 1 | 1.0613 | 1 |
|  |  |  |  |  |  |  | 26 | NNGCFVD | 0 | 291 | 0 | 1.1070 | 1 |
|  |  |  |  |  |  |  | 87 | VLVCEIA | 0 | 300 | 1 | 1.0740 | 1 |
|  |  |  |  |  |  |  | 170 | LRFCLTG | 0 | 301 | 1 | 0.8763 | 0 |
|  |  |  |  |  |  |  | 267 | QRLCEAF | 0 | 301 | 1 | 0.9750 | 0 |
|  |  |  |  |  |  |  | 319 | GPLCIFG | 0 | 311 | 1 | 1.0153 | 1 |
|  |  |  |  |  |  |  | 418 | GQICNLV | 0 | 290 | 0 | 1.0245 | 1 |
|  |  |  |  |  |  |  | 435 | FFLCPNL | 0 | 308 | 1 | 1.0193 | 1 |
|  |  |  |  |  |  |  | 624 | EEQCAPH | 0 | 291 | 0 | 1.1118 | 1 |
|  |  |  |  |  |  |  | 656 | REDCRGL | 0 | 307 | 1 | 1.0865 | 1 |
|  |  |  |  |  |  |  | 677 | ADNCCVQ | 0 | 281 | 0 | 1.0235 | 1 |
|  |  |  |  |  |  |  | 678 | DNCCVQI | 0 | 275 | 0 | 0.9203 | 0 |
|  |  |  |  |  |  |  | **717** | **QVGCGKS** | **1** | 319 | 1 | 1.1178 | 1 |
|  |  |  |  |  |  |  | 805 | IEACSLQ | 0 | 325 | 1 | 1.0200 | 1 |
|  |  |  |  |  |  |  | 921 | RSECQLF | 0 | 283 | 0 | 0.9593 | 0 |
|  |  |  |  |  |  |  | 1000 | WRACAKY | 0 | 293 | 0 | 0.9975 | 0 |
|  |  |  |  |  |  |  | 1050 | ARNCSLS | 0 | 315 | 1 | 0.8883 | 0 |
|  |  |  |  |  |  |  | 1056 | SQECTLD | 0 | 296 | 1 | 1.0615 | 1 |
|  |  |  |  |  |  |  | 1071 | TVLCSLG | 0 | 328 | 1 | 0.9065 | 0 |
|  |  |  |  |  |  |  | 1078 | IVLCLVT | 0 | 312 | 1 | 0.9798 | 0 |
|  |  |  |  |  |  |  | 1128 | SSDCNTI | 0 | 288 | 0 | 1.1545 | 1 |
|  |  |  |  |  |  |  | 1141 | TLECLSR | 0 | 302 | 1 | 0.9743 | 0 |
|  |  |  |  |  |  |  | 1148 | TLLCVSA | 0 | 321 | 1 | 0.9738 | 0 |
|  |  |  |  |  |  |  | 1174 | AIVCYFI | 0 | 285 | 0 | 0.9923 | 0 |
|  |  |  |  |  |  |  | 1257 | IGACVVL | 0 | 325 | 1 | 1.1540 | 1 |
|  |  |  |  |  |  |  | 1377 | IGICGRT | 0 | 310 | 1 | 1.1055 | 1 |
|  |  |  |  |  |  |  | 1445 | ERKCSDS | 0 | 308 | 1 | 0.8118 | 0 |
|  |  |  |  |  |  |  | 1490 | QLFCLAR | 0 | 302 | 1 | 0.9100 | 0 |
| **3** | Ryanodine receptors (RyR1) | Rattus norvegicus | F1LMY4 | [1] | 99 | 1 | 25 | VLQCSAT | 0 | 304 | 1 | 0.9340 | 0 |
|  |  |  |  |  |  |  | 37 | LKLCLAA | 0 | 335 | 1 | 0.9703 | 0 |
|  |  |  |  |  |  |  | 48 | NRLCFLE | 0 | 316 | 1 | 0.8633 | 0 |
|  |  |  |  |  |  |  | 66 | LAICCFI | 0 | 282 | 0 | 0.9538 | 0 |
|  |  |  |  |  |  |  | 67 | AICCFIL | 0 | 292 | 0 | 0.8978 | 0 |
|  |  |  |  |  |  |  | 121 | YLSCLTT | 0 | 307 | 1 | 0.9813 | 0 |
|  |  |  |  |  |  |  | 147 | GEACWWT | 0 | 291 | 0 | 1.0075 | 1 |
|  |  |  |  |  |  |  | 207 | NPICSCC | 0 | 288 | 0 | 0.8105 | 0 |
|  |  |  |  |  |  |  | 209 | ICSCCEE | 0 | 290 | 0 | 0.7223 | 0 |
|  |  |  |  |  |  |  | 210 | CSCCEEG | 0 | 282 | 0 | 0.9600 | 0 |
|  |  |  |  |  |  |  | 231 | MDECLTI | 0 | 284 | 0 | 1.1530 | 1 |
|  |  |  |  |  |  |  | 254 | GPVCTHA | 0 | 307 | 1 | 1.0493 | 1 |
|  |  |  |  |  |  |  | 316 | TSFCFRI | 0 | 278 | 0 | 0.9520 | 0 |
|  |  |  |  |  |  |  | 347 | ESLCFVQ | 0 | 304 | 1 | 0.9843 | 0 |
|  |  |  |  |  |  |  | 394 | LTRCQQE | 0 | 303 | 1 | 0.8540 | 0 |
|  |  |  |  |  |  |  | 491 | VLNCIDR | 0 | 290 | 0 | 1.0210 | 1 |
|  |  |  |  |  |  |  | 538 | RTNCALF | 0 | 307 | 1 | 1.0763 | 1 |
|  |  |  |  |  |  |  | 567 | VLYCVLI | 0 | 299 | 1 | 1.0113 | 1 |
|  |  |  |  |  |  |  | 605 | DVLCSLC | 0 | 316 | 1 | 0.9065 | 0 |
|  |  |  |  |  |  |  | 608 | CSLCVCN | 0 | 290 | 0 | 0.8073 | 0 |
|  |  |  |  |  |  |  | 610 | LCVCNGV | 0 | 280 | 0 | 0.8555 | 0 |
|  |  |  |  |  |  |  | 747 | VVSCCLD | 0 | 300 | 1 | 0.8483 | 0 |
|  |  |  |  |  |  |  | 748 | VSCCLDL | 0 | 301 | 1 | 0.7973 | 0 |
|  |  |  |  |  |  |  | 763 | INGCPVQ | 0 | 296 | 1 | 1.2295 | 1 |
|  |  |  |  |  |  |  | 812 | YAPCHEA | 0 | 294 | 0 | 1.0703 | 1 |
|  |  |  |  |  |  |  | 846 | PSRCLSH | 0 | 287 | 0 | 0.8193 | 0 |
|  |  |  |  |  |  |  | 855 | FVPCPVD | 0 | 292 | 0 | 1.1968 | 1 |
|  |  |  |  |  |  |  | 907 | LHPCLVD | 0 | 293 | 0 | 1.0175 | 1 |
|  |  |  |  |  |  |  | 938 | ALGCHVG | 0 | 310 | 1 | 0.9945 | 0 |
|  |  |  |  |  |  |  | 1041 | DSLCQAV | 0 | 297 | 1 | 0.9068 | 0 |
|  |  |  |  |  |  |  | 1151 | VVGCMID | 0 | 293 | 0 | 1.0898 | 1 |
|  |  |  |  |  |  |  | 1192 | LPVCSLG | 0 | 323 | 1 | 0.9563 | 0 |
|  |  |  |  |  |  |  | 1217 | FAICGLQ | 0 | 313 | 1 | 1.1398 | 1 |
|  |  |  |  |  |  |  | 1269 | TPPCLRL | 0 | 309 | 1 | 1.0485 | 1 |
|  |  |  |  |  |  |  | 1303 | HFRCTAG | 0 | 291 | 0 | 0.9318 | 0 |
|  |  |  |  |  |  |  | 1447 | EPSCVWV | 0 | 291 | 0 | 1.0240 | 1 |
|  |  |  |  |  |  |  | 1489 | SLKCSNC | 0 | 295 | 1 | 0.8245 | 0 |
|  |  |  |  |  |  |  | 1492 | CSNCYMV | 0 | 260 | 0 | 0.9633 | 0 |
|  |  |  |  |  |  |  | 1518 | VIGCLVD | 0 | 307 | 1 | 1.0858 | 1 |
|  |  |  |  |  |  |  | 1591 | APQCPPR | 0 | 306 | 1 | 1.2058 | 1 |
|  |  |  |  |  |  |  | 1630 | AVQCQEP | 0 | 290 | 0 | 0.9935 | 0 |
|  |  |  |  |  |  |  | 1685 | QPGCYHV | 0 | 292 | 0 | 1.0540 | 1 |
|  |  |  |  |  |  |  | 1723 | ESACRSR | 0 | 309 | 1 | 0.9805 | 0 |
|  |  |  |  |  |  |  | 1781 | SPPCFVV | 0 | 293 | 0 | 1.0960 | 1 |
|  |  |  |  |  |  |  | 1939 | LQMCHLL | 0 | 290 | 0 | 0.8593 | 0 |
|  |  |  |  |  |  |  | 1946 | EYFCDQE | 0 | 297 | 1 | 0.9478 | 0 |
|  |  |  |  |  |  |  | 2020 | EEDCPLP | 0 | 316 | 1 | 1.2245 | 1 |
|  |  |  |  |  |  |  | 2041 | LAHCGIQ | 0 | 293 | 0 | 1.0668 | 1 |
|  |  |  |  |  |  |  | 2157 | LLECLGQ | 0 | 303 | 1 | 1.0065 | 1 |
|  |  |  |  |  |  |  | 2231 | VTSCCRF | 0 | 294 | 0 | 0.8978 | 0 |
|  |  |  |  |  |  |  | 2232 | TSCCRFL | 0 | 284 | 0 | 0.7895 | 0 |
|  |  |  |  |  |  |  | 2236 | RFLCYFC | 0 | 287 | 0 | 0.9305 | 0 |
|  |  |  |  |  |  |  | 2239 | CYFCRIS | 0 | 270 | 0 | 0.8940 | 0 |
|  |  |  |  |  |  |  | 2394 | LAGCGLQ | 0 | 324 | 1 | 1.1760 | 1 |
|  |  |  |  |  |  |  | 2309 | LQSCPML | 0 | 293 | 0 | 1.0618 | 1 |
|  |  |  |  |  |  |  | 2325 | WNPCGGE | 0 | 298 | 1 | 1.1128 | 1 |
|  |  |  |  |  |  |  | 2362 | KPECFGP | 0 | 300 | 1 | 1.0938 | 1 |
|  |  |  |  |  |  |  | 2435 | LGRCAPE | 0 | 311 | 1 | 0.9768 | 0 |
|  |  |  |  |  |  |  | 2553 | RYLCAVL | 0 | 316 | 1 | 0.9918 | 0 |
|  |  |  |  |  |  |  | 2562 | ITKCAPL | 0 | 322 | 1 | 1.0665 | 1 |
|  |  |  |  |  |  |  | 2603 | IEDCLMA | 0 | 302 | 1 | 1.0505 | 1 |
|  |  |  |  |  |  |  | 2608 | MALCRYI | 0 | 285 | 0 | 0.9638 | 0 |
|  |  |  |  |  |  |  | 2648 | YERCWKY | 0 | 276 | 0 | 0.8770 | 0 |
|  |  |  |  |  |  |  | 2653 | KYYCLPT | 0 | 291 | 0 | 0.9868 | 0 |
|  |  |  |  |  |  |  | 2699 | AMPCLCA | 0 | 294 | 0 | 0.7678 | 0 |
|  |  |  |  |  |  |  | 2701 | PCLCAIA | 0 | 297 | 1 | 0.8468 | 0 |
|  |  |  |  |  |  |  | 3011 | TNHCLYF | 0 | 275 | 0 | 0.8593 | 0 |
|  |  |  |  |  |  |  | 3041 | SLFCKLA | 0 | 314 | 1 | 0.8908 | 0 |
|  |  |  |  |  |  |  | 3064 | VVNCLHI | 0 | 288 | 0 | 0.9945 | 0 |
|  |  |  |  |  |  |  | 3162 | QVSCYRT | 0 | 286 | 0 | 1.0095 | 1 |
|  |  |  |  |  |  |  | 3167 | RTLCSIY | 0 | 313 | 1 | 0.9713 | 0 |
|  |  |  |  |  |  |  | 3190 | LGECLAR | 0 | 311 | 1 | 1.0368 | 1 |
|  |  |  |  |  |  |  | 3213 | YNACSVY | 0 | 304 | 1 | 1.0583 | 1 |
|  |  |  |  |  |  |  | 3237 | EEMCPDI | 0 | 289 | 0 | 1.1140 | 1 |
|  |  |  |  |  |  |  | 3275 | PMLCSYL | 0 | 308 | 1 | 0.8595 | 0 |
|  |  |  |  |  |  |  | 3301 | PPPCTAV | 0 | 297 | 1 | 1.1255 | 1 |
|  |  |  |  |  |  |  | 3399 | SVLCRDL | 0 | 318 | 1 | 0.9198 | 0 |
|  |  |  |  |  |  |  | 3479 | AIGCAGV | 0 | 307 | 1 | 1.1258 | 1 |
|  |  |  |  |  |  |  | 3524 | LNMCAPT | 0 | 284 | 0 | 1.0773 | 1 |
|  |  |  |  |  |  |  | **3634** | **VVACFRM** | 1 | 308 | 1 | 1.0885 | 1 |
|  |  |  |  |  |  |  | 3649 | HRACNMF | 0 | 276 | 0 | 0.9755 | 0 |
|  |  |  |  |  |  |  | 3732 | AKSCHLE | 0 | 319 | 1 | 0.9243 | 0 |
|  |  |  |  |  |  |  | 3787 | ISACKGE | 0 | 319 | 1 | 1.0450 | 1 |
|  |  |  |  |  |  |  | 3840 | MQTCRWP | 0 | 260 | 0 | 0.8780 | 0 |
|  |  |  |  |  |  |  | 3895 | QLLCEGH | 0 | 305 | 1 | 1.0253 | 1 |
|  |  |  |  |  |  |  | 3921 | IIICTVD | 0 | 295 | 1 | 1.1275 | 1 |
|  |  |  |  |  |  |  | 3976 | QGPCTGN | 0 | 295 | 1 | 1.0885 | 1 |
|  |  |  |  |  |  |  | 4117 | LLSCSEA | 0 | 319 | 1 | 0.9743 | 0 |
|  |  |  |  |  |  |  | 4128 | MINCEEF | 0 | 275 | 0 | 1.1793 | 1 |
|  |  |  |  |  |  |  | 4241 | VSFCEDT | 0 | 286 | 0 | 1.0043 | 1 |
|  |  |  |  |  |  |  | 4643 | ALRCLSL | 0 | 320 | 1 | 0.8073 | 0 |
|  |  |  |  |  |  |  | 4655 | AFLCIIG | 0 | 303 | 1 | 0.9978 | 0 |
|  |  |  |  |  |  |  | 4661 | GYNCLKV | 0 | 292 | 0 | 0.9475 | 0 |
|  |  |  |  |  |  |  | 4874 | DMKCDDM | 0 | 284 | 0 | 0.9438 | 0 |
|  |  |  |  |  |  |  | 4880 | MMTCYLF | 0 | 275 | 0 | 0.9395 | 0 |
|  |  |  |  |  |  |  | 4956 | ETKCFIC | 0 | 294 | 0 | 1.0048 | 1 |
|  |  |  |  |  |  |  | 4959 | CFICGIG | 0 | 290 | 0 | 1.1048 | 1 |
|  |  |  |  |  |  |  | 5016 | QERCWDF | 0 | 272 | 0 | 0.8870 | 0 |
|  |  |  |  |  |  |  | 5025 | AGDCFRK | 0 | 313 | 1 | 1.1118 | 1 |
| **4** | Sarco/endoplasmic reticulum calcium ATPase (SERCA) pump | Sus scrofa, Oryctolagus cuniculus | P11607  P20647 | [1] | 29 | 7 | 70 | LAACISF | 0 | 305 | 1 | 1.1290 | 1 |
|  |  |  |  |  |  |  | **268** | **SLICIAV** | 1 | 303 | 1 | 1.0345 | 1 |
|  |  |  |  |  |  |  | 318 | ITTCLAL | 0 | 327 | 1 | 1.0190 | 1 |
|  |  |  |  |  |  |  | 344 | TLGCTSV | 0 | 304 | 1 | 1.0640 | 1 |
|  |  |  |  |  |  |  | 349 | SVICSDK | 0 | 314 | 1 | 0.9803 | 0 |
|  |  |  |  |  |  |  | 364 | MSVCRMF | 0 | 265 | 0 | 0.8898 | 0 |
|  |  |  |  |  |  |  | 377 | GDTCSLN | 0 | 311 | 1 | 1.0360 | 1 |
|  |  |  |  |  |  |  | 404 | PVKCHQY | 0 | 280 | 0 | 0.8868 | 0 |
|  |  |  |  |  |  |  | 417 | ATICALC | 0 | 320 | 1 | 1.0660 | 1 |
|  |  |  |  |  |  |  | 420 | CALCNDS | 0 | 298 | 1 | 1.0613 | 1 |
|  |  |  |  |  |  |  | 447 | ALTCLVE | 0 | 319 | 1 | 0.9883 | 0 |
|  |  |  |  |  |  |  | 471 | ANACNSV | 0 | 303 | 1 | 1.0865 | 1 |
|  |  |  |  |  |  |  | **498** | **SVYCTPN** | 1 | 296 | 1 | 1.1168 | 1 |
|  |  |  |  |  |  |  | **524** | **IDRCTHI** | 1 | 278 | 0 | 1.0043 | 1 |
|  |  |  |  |  |  |  | **560** | **TLRCLAL** | 1 | 316 | 1 | 0.8440 | 0 |
|  |  |  |  |  |  |  | 595 | FVGCVGM | 0 | 300 | 1 | 1.1415 | 1 |
|  |  |  |  |  |  |  | **613** | **VKLCRQA** | 1 | 317 | 1 | 0.9423 | 0 |
|  |  |  |  |  |  |  | 635 | VAICRRI | 0 | 291 | 0 | 1.0665 | 1 |
|  |  |  |  |  |  |  | **669** | **REACLNA** | 1 | 319 | 1 | 1.0080 | 1 |
|  |  |  |  |  |  |  | **674** | **NARCFAR** | 1 | 293 | 0 | 0.9688 | 0 |
|  |  |  |  |  |  |  | 773 | EVVCIFL | 0 | 300 | 1 | 1.0215 | 1 |
|  |  |  |  |  |  |  | 841 | AIGCYVG | 0 | 306 | 1 | 1.0975 | 1 |
|  |  |  |  |  |  |  | 875 | FLQCKED | 0 | 294 | 0 | 1.0015 | 1 |
|  |  |  |  |  |  |  | 887 | GVDCAVF | 0 | 296 | 1 | 1.1760 | 1 |
|  |  |  |  |  |  |  | 909 | IEMCNAL | 0 | 296 | 1 | 1.0518 | 1 |
|  |  |  |  |  |  |  | 937 | GSICLSM | 0 | 302 | 1 | 0.9618 | 0 |
|  |  |  |  |  |  |  | 997 | GKECVQP | 0 | 302 | 1 | 1.1330 | 1 |
|  |  |  |  |  |  |  | 1005 | TKSCSFS | 0 | 307 | 1 | 0.9730 | 0 |
|  |  |  |  |  |  |  | 1010 | FSACTDG | 0 | 308 | 1 | 1.0883 | 1 |
| **5** | H-Ras | Homo sapiens | P01112 | [1] | 6 | 1 | 51 | GETCLLD | 0 | 315 | 1 | 0.9858 | 0 |
|  |  |  |  |  |  |  | 80 | GFLCVFA | 0 | 311 | 1 | 1.0055 | 1 |
|  |  |  |  |  |  |  | **118** | **GNKCDLA** | 1 | 311 | 1 | 0.9748 | 0 |
|  |  |  |  |  |  |  | 181 | GPGCMSC | 0 | 300 | 1 | 1.0585 | 1 |
|  |  |  |  |  |  |  | 184 | CMSCKCV | 0 | 267 | 0 | 0.7543 | 0 |
|  |  |  |  |  |  |  | 186 | SCKCVLS | 0 | 308 | 1 | 0.8040 | 0 |
| **6** | c-Jun (AP-1) | Homo sapiens | P05412 | [1] | 3 | 1 | 99 | QFLCPKN | 0 | 307 | 1 | 1.0513 | 1 |
|  |  |  |  |  |  |  | **269** | **ASKCRKR** | 1 | 298 | 1 | 0.8708 | 0 |
|  |  |  |  |  |  |  | 320 | NSGCQLM | 0 | 291 | 0 | 0.9710 | 0 |
| **7** | IκB kinase (IKK-β) | Mus musculus | O88351 | [1] | 20 | 1 | 12 | TQTCGAW | 0 | 291 | 0 | 1.0683 | 1 |
|  |  |  |  |  |  |  | 46 | IKQCRQE | 0 | 296 | 1 | 0.9715 | 0 |
|  |  |  |  |  |  |  | 59 | NRWCLEI | 0 | 278 | 0 | 0.8350 | 0 |
|  |  |  |  |  |  |  | 99 | MEYCQGG | 0 | 275 | 0 | 0.9775 | 0 |
|  |  |  |  |  |  |  | 114 | FENCCGL | 0 | 291 | 0 | 0.9303 | 0 |
|  |  |  |  |  |  |  | 115 | ENCCGLR | 0 | 306 | 1 | 0.9023 | 0 |
|  |  |  |  |  |  |  | **179** | **GSLCTSF** | 1 | 305 | 1 | 0.9770 | 0 |
|  |  |  |  |  |  |  | 215 | AFECITG | 0 | 300 | 1 | 1.0898 | 1 |
|  |  |  |  |  |  |  | 299 | PNGCFRA | 0 | 299 | 1 | 1.0885 | 1 |
|  |  |  |  |  |  |  | 370 | ATQCISD | 0 | 305 | 1 | 1.0145 | 1 |
|  |  |  |  |  |  |  | 412 | SVSCILQ | 0 | 301 | 1 | 1.0100 | 1 |
|  |  |  |  |  |  |  | 444 | KEDCNRL | 0 | 302 | 1 | 1.1743 | 1 |
|  |  |  |  |  |  |  | 464 | NNSCLSK | 0 | 299 | 1 | 0.9593 | 0 |
|  |  |  |  |  |  |  | 524 | VEQCGRE | 0 | 311 | 1 | 1.0980 | 1 |
|  |  |  |  |  |  |  | 618 | TVVCKQK | 0 | 302 | 1 | 0.9925 | 0 |
|  |  |  |  |  |  |  | 662 | KIACSKV | 0 | 313 | 1 | 1.0128 | 1 |
|  |  |  |  |  |  |  | 695 | SSACDSL | 0 | 322 | 1 | 1.0618 | 1 |
|  |  |  |  |  |  |  | 716 | HALCSRL | 0 | 318 | 1 | 1.0015 | 1 |
|  |  |  |  |  |  |  | 750 | EERCSLE | 0 | 322 | 1 | 0.8420 | 0 |
|  |  |  |  |  |  |  | 756 | EQACD-- | 0 | 289 | 0 | 1.1043 | 1 |
| **8** | Protein kinase C-a (PKC-alpha) | Rattus norvegicus | P05696 | [1] | 20 | 1 | 50 | PTFCSHC | 0 | 293 | 0 | 0.9025 | 0 |
|  |  |  |  |  |  |  | 53 | CSHCTDF | 0 | 270 | 0 | 0.9288 | 0 |
|  |  |  |  |  |  |  | 67 | GFQCQVC | 0 | 268 | 0 | 0.9533 | 0 |
|  |  |  |  |  |  |  | 70 | CQVCCFV | 0 | 258 | 0 | 0.8305 | 0 |
|  |  |  |  |  |  |  | 71 | QVCCFVV | 0 | 280 | 0 | 0.8940 | 0 |
|  |  |  |  |  |  |  | 78 | HKRCHEF | 0 | 279 | 0 | 0.8758 | 0 |
|  |  |  |  |  |  |  | 86 | TFSCPGA | 0 | 302 | 1 | 1.1170 | 1 |
|  |  |  |  |  |  |  | 115 | PTFCDHC | 0 | 287 | 0 | 0.9860 | 0 |
|  |  |  |  |  |  |  | 118 | CDHCGSL | 0 | 298 | 1 | 1.0390 | 1 |
|  |  |  |  |  |  |  | 132 | GMKCDTC | 0 | 289 | 0 | 0.9885 | 0 |
|  |  |  |  |  |  |  | 135 | CDTCDMN | 0 | 273 | 0 | 1.0970 | 1 |
|  |  |  |  |  |  |  | 143 | HKQCVIN | 0 | 280 | 0 | 1.1025 | 1 |
|  |  |  |  |  |  |  | 151 | PSLCGMD | 0 | 307 | 1 | 0.9708 | 0 |
|  |  |  |  |  |  |  | 380 | DVECTMV | 0 | 281 | 0 | 1.0683 | 1 |
|  |  |  |  |  |  |  | 406 | LHSCFQT | 0 | 287 | 0 | 0.9868 | 0 |
|  |  |  |  |  |  |  | 485 | FGMCKEH | 0 | 285 | 0 | 1.0088 | 1 |
|  |  |  |  |  |  |  | **499** | **RTFCGTP** | 1 | 319 | 1 | 1.0735 | 1 |
|  |  |  |  |  |  |  | 569 | VSICKGL | 0 | 309 | 1 | 1.0095 | 1 |
|  |  |  |  |  |  |  | 583 | RLGCGPE | 0 | 330 | 1 | 1.1580 | 1 |
|  |  |  |  |  |  |  | 619 | PKVCGKG | 0 | 314 | 1 | 1.0568 | 1 |
| **9** | Actin Beta, Gamma1 | Homo sapiens | P60709  P63261 | [1] [2] | 6 | 2 | 17 | SGMCKAG | 0 | 304 | 1 | 0.9758 | 0 |
|  |  |  |  |  |  |  | **217** | **EKLCYVA** | 1 | 319 | 1 | 1.0015 | 1 |
|  |  |  |  |  |  |  | 257 | RFRCPEA | 0 | 302 | 1 | 1.0383 | 1 |
|  |  |  |  |  |  |  | 272 | MESCGIH | 0 | 285 | 0 | 1.1205 | 1 |
|  |  |  |  |  |  |  | 285 | IMKCDVD | 0 | 289 | 0 | 0.9870 | 0 |
|  |  |  |  |  |  |  | **374** | **HRKCF--** | 1 | 267 | 0 | 0.8240 | 0 |
| **10** | Mitochondrial NADP-dependent isocitrate dehydrogenase | Homo sapiens | O75874 | [1] | 5 | 1 | 73 | GVKCATI | 0 | 302 | 1 | 1.0308 | 1 |
|  |  |  |  |  |  |  | 114 | AIICKNI | 0 | 292 | 0 | 0.9798 | 0 |
|  |  |  |  |  |  |  | **269** | **IWACKNY** | 1 | 288 | 0 | 0.8960 | 0 |
|  |  |  |  |  |  |  | 297 | VLVCPDG | 0 | 308 | 1 | 1.0795 | 1 |
|  |  |  |  |  |  |  | 379 | LAACIKG | 0 | 314 | 1 | 1.1320 | 1 |
| **11** | PTEN | Homo sapiens | P60484 | [1] | 10 | 1 | 71 | YNLCAER | 0 | 307 | 1 | 1.0565 | 1 |
|  |  |  |  |  |  |  | 83 | KFNCRVA | 0 | 290 | 0 | 1.0093 | 1 |
|  |  |  |  |  |  |  | 105 | KPFCEDL | 0 | 306 | 1 | 1.0505 | 1 |
|  |  |  |  |  |  |  | **124** | **AIHCKAG** | 1 | 306 | 1 | 0.9215 | 0 |
|  |  |  |  |  |  |  | 136 | VMICAYL | 0 | 296 | 1 | 1.0053 | 1 |
|  |  |  |  |  |  |  | 211 | GGTCNPQ | 0 | 293 | 0 | 1.1030 | 1 |
|  |  |  |  |  |  |  | 218 | FVVCQLK | 0 | 299 | 1 | 0.9245 | 0 |
|  |  |  |  |  |  |  | 250 | LPVCGDI | 0 | 309 | 1 | 1.0785 | 1 |
|  |  |  |  |  |  |  | 296 | GSLCDQE | 0 | 317 | 1 | 0.9890 | 0 |
|  |  |  |  |  |  |  | 304 | DSICSIE | 0 | 300 | 1 | 0.9958 | 0 |
| **12** | Protein kinase B (Akt, PKB) | Homo sapiens | P31749 | [3] | 7 | 2 | 60 | VAQCQLM | 0 | 286 | 0 | 0.9850 | 0 |
|  |  |  |  |  |  |  | 77 | IIRCLQW | 0 | 283 | 0 | 0.8763 | 0 |
|  |  |  |  |  |  |  | 224 | DRLCFVM | 0 | 297 | 1 | 0.9175 | 0 |
|  |  |  |  |  |  |  | **296** | **FGLCKEG** | 1 | 318 | 1 | 0.9980 | 0 |
|  |  |  |  |  |  |  | **310** | **KTFCGTP** | 1 | 320 | 1 | 1.0735 | 1 |
|  |  |  |  |  |  |  | 344 | EMMCGRL | 0 | 302 | 1 | 1.0215 | 1 |
|  |  |  |  |  |  |  | 460 | SMECVDS | 0 | 295 | 1 | 1.0678 | 1 |
| **13** | Hemoglobin | Homo sapiens | P68871 | [4] | 2 | 1 | **94** | **ELHCDKL** | 1 | 308 | 1 | 0.8933 | 0 |
|  |  |  |  |  |  |  | 113 | VLVCVLA | 0 | 319 | 1 | 1.0038 | 1 |
| **14** | p65 (NF-kB) | Homo sapiens | Q04206 | [5] | 9 | 1 | **38** | **RYKCEGR** | 1 | 295 | 1 | 0.9953 | 0 |
|  |  |  |  |  |  |  | 95 | GKDCRDG | 0 | 305 | 1 | 1.0620 | 1 |
|  |  |  |  |  |  |  | 105 | AELCPDR | 0 | 321 | 1 | 1.1033 | 1 |
|  |  |  |  |  |  |  | 109 | PDRCIHS | 0 | 283 | 0 | 0.9743 | 0 |
|  |  |  |  |  |  |  | 120 | GIQCVKK | 0 | 297 | 1 | 1.0495 | 1 |
|  |  |  |  |  |  |  | 160 | VRLCFQV | 0 | 299 | 1 | 0.8868 | 0 |
|  |  |  |  |  |  |  | 197 | LKICRVN | 0 | 303 | 1 | 1.0358 | 1 |
|  |  |  |  |  |  |  | 206 | SGSCLGG | 0 | 313 | 1 | 0.9815 | 0 |
|  |  |  |  |  |  |  | 216 | FLLCDKV | 0 | 307 | 1 | 0.9545 | 0 |
| **15** | p50 (NF-kB) | Homo sapiens | P19838 | [5] | 11 | 1 | **61** | **RYVCEGP** | 1 | 295 | 1 | 1.0378 | 1 |
|  |  |  |  |  |  |  | 87 | VKICNYV | 0 | 287 | 0 | 1.0595 | 1 |
|  |  |  |  |  |  |  | 118 | GKHCEDG | 0 | 302 | 1 | 0.9913 | 0 |
|  |  |  |  |  |  |  | 123 | DGICTVT | 0 | 298 | 1 | 1.1098 | 1 |
|  |  |  |  |  |  |  | 161 | TEACIRG | 0 | 310 | 1 | 1.1228 | 1 |
|  |  |  |  |  |  |  | 261 | TAGCVTG | 0 | 312 | 1 | 1.2263 | 1 |
|  |  |  |  |  |  |  | 272 | YLLCDKV | 0 | 310 | 1 | 0.9545 | 0 |
|  |  |  |  |  |  |  | 446 | PEGCDKS | 0 | 310 | 1 | 1.1053 | 1 |
|  |  |  |  |  |  |  | 666 | SLPCLLL | 0 | 322 | 1 | 0.9545 | 0 |
|  |  |  |  |  |  |  | 703 | LAGCLLL | 0 | 331 | 1 | 1.0838 | 1 |
|  |  |  |  |  |  |  | 925 | DSVCDSG | 0 | 297 | 1 | 0.9915 | 0 |
| **16** | Caspase-1 (p10) | Homo sapiens | P29466 | [5] [6] [7] | 12 | 2 | 69 | AQACQIC | 0 | 281 | 0 | 1.0165 | 1 |
|  |  |  |  |  |  |  | 72 | CQICITY | 0 | 271 | 0 | 1.0628 | 1 |
|  |  |  |  |  |  |  | 77 | TYICEED | 0 | 297 | 1 | 1.1100 | 1 |
|  |  |  |  |  |  |  | 136 | VKLCSLE | 0 | 340 | 1 | 0.9165 | 0 |
|  |  |  |  |  |  |  | 169 | LIICNEE | 0 | 302 | 1 | 1.1285 | 1 |
|  |  |  |  |  |  |  | 244 | EGICGKK | 0 | 324 | 1 | 1.0708 | 1 |
|  |  |  |  |  |  |  | 270 | TKNCPSL | 0 | 308 | 1 | 1.1580 | 1 |
|  |  |  |  |  |  |  | 285 | IQACRGD | 0 | 299 | 1 | 1.0080 | 1 |
|  |  |  |  |  |  |  | 331 | IAFCSST | 0 | 296 | 1 | 0.9500 | 0 |
|  |  |  |  |  |  |  | **362** | **EYACSCD** | 1 | 309 | 1 | 0.7723 | 0 |
|  |  |  |  |  |  |  | 364 | ACSCDVE | 0 | 303 | 1 | 0.8870 | 0 |
|  |  |  |  |  |  |  | **397** | **LTRCFYL** | 1 | 308 | 1 | 0.8843 | 0 |
| **17** | STAT3 | Homo sapiens | P40763 | [5] | 14 | 2 | 108 | VARCLWE | 0 | 297 | 1 | 0.8585 | 0 |
|  |  |  |  |  |  |  | 251 | QIACIGG | 0 | 305 | 1 | 1.1090 | 1 |
|  |  |  |  |  |  |  | 259 | PNICLDR | 0 | 294 | 0 | 0.9985 | 0 |
|  |  |  |  |  |  |  | **328** | **RQPCMPM** | 1 | 276 | 0 | 1.0538 | 1 |
|  |  |  |  |  |  |  | 367 | IKVCIDK | 0 | 302 | 1 | 1.0225 | 1 |
|  |  |  |  |  |  |  | 418 | EQRCGNG | 0 | 294 | 0 | 0.8778 | 0 |
|  |  |  |  |  |  |  | 426 | RANCDAS | 0 | 305 | 1 | 1.1570 | 1 |
|  |  |  |  |  |  |  | 468 | SNICQMP | 0 | 276 | 0 | 0.9360 | 0 |
|  |  |  |  |  |  |  | **542** | **YSGCQIT** | 1 | 278 | 0 | 1.0220 | 1 |
|  |  |  |  |  |  |  | 550 | AKFCKEN | 0 | 309 | 1 | 1.0050 | 1 |
|  |  |  |  |  |  |  | 687 | GKYCRPE | 0 | 304 | 1 | 1.0320 | 1 |
|  |  |  |  |  |  |  | 712 | KFICVTP | 0 | 302 | 1 | 1.1040 | 1 |
|  |  |  |  |  |  |  | 718 | PTTCSNT | 0 | 301 | 1 | 0.9343 | 0 |
|  |  |  |  |  |  |  | 765 | TSECATS | 0 | 301 | 1 | 1.1080 | 1 |
| **18** | S100A8 | Homo sapiens | P05109 | [5] | 1 | 1 | **42** | **ETECPQY** | 1 | 304 | 1 | 1.2015 | 1 |
| **19** | S100A9 | Homo sapiens | P06702 | [5] | 1 | 1 | **3** | **-MTCKMS** | 1 | 275 | 0 | 0.9208 | 0 |
| **20** | GRX1 | Mus musculus | Q9QUH0 | [8] | 5 | 1 | 8 | FVNCKIQ | 0 | 277 | 0 | 1.0650 | 1 |
|  |  |  |  |  |  |  | **23** | **KPTCPYC** | 1 | 289 | 0 | 1.1395 | 1 |
|  |  |  |  |  |  |  | 26 | CPYCRKT | 0 | 277 | 0 | 0.9670 | 0 |
|  |  |  |  |  |  |  | 79 | GKDCIGG | 0 | 306 | 1 | 1.1460 | 1 |
|  |  |  |  |  |  |  | 83 | IGGCSDL | 0 | 322 | 1 | 1.0058 | 1 |
| **21** | Fas (CD95) | Mus musculus | P25446 | [8], [9] | 24 | 1 | 44 | DKNCSEG | 0 | 307 | 1 | 1.0573 | 1 |
|  |  |  |  |  |  |  | 55 | GPFCCQP | 0 | 289 | 0 | 0.8433 | 0 |
|  |  |  |  |  |  |  | 56 | PFCCQPC | 0 | 265 | 0 | 0.8328 | 0 |
|  |  |  |  |  |  |  | 59 | CQPCQPG | 0 | 266 | 0 | 1.0105 | 1 |
|  |  |  |  |  |  |  | 69 | VEDCKMN | 0 | 295 | 1 | 1.0660 | 1 |
|  |  |  |  |  |  |  | 78 | TPTCAPC | 0 | 293 | 0 | 1.1340 | 1 |
|  |  |  |  |  |  |  | 81 | CAPCTEG | 0 | 289 | 0 | 1.1960 | 1 |
|  |  |  |  |  |  |  | 97 | ADKCRRC | 0 | 288 | 0 | 1.0145 | 1 |
|  |  |  |  |  |  |  | 100 | CRRCTLC | 0 | 278 | 0 | 0.8325 | 0 |
|  |  |  |  |  |  |  | 103 | CTLCDEE | 0 | 323 | 1 | 1.0715 | 1 |
|  |  |  |  |  |  |  | 115 | ETNCTLT | 0 | 313 | 1 | 1.0903 | 1 |
|  |  |  |  |  |  |  | 123 | NTKCKCK | 0 | 304 | 1 | 0.7598 | 0 |
|  |  |  |  |  |  |  | 125 | KCKCKPD | 0 | 294 | 0 | 0.8105 | 0 |
|  |  |  |  |  |  |  | 131 | DFYCDSP | 0 | 285 | 0 | 0.9968 | 0 |
|  |  |  |  |  |  |  | 136 | SPGCEHC | 0 | 298 | 1 | 1.1483 | 1 |
|  |  |  |  |  |  |  | 139 | CEHCVRC | 0 | 274 | 0 | 1.0020 | 1 |
|  |  |  |  |  |  |  | 142 | CVRCASC | 0 | 277 | 0 | 0.9078 | 0 |
|  |  |  |  |  |  |  | 145 | CASCEHG | 0 | 286 | 0 | 1.1233 | 1 |
|  |  |  |  |  |  |  | 153 | LEPCTAT | 0 | 300 | 1 | 1.1190 | 1 |
|  |  |  |  |  |  |  | 161 | NTNCRKQ | 0 | 291 | 0 | 0.9965 | 0 |
|  |  |  |  |  |  |  | 194 | KRKCWKR | 0 | 282 | 0 | 0.8358 | 0 |
|  |  |  |  |  |  |  | 272 | LLLCWYQ | 0 | 294 | 0 | 0.8998 | 0 |
|  |  |  |  |  |  |  | **295** | **KAECRRT** | 1 | 289 | 0 | 1.0910 | 1 |
|  |  |  |  |  |  |  | 325 | EGQCLE- | 0 | 297 | 1 | 1.0118 | 1 |
| **22** | Acylphosphatase-2 | Oryctolagus cuniculus | P00820 | [10] | 1 | 1 | **22** | **QGVCFRM** | 1 | 291 | 0 | 1.0075 | 1 |
| **23** | Chloride intracellular channel protein 1 | Homo sapiens | O00299 | [11] | 6 | 1 | **24** | **IGNCPFS** | 1 | 298 | 1 | 1.1593 | 1 |
|  |  |  |  |  |  |  | 59 | QKLCPGG | 0 | 319 | 1 | 1.1173 | 1 |
|  |  |  |  |  |  |  | 89 | AVLCPPR | 0 | 323 | 1 | 1.1548 | 1 |
|  |  |  |  |  |  |  | 178 | LADCNLL | 0 | 312 | 1 | 1.1825 | 1 |
|  |  |  |  |  |  |  | 191 | QVVCKKY | 0 | 289 | 0 | 0.9700 | 0 |
|  |  |  |  |  |  |  | 223 | ASTCPDD | 0 | 302 | 1 | 1.1085 | 1 |
| **24** | Beta-crystallin A3 | Rattus norvegicus | P14881 | Uniprot | 8 | 2 | 52 | TSSCPNV | 0 | 285 | 0 | 1.0580 | 1 |
|  |  |  |  |  |  |  | 70 | KVECGAW | 0 | 304 | 1 | 1.1398 | 1 |
|  |  |  |  |  |  |  | **82** | **TSFCGQQ** | 1 | 292 | 0 | 1.0030 | 1 |
|  |  |  |  |  |  |  | **117** | **RPICSAN** | 1 | 318 | 1 | 1.0258 | 1 |
|  |  |  |  |  |  |  | 142 | WEICDDY | 0 | 285 | 0 | 1.0790 | 1 |
|  |  |  |  |  |  |  | 165 | KIQCGAW | 0 | 295 | 1 | 1.0888 | 1 |
|  |  |  |  |  |  |  | 170 | AWVCYQY | 0 | 280 | 0 | 0.8765 | 0 |
|  |  |  |  |  |  |  | 185 | ILECDHH | 0 | 286 | 0 | 1.0368 | 1 |
| **25** | Lactoylglutathione lyase | Homo sapiens | Q04760 | Uniprot | 4 | 1 | 19 | ALSCCSD | 0 | 301 | 1 | 0.8098 | 0 |
|  |  |  |  |  |  |  | 20 | LSCCSDA | 0 | 300 | 1 | 0.7783 | 0 |
|  |  |  |  |  |  |  | 61 | IQKCDFP | 0 | 282 | 0 | 0.9663 | 0 |
|  |  |  |  |  |  |  | **139** | **YSACKRF** | 1 | 302 | 1 | 1.0505 | 1 |
| **26** | Selenoprotein W | Homo sapiens | P63302 | Uniprot | 2 | 1 | 10 | VVYCGAU | 0 | 306 | 1 | 1.0880 | 1 |
|  |  |  |  |  |  |  | **37** | **LDICGEG** | 1 | 315 | 1 | 1.2328 | 1 |
| **27** | Hydrogen peroxide-inducible genes activator | Escherichia coli | P0ACQ4 | [12] | 6 | 1 | 25 | ADSCHVS | 0 | 298 | 1 | 1.0530 | 1 |
|  |  |  |  |  |  |  | 143 | KLDCVIL | 0 | 305 | 1 | 1.1590 | 1 |
|  |  |  |  |  |  |  | 180 | NRECVPM | 0 | 284 | 0 | 1.0860 | 1 |
|  |  |  |  |  |  |  | **199** | **DGHCLRD** | 1 | 298 | 1 | 0.8893 | 0 |
|  |  |  |  |  |  |  | 208 | MGFCFEA | 0 | 293 | 0 | 0.9978 | 0 |
|  |  |  |  |  |  |  | 259 | YLPCIKP | 0 | 291 | 0 | 1.0035 | 1 |
| **28** | Carbonic anhydrase 3 | Mus musculus | P16015 | [13] | 5 | 2 | 66 | GKTCRVV | 0 | 295 | 1 | 1.0180 | 1 |
|  |  |  |  |  |  |  | **182** | **DPSCLFP** | 1 | 300 | 1 | 1.0038 | 1 |
|  |  |  |  |  |  |  | **187** | **FPACRDY** | 1 | 302 | 1 | 1.0398 | 1 |
|  |  |  |  |  |  |  | 202 | TPPCEEC | 0 | 290 | 0 | 1.1865 | 1 |
|  |  |  |  |  |  |  | 205 | CEECIVW | 0 | 270 | 0 | 1.1303 | 1 |
| **29** | Annexin A2 | Rattus norvegicus | Q07936 | [14] | 5 | 2 | **9** | **EILCKLS** | 1 | 327 | 1 | 0.9480 | 0 |
|  |  |  |  |  |  |  | **133** | **EIICSRT** | 1 | 302 | 1 | 1.0120 | 1 |
|  |  |  |  |  |  |  | 223 | RSVCHLQ | 0 | 292 | 0 | 0.8813 | 0 |
|  |  |  |  |  |  |  | 262 | LVQCIQN | 0 | 287 | 0 | 1.0263 | 1 |
|  |  |  |  |  |  |  | 335 | LYLCGGD | 0 | 321 | 1 | 0.9960 | 0 |
| **30** | Nitric oxide synthase, endothelial | Homo sapiens | P29474 | [15] | 29 | 2 | 15 | GPPCGLG | 0 | 324 | 1 | 1.1050 | 1 |
|  |  |  |  |  |  |  | 26 | LGLCGKQ | 0 | 324 | 1 | 1.0080 | 1 |
|  |  |  |  |  |  |  | 94 | DGPCTPR | 0 | 296 | 1 | 1.1360 | 1 |
|  |  |  |  |  |  |  | 99 | PRRCLGS | 0 | 298 | 1 | 0.7848 | 0 |
|  |  |  |  |  |  |  | 184 | APRCVGR | 0 | 307 | 1 | 0.9845 | 0 |
|  |  |  |  |  |  |  | 201 | ARDCRSA | 0 | 304 | 1 | 0.9478 | 0 |
|  |  |  |  |  |  |  | 212 | TYICNHI | 0 | 275 | 0 | 0.9978 | 0 |
|  |  |  |  |  |  |  | 235 | PQRCPGR | 0 | 284 | 0 | 0.9983 | 0 |
|  |  |  |  |  |  |  | 274 | TELCIQH | 0 | 297 | 1 | 1.0123 | 1 |
|  |  |  |  |  |  |  | 368 | RNLCDPH | 0 | 300 | 1 | 1.0670 | 1 |
|  |  |  |  |  |  |  | 382 | VAVCMDL | 0 | 303 | 1 | 1.0380 | 1 |
|  |  |  |  |  |  |  | 441 | RGGCPAD | 0 | 318 | 1 | 1.2000 | 1 |
|  |  |  |  |  |  |  | 552 | RVLCMDE | 0 | 314 | 1 | 0.9508 | 0 |
|  |  |  |  |  |  |  | 618 | SISCSDP | 0 | 302 | 1 | 0.9610 | 0 |
|  |  |  |  |  |  |  | 648 | LRFCVFG | 0 | 287 | 0 | 0.9273 | 0 |
|  |  |  |  |  |  |  | 661 | PHFCAFA | 0 | 284 | 0 | 0.9680 | 0 |
|  |  |  |  |  |  |  | **689** | **DELCGQE** | 1 | 328 | 1 | 1.0565 | 1 |
|  |  |  |  |  |  |  | 707 | QAACETF | 0 | 307 | 1 | 1.2448 | 1 |
|  |  |  |  |  |  |  | 711 | ETFCVGE | 0 | 317 | 1 | 1.0425 | 1 |
|  |  |  |  |  |  |  | 802 | IGVCPPN | 0 | 307 | 1 | 1.1720 | 1 |
|  |  |  |  |  |  |  | 853 | LPPCTLR | 0 | 308 | 1 | 1.0908 | 1 |
|  |  |  |  |  |  |  | **908** | **WFRCPTL** | 1 | 297 | 1 | 1.0263 | 1 |
|  |  |  |  |  |  |  | 976 | YGVCSTW | 0 | 301 | 1 | 0.9795 | 0 |
|  |  |  |  |  |  |  | 991 | PVPCFIR | 0 | 279 | 0 | 1.0710 | 1 |
|  |  |  |  |  |  |  | 1009 | SLPCILV | 0 | 299 | 1 | 1.0025 | 1 |
|  |  |  |  |  |  |  | 1048 | VFGCRCS | 0 | 295 | 1 | 0.8005 | 0 |
|  |  |  |  |  |  |  | 1050 | GCRCSQL | 0 | 301 | 1 | 0.6565 | 0 |
|  |  |  |  |  |  |  | 1105 | RVLCLER | 0 | 319 | 1 | 0.9933 | 0 |
|  |  |  |  |  |  |  | 1114 | MFVCGDV | 0 | 290 | 0 | 1.0300 | 1 |
| **31** | Troponin I, fast skeletal muscle | Homo sapiens | P48788 | [16] | 3 | 1 | 49 | AEHCPPL | 0 | 315 | 1 | 1.1088 | 1 |
|  |  |  |  |  |  |  | 65 | QELCKQL | 0 | 317 | 1 | 0.9798 | 0 |
|  |  |  |  |  |  |  | **134** | **HKVCMDL** | 1 | 298 | 1 | 0.9888 | 0 |
| **32** | Complex 2 | Homo sapiens | O14521 | [17] | 5 | 1 | 11 | SAVCGAL | 0 | 327 | 1 | 1.1398 | 1 |
|  |  |  |  |  |  |  | 44 | PEWCGVQ | 0 | 291 | 0 | 1.0203 | 1 |
|  |  |  |  |  |  |  | **88** | **LNPCSAM** | 1 | 297 | 1 | 1.0060 | 1 |
|  |  |  |  |  |  |  | 140 | AGLCYFN | 0 | 312 | 1 | 0.9465 | 0 |
|  |  |  |  |  |  |  | 150 | VGICKAV | 0 | 308 | 1 | 1.0278 | 1 |
| **33** | NAD-dependent protein deacetylase sirtuin-1 | Homo sapiens | Q96EB6 | [18] [19] | 19 | 3 | **67** | **ARGCPGA** | 1 | 317 | 1 | 1.1150 | 1 |
|  |  |  |  |  |  |  | 160 | FHSCESD | 0 | 289 | 0 | 1.0383 | 1 |
|  |  |  |  |  |  |  | 253 | LQECKKI | 0 | 282 | 0 | 1.0000 | 0 |
|  |  |  |  |  |  |  | 268 | SVSCGIP | 0 | 303 | 1 | 1.1053 | 1 |
|  |  |  |  |  |  |  | 326 | PSLCHKF | 0 | 290 | 0 | 0.8543 | 0 |
|  |  |  |  |  |  |  | 362 | IIQCHGS | 0 | 285 | 0 | 0.9443 | 0 |
|  |  |  |  |  |  |  | 371 | TASCLIC | 0 | 284 | 0 | 1.0723 | 1 |
|  |  |  |  |  |  |  | 374 | CLICKYK | 0 | 293 | 0 | 0.9630 | 0 |
|  |  |  |  |  |  |  | 380 | KVDCEAV | 0 | 303 | 1 | 1.1985 | 1 |
|  |  |  |  |  |  |  | 395 | VPRCPRC | 0 | 293 | 0 | 1.0548 | 1 |
|  |  |  |  |  |  |  | 398 | CPRCPAD | 0 | 297 | 1 | 1.0538 | 1 |
|  |  |  |  |  |  |  | **482** | **LGDCDVI** | 1 | 298 | 1 | 1.1658 | 1 |
|  |  |  |  |  |  |  | 490 | NELCHRL | 0 | 308 | 1 | 0.9288 | 0 |
|  |  |  |  |  |  |  | 501 | AKLCCNP | 0 | 307 | 1 | 0.7908 | 0 |
|  |  |  |  |  |  |  | 502 | KLCCNPV | 0 | 285 | 0 | 0.9175 | 0 |
|  |  |  |  |  |  |  | **574** | **SKGCMEE** | 1 | 318 | 1 | 1.1165 | 1 |
|  |  |  |  |  |  |  | 623 | VRKCWPN | 0 | 286 | 0 | 0.9125 | 0 |
|  |  |  |  |  |  |  | 671 | SSSCGSN | 0 | 310 | 1 | 1.0278 | 1 |
|  |  |  |  |  |  |  | 680 | SGTCQSP | 0 | 292 | 0 | 0.9288 | 0 |
| **34** | Elongation factor 1 alpha 1 | Homo sapiens | P68104 | [2] | 6 | 1 | 31 | IYKCGGI | 0 | 300 | 1 | 0.9815 | 0 |
|  |  |  |  |  |  |  | 111 | QADCAVL | 0 | 305 | 1 | 1.2353 | 1 |
|  |  |  |  |  |  |  | 234 | ALDCILP | 0 | 314 | 1 | 1.0693 | 1 |
|  |  |  |  |  |  |  | 363 | VLDCHTA | 0 | 306 | 1 | 1.0293 | 1 |
|  |  |  |  |  |  |  | 370 | HIACKFA | 0 | 303 | 1 | 1.0663 | 1 |
|  |  |  |  |  |  |  | **411** | **KPMCVES** | 1 | 304 | 1 | 1.1125 | 1 |
| **35** | Thioredoxin | Homo sapiens | P10599 | [20] | 5 | 1 | 32 | ATWCGPC | 0 | 309 | 1 | 1.0423 | 1 |
|  |  |  |  |  |  |  | 35 | CGPCKMI | 0 | 272 | 0 | 0.9733 | 0 |
|  |  |  |  |  |  |  | 62 | VDDCQDV | 0 | 280 | 0 | 1.1243 | 1 |
|  |  |  |  |  |  |  | 69 | ASECEVK | 0 | 304 | 1 | 1.1485 | 1 |
|  |  |  |  |  |  |  | **73** | **EVKCMPT** | 1 | 297 | 1 | 1.0030 | 1 |
| **36** | Neurogranin | Homo sapiens | Q92686 | [21] | 3 | 3 | **3** | **-MDCCTE** | 1 | 283 | 0 | 0.9570 | 0 |
|  |  |  |  |  |  |  | **4** | **MDCCTEN** | 1 | 280 | 0 | 1.0410 | 1 |
|  |  |  |  |  |  |  | **9** | **ENACSKP** | 1 | 316 | 1 | 1.0050 | 1 |
| **37** | Carbonic anhydrase III | Homo sapiens | P07451 | [2] [22] | 5 | 2 | 66 | GKTCRVV | 0 | 295 | 1 | 1.0180 | 1 |
|  |  |  |  |  |  |  | **182** | **DPSCLFP** | 1 | 300 | 1 | 1.0038 | 1 |
|  |  |  |  |  |  |  | **187** | **FPACRDY** | 1 | 302 | 1 | 1.0398 | 1 |
|  |  |  |  |  |  |  | 202 | TPPCEEC | 0 | 290 | 0 | 1.1865 | 1 |
|  |  |  |  |  |  |  | 205 | CEECIVW | 0 | 270 | 0 | 1.1303 | 1 |
| **38** | Heat shock 60 KD protein 1 (chaperonin) | Homo sapiens | P10809 | [2] | 3 | 1 | 237 | GQKCEFQ | 0 | 282 | 0 | 1.0078 | 1 |
|  |  |  |  |  |  |  | 442 | GGGCALL | 0 | 331 | 1 | 1.0778 | 1 |
|  |  |  |  |  |  |  | **447** | **LLRCIPA** | 1 | 303 | 1 | 0.9350 | 0 |
| **39** | Protein tyrosine phosphatase, non-receptor type 1 | Homo sapiens | P18031 | [1]  [23] | 10 | 1 | 32 | DFPCRVA | 0 | 288 | 0 | 1.0018 | 1 |
|  |  |  |  |  |  |  | 92 | PNTCGHF | 0 | 286 | 0 | 1.0580 | 1 |
|  |  |  |  |  |  |  | 121 | SLKCAQY | 0 | 297 | 1 | 0.9620 | 0 |
|  |  |  |  |  |  |  | **215** | **VVHCSAG** | 1 | 309 | 1 | 0.8800 | 0 |
|  |  |  |  |  |  |  | 226 | GTFCLAD | 0 | 318 | 1 | 0.9603 | 0 |
|  |  |  |  |  |  |  | 231 | ADTCLLL | 0 | 322 | 1 | 1.0550 | 1 |
|  |  |  |  |  |  |  | 324 | NGKCREF | 0 | 290 | 0 | 0.9513 | 0 |
|  |  |  |  |  |  |  | 344 | DKDCPIK | 0 | 302 | 1 | 1.2703 | 1 |
|  |  |  |  |  |  |  | 414 | VNMCVAT | 0 | 287 | 0 | 1.0570 | 1 |
|  |  |  |  |  |  |  | 426 | AYLCYRF | 0 | 301 | 1 | 0.9210 | 0 |
| **40** | Na.K ATPase | Sus scrofa | P05024 | [24] [25] | 23 | 13 | 91 | VKFCRQL | 0 | 301 | 1 | 0.9285 | 0 |
|  |  |  |  |  |  |  | 109 | AILCFLA | 0 | 328 | 1 | 0.9615 | 0 |
|  |  |  |  |  |  |  | **143** | **ITGCFSY** | 1 | 308 | 1 | 1.0653 | 1 |
|  |  |  |  |  |  |  | **209** | **ANGCKVD** | 1 | 306 | 1 | 1.0935 | 1 |
|  |  |  |  |  |  |  | **247** | **STNCVEG** | 1 | 313 | 1 | 1.1668 | 1 |
|  |  |  |  |  |  |  | **341** | **VTVCLTL** | 1 | 329 | 1 | 1.0230 | 1 |
|  |  |  |  |  |  |  | **354** | **RKNCLVK** | 1 | 307 | 1 | 1.0628 | 1 |
|  |  |  |  |  |  |  | **372** | **STICSDK** | 1 | 325 | 1 | 0.9940 | 0 |
|  |  |  |  |  |  |  | 426 | AGLCNRA | 0 | 319 | 1 | 1.0160 | 1 |
|  |  |  |  |  |  |  | **457** | **LLKCIEL** | 1 | 312 | 1 | 0.9903 | 0 |
|  |  |  |  |  |  |  | **461** | **IELCCGS** | 1 | 310 | 1 | 0.8573 | 0 |
|  |  |  |  |  |  |  | **462** | **ELCCGSV** | 1 | 305 | 1 | 0.8645 | 0 |
|  |  |  |  |  |  |  | **516** | **LDRCSSI** | 1 | 293 | 0 | 0.9093 | 0 |
|  |  |  |  |  |  |  | **554** | **LGFCHLF** | 1 | 292 | 0 | 0.8533 | 0 |
|  |  |  |  |  |  |  | 582 | DNLCFVG | 0 | 303 | 1 | 1.0080 | 1 |
|  |  |  |  |  |  |  | 604 | VGKCRSA | 0 | 307 | 1 | 0.8815 | 0 |
|  |  |  |  |  |  |  | **661** | **AKACVVH** | 1 | 318 | 1 | 1.1748 | 1 |
|  |  |  |  |  |  |  | **703** | **VEGCQRQ** | 1 | 292 | 0 | 1.0465 | 1 |
|  |  |  |  |  |  |  | 807 | TILCIDL | 0 | 309 | 1 | 0.9915 | 0 |
|  |  |  |  |  |  |  | 916 | EFTCHTP | 0 | 291 | 0 | 0.9518 | 0 |
|  |  |  |  |  |  |  | 935 | LVICKTR | 0 | 307 | 1 | 1.0595 | 1 |
|  |  |  |  |  |  |  | 969 | LSYCPGM | 0 | 289 | 0 | 1.1183 | 1 |
|  |  |  |  |  |  |  | 988 | WWFCAFP | 0 | 267 | 0 | 0.8835 | 0 |
| **41** | cAMP-dependent protein kinase catalytic subunit alpha | Homo sapiens | P17612 | [26] | 2 | 2 | **200** | **WTLCGTP** | 1 | 329 | 1 | 1.0873 | 1 |
|  |  |  |  |  |  |  | **344** | **NEKCGKE** | 1 | 315 | 1 | 1.0195 | 1 |
| **42** | HIV-1 protease | Human immunodeficiency virus 1 | P04585 | [27] | 20 | 2 | 57 | SEGCRQI | 0 | 297 | 1 | 1.0465 | 1 |
|  |  |  |  |  |  |  | 87 | TLYCVHQ | 0 | 284 | 0 | 1.0073 | 1 |
|  |  |  |  |  |  |  | 330 | NPDCKTI | 0 | 298 | 1 | 1.1508 | 1 |
|  |  |  |  |  |  |  | 350 | MTACQGV | 0 | 301 | 1 | 1.0388 | 1 |
|  |  |  |  |  |  |  | 392 | IVKCFNC | 0 | 281 | 0 | 0.9090 | 0 |
|  |  |  |  |  |  |  | 395 | CFNCGKE | 0 | 296 | 1 | 1.0650 | 1 |
|  |  |  |  |  |  |  | 405 | ARNCRAP | 0 | 296 | 1 | 0.9253 | 0 |
|  |  |  |  |  |  |  | 413 | KKGCWKC | 0 | 290 | 0 | 1.0505 | 1 |
|  |  |  |  |  |  |  | 416 | CWKCGKE | 0 | 296 | 1 | 0.8920 | 0 |
|  |  |  |  |  |  |  | 426 | MKDCTER | 0 | 296 | 1 | 1.2135 | 1 |
|  |  |  |  |  |  |  | **555** | **IEICGHK** | 1 | 313 | 1 | 1.0918 | 1 |
|  |  |  |  |  |  |  | **583** | **QIGCTLN** | 1 | 308 | 1 | 1.1095 | 1 |
|  |  |  |  |  |  |  | 625 | VEICTEM | 0 | 302 | 1 | 1.1493 | 1 |
|  |  |  |  |  |  |  | 867 | RQLCKLL | 0 | 320 | 1 | 0.9118 | 0 |
|  |  |  |  |  |  |  | 1187 | VASCDKC | 0 | 291 | 0 | 1.0868 | 1 |
|  |  |  |  |  |  |  | 1190 | CDKCQLK | 0 | 288 | 0 | 0.9665 | 0 |
|  |  |  |  |  |  |  | 1203 | QVDCSPG | 0 | 307 | 1 | 1.1165 | 1 |
|  |  |  |  |  |  |  | 1212 | QLDCTHL | 0 | 303 | 1 | 1.0953 | 1 |
|  |  |  |  |  |  |  | 1277 | RAACWWA | 0 | 296 | 1 | 1.0515 | 1 |
|  |  |  |  |  |  |  | 1427 | GDDCVAS | 0 | 311 | 1 | 1.2638 | 1 |
| **43** | Nucleophosmin 1 | Homo sapiens | P06748 | [28] | 3 | 1 | 21 | LFGCELK | 0 | 318 | 1 | 1.1038 | 1 |
|  |  |  |  |  |  |  | 104 | RLKCGSG | 0 | 318 | 1 | 0.9648 | 0 |
|  |  |  |  |  |  |  | **275** | **VKNCFRM** | 1 | 291 | 0 | 1.0733 | 1 |
| **44** | Myosin 2 | Dictyostelium discoideum | P08799 | [29] | 9 | 1 | 49 | SYECGEI | 0 | 301 | 1 | 1.1208 | 1 |
|  |  |  |  |  |  |  | 312 | QSGCVDI | 0 | 292 | 0 | 1.0978 | 1 |
|  |  |  |  |  |  |  | **394** | **KALCEPR** | 1 | 314 | 1 | 1.1685 | 1 |
|  |  |  |  |  |  |  | 442 | NVLCQER | 0 | 298 | 1 | 0.9643 | 0 |
|  |  |  |  |  |  |  | 470 | EQLCINY | 0 | 285 | 0 | 0.9133 | 0 |
|  |  |  |  |  |  |  | 599 | LELCFKD | 0 | 313 | 1 | 0.9708 | 0 |
|  |  |  |  |  |  |  | 655 | FVRCIIP | 0 | 277 | 0 | 0.9448 | 0 |
|  |  |  |  |  |  |  | 678 | QLRCNGV | 0 | 283 | 0 | 0.9050 | 0 |
|  |  |  |  |  |  |  | 1474 | DERCNSA | 0 | 293 | 0 | 0.9245 | 0 |
| **45** | 3,4-dihydroxy-2-butanone 4-phosphate synthase | Saccharomyces cerevisiae | Q99258; D6VTB0 | [30] [31] [32] | 6 | 3 | **34** | **DLICAAE** | 1 | 317 | 1 | 1.0505 | 1 |
|  |  |  |  |  |  |  | **56** | **GYVCAPM** | 1 | 296 | 1 | 1.0433 | 1 |
|  |  |  |  |  |  |  | 113 | SMTCRAL | 0 | 301 | 1 | 0.9458 | 0 |
|  |  |  |  |  |  |  | **133** | **GHICPLR** | 1 | 308 | 1 | 1.1120 | 1 |
|  |  |  |  |  |  |  | 156 | VDLCKLS | 0 | 324 | 1 | 1.0255 | 1 |
|  |  |  |  |  |  |  | 185 | LNDCQAF | 0 | 283 | 0 | 1.0628 | 1 |
| **46** | ATP-sensitive inward rectifier potassium channel 8 | Rattus norvegicus | Q63664; Q9JM49; Q9JM50 | [33] [34] [7]  [35] | 9 | 3 | **43** | **SGACNLA** | 1 | 326 | 1 | 1.0785 | 1 |
|  |  |  |  |  |  |  | 82 | SFLCSWL | 0 | 312 | 1 | 0.8333 | 0 |
|  |  |  |  |  |  |  | **120** | **SAVCVTN** | 1 | 307 | 1 | 1.1553 | 1 |
|  |  |  |  |  |  |  | 152 | TEECPLA | 0 | 313 | 1 | 1.1795 | 1 |
|  |  |  |  |  |  |  | **176** | **MLGCIFM** | 1 | 284 | 0 | 1.0560 | 1 |
|  |  |  |  |  |  |  | 207 | GKLCFMF | 0 | 301 | 1 | 0.9420 | 0 |
|  |  |  |  |  |  |  | 268 | LIICHVI | 0 | 284 | 0 | 1.0018 | 1 |
|  |  |  |  |  |  |  | 353 | APRCSAR | 0 | 317 | 1 | 0.8833 | 0 |
|  |  |  |  |  |  |  | 420 | GNQCPSE | 0 | 299 | 1 | 1.1035 | 1 |
| **47** | Putative nucleic acid-binding protein | Chlamydomonas incerta | Q1WLV5 | [36] | 2 | 1 | 284 | PWSCQWQ | 0 | 257 | 0 | 0.7743 | 0 |
|  |  |  |  |  |  |  | **226** | **AMAC---** | 1 | 284 | 0 | 1.0725 | 1 |
| **48** | NADH-ubiquinone oxidoreductase 75 kDa subunit, mitochondrial | Bos taurus | P15690; Q0VCP7 | [37] | 18 | 1 | 20 | SKGCVRT | 0 | 309 | 1 | 1.1570 | 1 |
|  |  |  |  |  |  |  | 53 | LQACEKV | 0 | 301 | 1 | 1.1015 | 1 |
|  |  |  |  |  |  |  | 64 | PRFCYHE | 0 | 282 | 0 | 0.8283 | 0 |
|  |  |  |  |  |  |  | 75 | AGNCRMC | 0 | 281 | 0 | 0.9485 | 0 |
|  |  |  |  |  |  |  | 78 | CRMCLVE | 0 | 284 | 0 | 0.8993 | 0 |
|  |  |  |  |  |  |  | 92 | VAACAMP | 0 | 307 | 1 | 1.1245 | 1 |
|  |  |  |  |  |  |  | 128 | PLDCPIC | 0 | 285 | 0 | 1.2238 | 1 |
|  |  |  |  |  |  |  | 131 | CPICDQG | 0 | 295 | 1 | 1.0980 | 1 |
|  |  |  |  |  |  |  | 137 | GGECDLQ | 0 | 307 | 1 | 1.0665 | 1 |
|  |  |  |  |  |  |  | 176 | MTRCIQC | 0 | 280 | 0 | 0.9310 | 0 |
|  |  |  |  |  |  |  | 179 | CIQCTRC | 0 | 266 | 0 | 1.0755 | 1 |
|  |  |  |  |  |  |  | 182 | CTRCIRF | 0 | 282 | 0 | 0.9433 | 0 |
|  |  |  |  |  |  |  | 226 | IDICPVG | 0 | 300 | 1 | 1.2785 | 1 |
|  |  |  |  |  |  |  | **367** | **DTLCTEE** | 1 | 332 | 1 | 1.0850 | 1 |
|  |  |  |  |  |  |  | 554 | DGGCITR | 0 | 305 | 1 | 1.1175 | 1 |
|  |  |  |  |  |  |  | 564 | PKDCFIV | 0 | 286 | 0 | 1.1478 | 1 |
|  |  |  |  |  |  |  | 710 | MAKCVKA | 0 | 297 | 1 | 1.0580 | 1 |
|  |  |  |  |  |  |  | 727 | PSIC--- | 0 | 266 | 0 | 1.0380 | 1 |
| **49** | NADH dehydrogenase [ubiquinone] flavoprotein 1, mitochondrial | Bos taurus | P25708; Q148I2 | [37] | 12 | 1 | 125 | PGTCKDR | 0 | 296 | 1 | 0.9863 | 0 |
|  |  |  |  |  |  |  | 142 | VEGCLVG | 0 | 315 | 1 | 1.0940 | 1 |
|  |  |  |  |  |  |  | **187** | **KNACGSG** | 1 | 323 | 1 | 1.1133 | 1 |
|  |  |  |  |  |  |  | 206 | AYICGEE | 0 | 325 | 1 | 1.0963 | 1 |
|  |  |  |  |  |  |  | 238 | VFGCPTT | 0 | 306 | 1 | 1.2050 | 1 |
|  |  |  |  |  |  |  | 255 | PTICRRG | 0 | 307 | 1 | 1.0210 | 1 |
|  |  |  |  |  |  |  | 286 | NNPCTVE | 0 | 289 | 0 | 1.1225 | 1 |
|  |  |  |  |  |  |  | 332 | KSVCETV | 0 | 296 | 1 | 1.0908 | 1 |
|  |  |  |  |  |  |  | 379 | HESCGQC | 0 | 292 | 0 | 1.0930 | 1 |
|  |  |  |  |  |  |  | 382 | CGQCTPC | 0 | 276 | 0 | 1.0998 | 1 |
|  |  |  |  |  |  |  | 385 | CTPCREG | 0 | 296 | 1 | 1.0558 | 1 |
|  |  |  |  |  |  |  | 425 | HTICALG | 0 | 317 | 1 | 1.0660 | 1 |
| **50** | Estrogen sulfotransferase | Homo sapiens | P49888; Q8N6X5 | [38] | 4 | 1 | 69 | VEKCKED | 0 | 306 | 1 | 1.0095 | 1 |
|  |  |  |  |  |  |  | **83** | **FLECRKE** | 1 | 299 | 1 | 0.9605 | 0 |
|  |  |  |  |  |  |  | 122 | EKDCKII | 0 | 293 | 0 | 1.1343 | 1 |
|  |  |  |  |  |  |  | 128 | IYLCRNA | 0 | 301 | 1 | 0.8258 | 0 |
| **51** | Ras-specific guanine nucleotide-releasing factor 1 | Mus musculus | P27671 | [39] | 21 | 1 | 73 | GSICKRA | 0 | 306 | 1 | 1.0150 | 1 |
|  |  |  |  |  |  |  | **118** | **AKDCDEW** | 1 | 304 | 1 | 1.2000 | 1 |
|  |  |  |  |  |  |  | 222 | GWLCRRK | 0 | 304 | 1 | 0.8323 | 0 |
|  |  |  |  |  |  |  | 354 | LAHCKQN | 0 | 295 | 1 | 0.9625 | 0 |
|  |  |  |  |  |  |  | 373 | KPDCEER | 0 | 307 | 1 | 1.2533 | 1 |
|  |  |  |  |  |  |  | 451 | TEGCEIL | 0 | 305 | 1 | 1.1968 | 1 |
|  |  |  |  |  |  |  | 496 | ERQCFLF | 0 | 288 | 0 | 0.8925 | 0 |
|  |  |  |  |  |  |  | 506 | LIICTRG | 0 | 301 | 1 | 1.1090 | 1 |
|  |  |  |  |  |  |  | 527 | LIDCTLL | 0 | 316 | 1 | 1.1428 | 1 |
|  |  |  |  |  |  |  | 586 | IIQCVDN | 0 | 287 | 0 | 1.0595 | 1 |
|  |  |  |  |  |  |  | 592 | NIRCNGL | 0 | 288 | 0 | 0.9485 | 0 |
|  |  |  |  |  |  |  | 620 | SLYCDDV | 0 | 291 | 0 | 1.0000 | 0 |
|  |  |  |  |  |  |  | 634 | MNSCKVL | 0 | 291 | 0 | 1.0310 | 1 |
|  |  |  |  |  |  |  | 764 | SLGCSSD | 0 | 318 | 1 | 0.9670 | 0 |
|  |  |  |  |  |  |  | 790 | GKLCMAS | 0 | 326 | 1 | 0.9845 | 0 |
|  |  |  |  |  |  |  | 878 | MTTCRDL | 0 | 302 | 1 | 0.9785 | 0 |
|  |  |  |  |  |  |  | 970 | RVICFLE | 0 | 316 | 1 | 1.0173 | 1 |
|  |  |  |  |  |  |  | 1109 | ADICRCL | 0 | 299 | 1 | 0.8755 | 0 |
|  |  |  |  |  |  |  | 1111 | ICRCLHN | 0 | 285 | 0 | 0.6480 | 0 |
|  |  |  |  |  |  |  | 1171 | LRNCDPP | 0 | 291 | 0 | 1.0495 | 1 |
|  |  |  |  |  |  |  | 1175 | DPPCVPY | 0 | 292 | 0 | 1.1773 | 1 |
| **52** | Paired box protein Pax-8 | Rattus norvegicus | P51974; Q66HM8 | [40] [41] [42] | 6 | 2 | **45** | **VRPCDIS** | 1 | 286 | 0 | 1.0153 | 1 |
|  |  |  |  |  |  |  | **57** | **SHGCVSK** | 1 | 309 | 1 | 1.0815 | 1 |
|  |  |  |  |  |  |  | 117 | EGVCDND | 0 | 298 | 1 | 0.9763 | 0 |
|  |  |  |  |  |  |  | 147 | MDSCVAT | 0 | 288 | 0 | 1.1680 | 1 |
|  |  |  |  |  |  |  | 207 | QDSCRLS | 0 | 301 | 1 | 1.0298 | 1 |
|  |  |  |  |  |  |  | 239 | PLECPFE | 0 | 301 | 1 | 1.1478 | 1 |
| **53** | Protease/reverse transcriptase | Human immunodeficiency virus 1 | Q9QIL5 | [43] [44] | 4 | 1 | 67 | IEICGHK | 0 | 313 | 1 | 1.0918 | 1 |
|  |  |  |  |  |  |  | **95** | **QIGCTLN** | 1 | 308 | 1 | 1.1095 | 1 |
|  |  |  |  |  |  |  | 137 | VEICAEL | 0 | 316 | 1 | 1.1353 | 1 |
|  |  |  |  |  |  |  | 280 | IVICQYM | 0 | 278 | 0 | 0.9550 | 0 |
| **54** | Glutathione S-transferase | Rattus norvegicus | Q5BK56 | [45] | 3 | 1 | 87 | HNLCGET | 0 | 308 | 1 | 1.0848 | 1 |
|  |  |  |  |  |  |  | **115** | **ARVCYSP** | 1 | 296 | 1 | 0.8720 | 0 |
|  |  |  |  |  |  |  | 174 | EPKCLDA | 0 | 317 | 1 | 0.9438 | 0 |
| **55** | GA repeat binding protein, alpha (Predicted) | Rattus norvegicus | D4ACQ9 | [46] [47] | 9 | 3 | 22 | KPECTEE | 0 | 312 | 1 | 1.1803 | 1 |
|  |  |  |  |  |  |  | 37 | PAECVSQ | 0 | 291 | 0 | 1.1568 | 1 |
|  |  |  |  |  |  |  | 61 | RLQCSLD | 0 | 309 | 1 | 0.8993 | 0 |
|  |  |  |  |  |  |  | 69 | HEICLQD | 0 | 297 | 1 | 1.0270 | 1 |
|  |  |  |  |  |  |  | 223 | RELCSLN | 0 | 329 | 1 | 0.9218 | 0 |
|  |  |  |  |  |  |  | 338 | ARDCISW | 0 | 287 | 0 | 1.0125 | 1 |
|  |  |  |  |  |  |  | **388** | **DMICKVQ** | 1 | 286 | 0 | 1.0153 | 1 |
|  |  |  |  |  |  |  | **401** | **KFVCDLK** | 1 | 310 | 1 | 0.9913 | 0 |
|  |  |  |  |  |  |  | **421** | **VTECEQK** | 1 | 312 | 1 | 1.1560 | 1 |
| **56** | Carbonic anhydrase 3 | Rattus norvegicus | P14141; O54961; Q9QV77 | [13] [48] [49] [50] | 5 | 2 | 66 | GKTCRVV | 0 | 295 | 1 | 1.0180 | 1 |
|  |  |  |  |  |  |  | **182** | **DPSCLFP** | 1 | 300 | 1 | 1.0038 | 1 |
|  |  |  |  |  |  |  | **187** | **FPACRDY** | 1 | 302 | 1 | 1.0398 | 1 |
|  |  |  |  |  |  |  | 202 | TPPCEEC | 0 | 290 | 0 | 1.1865 | 1 |
|  |  |  |  |  |  |  | 205 | CEECIVW | 0 | 270 | 0 | 1.1303 | 1 |
| **57** | Guanidinoacetate N-methyltransferase | Rattus norvegicus | P10868 | [51] | 5 | 1 | **16** | **GEDCGPA** | 1 | 323 | 1 | 1.2430 | 1 |
|  |  |  |  |  |  |  | 91 | IIECNDG | 0 | 285 | 0 | 1.0963 | 1 |
|  |  |  |  |  |  |  | 169 | LTYCNLT | 0 | 302 | 1 | 1.0403 | 1 |
|  |  |  |  |  |  |  | 208 | ENICTEV | 0 | 296 | 1 | 1.1333 | 1 |
|  |  |  |  |  |  |  | 220 | PADCRYY | 0 | 279 | 0 | 1.0960 | 1 |
| **58** | NF-kappa-B inhibitor alpha | Homo sapiens | P25963; B2R8L6 | [5]  [52] | 8 | 1 | 135 | GAGCDPE | 0 | 319 | 1 | 1.2260 | 1 |
|  |  |  |  |  |  |  | 152 | HLACEQG | 0 | 310 | 1 | 1.1168 | 1 |
|  |  |  |  |  |  |  | 156 | EQGCLAS | 0 | 315 | 1 | 1.0300 | 1 |
|  |  |  |  |  |  |  | 167 | TQSCTTP | 0 | 290 | 0 | 1.0665 | 1 |
|  |  |  |  |  |  |  | **186** | **GHTCLHL** | 1 | 302 | 1 | 0.9388 | 0 |
|  |  |  |  |  |  |  | 215 | QEPCNGR | 0 | 283 | 0 | 1.1020 | 1 |
|  |  |  |  |  |  |  | 239 | LLKCGAD | 0 | 322 | 1 | 1.0015 | 1 |
|  |  |  |  |  |  |  | 308 | YDDCVFG | 0 | 292 | 0 | 1.2490 | 1 |
| **59** | Mitogen-activated protein kinase kinase kinase 1 | Rattus norvegicus | Q62925 | [1]  [53] | 36 | 1 | 86 | SPPCPST | 0 | 301 | 1 | 1.1623 | 1 |
|  |  |  |  |  |  |  | 120 | ASRCGSH | 0 | 303 | 1 | 0.9115 | 0 |
|  |  |  |  |  |  |  | 183 | KATCMPA | 0 | 301 | 1 | 1.1218 | 1 |
|  |  |  |  |  |  |  | 337 | PQNCSCG | 0 | 279 | 0 | 0.7698 | 0 |
|  |  |  |  |  |  |  | 339 | NCSCGRG | 0 | 297 | 1 | 0.8963 | 0 |
|  |  |  |  |  |  |  | 345 | GTFCIHL | 0 | 306 | 1 | 0.9695 | 0 |
|  |  |  |  |  |  |  | 408 | MSNCHTL | 0 | 284 | 0 | 0.9820 | 0 |
|  |  |  |  |  |  |  | 433 | EQMCPIC | 0 | 266 | 0 | 1.1095 | 1 |
|  |  |  |  |  |  |  | 436 | CPICLLG | 0 | 311 | 1 | 1.0100 | 1 |
|  |  |  |  |  |  |  | 449 | LTVCEDG | 0 | 314 | 1 | 1.0843 | 1 |
|  |  |  |  |  |  |  | 453 | EDGCRNK | 0 | 299 | 1 | 1.0613 | 1 |
|  |  |  |  |  |  |  | 461 | HHHCMSI | 0 | 268 | 0 | 0.8488 | 0 |
|  |  |  |  |  |  |  | 469 | AEECRRN | 0 | 300 | 1 | 1.0470 | 1 |
|  |  |  |  |  |  |  | 478 | PLICPLC | 0 | 304 | 1 | 1.1033 | 1 |
|  |  |  |  |  |  |  | 481 | CPLCRSK | 0 | 308 | 1 | 0.9285 | 0 |
|  |  |  |  |  |  |  | 562 | LVGCLFS | 0 | 313 | 1 | 1.0445 | 1 |
|  |  |  |  |  |  |  | 627 | EAFCSVL | 0 | 310 | 1 | 1.0063 | 1 |
|  |  |  |  |  |  |  | 634 | SIVCADP | 0 | 297 | 1 | 1.0355 | 1 |
|  |  |  |  |  |  |  | 658 | YTPCHSL | 0 | 306 | 1 | 0.9550 | 0 |
|  |  |  |  |  |  |  | 682 | LVKCADA | 0 | 308 | 1 | 0.9860 | 0 |
|  |  |  |  |  |  |  | 701 | LELCKGQ | 0 | 313 | 1 | 0.9865 | 0 |
|  |  |  |  |  |  |  | 731 | VLSCILG | 0 | 310 | 1 | 0.9735 | 0 |
|  |  |  |  |  |  |  | 750 | GRLCLID | 0 | 305 | 1 | 0.8853 | 0 |
|  |  |  |  |  |  |  | 948 | HSQCLNS | 0 | 283 | 0 | 0.8993 | 0 |
|  |  |  |  |  |  |  | 968 | SAPCSSA | 0 | 309 | 1 | 1.0293 | 1 |
|  |  |  |  |  |  |  | 993 | FVPCKIP | 0 | 282 | 0 | 1.0575 | 1 |
|  |  |  |  |  |  |  | 1014 | QRTCSEN | 0 | 297 | 1 | 0.9280 | 0 |
|  |  |  |  |  |  |  | 1072 | ASQCDDS | 0 | 299 | 1 | 1.0058 | 1 |
|  |  |  |  |  |  |  | 1101 | EDKCRLD | 0 | 303 | 1 | 0.9788 | 0 |
|  |  |  |  |  |  |  | 1158 | NQKCKEK | 0 | 294 | 0 | 0.9650 | 0 |
|  |  |  |  |  |  |  | **1238** | **FSSCYQA** | 1 | 290 | 0 | 0.9728 | 0 |
|  |  |  |  |  |  |  | 1295 | GATCEKS | 0 | 306 | 1 | 1.1368 | 1 |
|  |  |  |  |  |  |  | 1411 | GRSCDVW | 0 | 285 | 0 | 0.9895 | 0 |
|  |  |  |  |  |  |  | 1418 | SVGCAII | 0 | 294 | 0 | 1.1395 | 1 |
|  |  |  |  |  |  |  | 1425 | EMACAKP | 0 | 309 | 1 | 1.0460 | 1 |
|  |  |  |  |  |  |  | 1469 | ALRCLEL | 0 | 322 | 1 | 0.8770 | 0 |
| **60** | Triosephosphate isomerase | Plasmodium falciparum | Q07412 | [54] | 4 | 1 | 13 | NWKCNGT | 0 | 273 | 0 | 0.8945 | 0 |
|  |  |  |  |  |  |  | 126 | AVVCFGE | 0 | 313 | 1 | 1.0128 | 1 |
|  |  |  |  |  |  |  | 196 | KDTCGEK | 0 | 313 | 1 | 1.2150 | 1 |
|  |  |  |  |  |  |  | **217** | **TENCSSL** | 1 | 308 | 1 | 0.9928 | 0 |
| **61** | Succinate dehydrogenase [ubiquinone] flavoprotein subunit, mitochondrial | Homo sapiens | P31040; A8K5J6; B4DJ60; E9PBJ5; Q16395; Q59GW8; Q8IW48; Q9UMY5 | [17] | 18 | 1 | **89** | **NTACVTK** | 1 | 331 | 1 | 1.1800 | 1 |
|  |  |  |  |  |  |  | 189 | AHRCCCV | 0 | 273 | 0 | 0.5238 | 0 |
|  |  |  |  |  |  |  | 190 | HRCCCVA | 0 | 273 | 0 | 0.6600 | 0 |
|  |  |  |  |  |  |  | 191 | RCCCVAD | 0 | 288 | 0 | 0.7385 | 0 |
|  |  |  |  |  |  |  | 231 | NGECRGV | 0 | 287 | 0 | 1.0155 | 1 |
|  |  |  |  |  |  |  | 238 | IALCIED | 0 | 309 | 1 | 1.1005 | 1 |
|  |  |  |  |  |  |  | 266 | YFSCTSA | 0 | 300 | 1 | 1.0113 | 1 |
|  |  |  |  |  |  |  | 287 | GLPCQDL | 0 | 297 | 1 | 0.9365 | 0 |
|  |  |  |  |  |  |  | 305 | GAGCLIT | 0 | 302 | 1 | 1.1348 | 1 |
|  |  |  |  |  |  |  | 311 | TEGCRGE | 0 | 310 | 1 | 1.0533 | 1 |
|  |  |  |  |  |  |  | 357 | GRGCGPE | 0 | 324 | 1 | 1.1033 | 1 |
|  |  |  |  |  |  |  | 438 | LYACGEA | 0 | 331 | 1 | 1.1318 | 1 |
|  |  |  |  |  |  |  | 443 | EAACASV | 0 | 315 | 1 | 1.1450 | 1 |
|  |  |  |  |  |  |  | 467 | GRACALS | 0 | 327 | 1 | 0.9965 | 0 |
|  |  |  |  |  |  |  | 475 | EESCRPG | 0 | 304 | 1 | 1.0383 | 1 |
|  |  |  |  |  |  |  | 536 | QEGCGKI | 0 | 308 | 1 | 1.1330 | 1 |
|  |  |  |  |  |  |  | 574 | LMLCALQ | 0 | 311 | 1 | 0.9468 | 0 |
|  |  |  |  |  |  |  | 654 | EADCATV | 0 | 304 | 1 | 1.2368 | 1 |
| **62** | Isocitrate dehydrogenase [NADP], mitochondrial | Mus musculus | P54071; Q8C2R9; Q9EQK1 | [55] [56] [57] | 8 | 3 | 12 | SSLCRAS | 0 | 318 | 1 | 0.9190 | 0 |
|  |  |  |  |  |  |  | **113** | **AVKCATI** | 1 | 306 | 1 | 1.0308 | 1 |
|  |  |  |  |  |  |  | **154** | **PIICKNI** | 1 | 282 | 0 | 0.9798 | 0 |
|  |  |  |  |  |  |  | 235 | AHSCFQY | 0 | 288 | 0 | 0.9868 | 0 |
|  |  |  |  |  |  |  | **308** | **VWACKNY** | 1 | 290 | 0 | 0.8960 | 0 |
|  |  |  |  |  |  |  | 336 | VLVCPDG | 0 | 308 | 1 | 1.0795 | 1 |
|  |  |  |  |  |  |  | 402 | EKVCVQT | 0 | 302 | 1 | 1.0738 | 1 |
|  |  |  |  |  |  |  | 418 | LAGCIHG | 0 | 301 | 1 | 1.1278 | 1 |
| **63** | Phosphoadenosine phosphosulfate reductase | Escherichia coli | P17854; Q2MA67 | [58] | 1 | 1 | **239** | **KRECGLH** | 1 | 304 | 1 | 1.0138 | 1 |
| **64** | Cystic fibrosis transmembrane conductance regulator | Homo sapiens | P13569; Q20BG8; Q20BH2; Q2I0A1; Q2I102 | [59] | 18 | 1 | 76 | LRRCFFW | 0 | 273 | 0 | 0.8035 | 0 |
|  |  |  |  |  |  |  | 128 | IGLCLLF | 0 | 321 | 1 | 0.9148 | 0 |
|  |  |  |  |  |  |  | 225 | SAFCGLG | 0 | 318 | 1 | 1.0633 | 1 |
|  |  |  |  |  |  |  | 276 | KAYCWEE | 0 | 291 | 0 | 1.1030 | 1 |
|  |  |  |  |  |  |  | 343 | ISFCIVL | 0 | 291 | 0 | 0.9895 | 0 |
|  |  |  |  |  |  |  | 491 | ISFCSQF | 0 | 285 | 0 | 0.8918 | 0 |
|  |  |  |  |  |  |  | 524 | IKACQLE | 0 | 323 | 1 | 1.0048 | 1 |
|  |  |  |  |  |  |  | 590 | FESCVCK | 0 | 294 | 0 | 0.8835 | 0 |
|  |  |  |  |  |  |  | 592 | SCVCKLM | 0 | 297 | 1 | 0.7753 | 0 |
|  |  |  |  |  |  |  | 647 | LMGCDSF | 0 | 293 | 0 | 1.0443 | 1 |
|  |  |  |  |  |  |  | 832 | LKECFFD | 0 | 294 | 0 | 1.0718 | 1 |
|  |  |  |  |  |  |  | 866 | LIWCLVI | 0 | 283 | 0 | 0.9198 | 0 |
|  |  |  |  |  |  |  | **1344** | **DGGCVLS** | 1 | 323 | 1 | 1.1005 | 1 |
|  |  |  |  |  |  |  | 1355 | QLMCLAR | 0 | 298 | 1 | 0.9345 | 0 |
|  |  |  |  |  |  |  | 1395 | FADCTVI | 0 | 289 | 0 | 1.2493 | 1 |
|  |  |  |  |  |  |  | 1400 | VILCEHR | 0 | 303 | 1 | 1.0345 | 1 |
|  |  |  |  |  |  |  | 1410 | MLECQQF | 0 | 269 | 0 | 0.9708 | 0 |
|  |  |  |  |  |  |  | 1458 | SSKCKSK | 0 | 306 | 1 | 0.9000 | 0 |
| **65** | Selenocysteine insertion sequence-binding protein 2 | Homo sapiens | Q96T21; F8W892; Q5HYY1; Q7L1Z0; Q8IYC0; Q9H0A1 | [60] | 12 | 2 | 41 | SEACVFP | 0 | 312 | 1 | 1.1458 | 1 |
|  |  |  |  |  |  |  | 118 | QPSCYRG | 0 | 296 | 1 | 1.0313 | 1 |
|  |  |  |  |  |  |  | 133 | ENTCPLP | 0 | 305 | 1 | 1.1213 | 1 |
|  |  |  |  |  |  |  | 291 | SPSCTRE | 0 | 312 | 1 | 1.0975 | 1 |
|  |  |  |  |  |  |  | 489 | SKECASG | 0 | 307 | 1 | 1.0848 | 1 |
|  |  |  |  |  |  |  | 633 | RDYCSQM | 0 | 286 | 0 | 1.0500 | 1 |
|  |  |  |  |  |  |  | **644** | **VDACVTD** | 1 | 315 | 1 | 1.2508 | 1 |
|  |  |  |  |  |  |  | 691 | KLKCVII | 0 | 290 | 0 | 1.0123 | 1 |
|  |  |  |  |  |  |  | 698 | SPNCEKI | 0 | 293 | 0 | 1.1273 | 1 |
|  |  |  |  |  |  |  | 719 | DYACEQN | 0 | 306 | 1 | 1.1013 | 1 |
|  |  |  |  |  |  |  | **803** | **GPSCPAE** | 1 | 323 | 1 | 1.1700 | 1 |
|  |  |  |  |  |  |  | 834 | YSGCTLE | 0 | 318 | 1 | 1.0780 | 1 |
| **66** | 5-methyltetrahydropteroyltriglutamate--homocysteine methyltransferase | Escherichia coli | P25665; Q2M8D4 | [61] | 7 | 1 | 323 | ASSCSLL | 0 | 326 | 1 | 0.9185 | 0 |
|  |  |  |  |  |  |  | 353 | LQKCHEL | 0 | 295 | 1 | 0.9018 | 0 |
|  |  |  |  |  |  |  | 516 | GSRCVKP | 0 | 295 | 1 | 0.9090 | 0 |
|  |  |  |  |  |  |  | 560 | TILCWSF | 0 | 288 | 0 | 0.9455 | 0 |
|  |  |  |  |  |  |  | 643 | THMCYCE | 0 | 274 | 0 | 0.7398 | 0 |
|  |  |  |  |  |  |  | **645** | **MCYCEFN** | 1 | 266 | 0 | 0.8933 | 0 |
|  |  |  |  |  |  |  | 726 | NPDCGLK | 0 | 325 | 1 | 1.1718 | 1 |
| **67** | Carbonic anhydrase 7 | Homo sapiens | P43166; Q541F0; Q86YU0 | [22] | 4 | 2 | 57 | YEACMSL | 0 | 319 | 1 | 1.0513 | 1 |
|  |  |  |  |  |  |  | 180 | QFSCFNP | 0 | 281 | 0 | 0.9333 | 0 |
|  |  |  |  |  |  |  | **185** | **NPKCLLP** | 1 | 313 | 1 | 0.9328 | 0 |
|  |  |  |  |  |  |  | **219** | **EPICISE** | 1 | 312 | 1 | 1.0560 | 1 |
| **68** | TNF receptor-associated factor 3 | Homo sapiens | Q13114; B7Z8C4; Q12990; Q13076; Q13947; Q6AZX1; Q9UNL1 | [62] | 28 | 1 | 53 | KYKCEKC | 0 | 287 | 0 | 0.9660 | 0 |
|  |  |  |  |  |  |  | 56 | CEKCHLV | 0 | 287 | 0 | 0.8785 | 0 |
|  |  |  |  |  |  |  | 61 | LVLCSPK | 0 | 328 | 1 | 0.9843 | 0 |
|  |  |  |  |  |  |  | 68 | QTECGHR | 0 | 308 | 1 | 1.1148 | 1 |
|  |  |  |  |  |  |  | 73 | HRFCESC | 0 | 271 | 0 | 0.9245 | 0 |
|  |  |  |  |  |  |  | 76 | CESCMAA | 0 | 296 | 1 | 1.0263 | 1 |
|  |  |  |  |  |  |  | 88 | SPKCTAC | 0 | 303 | 1 | 1.0455 | 1 |
|  |  |  |  |  |  |  | 91 | CTACQES | 0 | 312 | 1 | 1.0763 | 1 |
|  |  |  |  |  |  |  | 105 | KDNCCKR | 0 | 279 | 0 | 0.9703 | 0 |
|  |  |  |  |  |  |  | 106 | DNCCKRE | 0 | 290 | 0 | 0.8613 | 0 |
|  |  |  |  |  |  |  | 117 | QIYCRNE | 0 | 280 | 0 | 0.9203 | 0 |
|  |  |  |  |  |  |  | 124 | SRGCAEQ | 0 | 302 | 1 | 1.0650 | 1 |
|  |  |  |  |  |  |  | 141 | KNDCHFE | 0 | 288 | 0 | 1.0293 | 1 |
|  |  |  |  |  |  |  | 148 | ELPCVRP | 0 | 300 | 1 | 1.0770 | 1 |
|  |  |  |  |  |  |  | 153 | RPDCKEK | 0 | 314 | 1 | 1.1628 | 1 |
|  |  |  |  |  |  |  | 170 | EKACKYR | 0 | 314 | 1 | 1.0450 | 1 |
|  |  |  |  |  |  |  | 177 | EATCSHC | 0 | 292 | 0 | 1.0068 | 1 |
|  |  |  |  |  |  |  | 180 | CSHCKSQ | 0 | 276 | 0 | 0.8533 | 0 |
|  |  |  |  |  |  |  | 197 | DTDCPCV | 0 | 299 | 1 | 1.0425 | 1 |
|  |  |  |  |  |  |  | 199 | DCPCVVV | 0 | 278 | 0 | 0.9383 | 0 |
|  |  |  |  |  |  |  | 204 | VVSCPHK | 0 | 304 | 1 | 1.1095 | 1 |
|  |  |  |  |  |  |  | 208 | PHKCSVQ | 0 | 291 | 0 | 0.9185 | 0 |
|  |  |  |  |  |  |  | 225 | LSECVNA | 0 | 294 | 0 | 1.0440 | 1 |
|  |  |  |  |  |  |  | 232 | PSTCSFK | 0 | 296 | 1 | 0.9470 | 0 |
|  |  |  |  |  |  |  | 239 | RYGCVFQ | 0 | 292 | 0 | 1.0793 | 1 |
|  |  |  |  |  |  |  | 296 | NQICSFE | 0 | 293 | 0 | 0.9600 | 0 |
|  |  |  |  |  |  |  | **455** | **YKMCARV** | 1 | 288 | 0 | 1.0460 | 1 |
|  |  |  |  |  |  |  | 534 | ASGCPVF | 0 | 304 | 1 | 1.2058 | 1 |
| **69** | TNF receptor-associated factor 6 | Homo sapiens | Q9Y4K3; A6NKI7; A8KAB3; D3DR16; Q8NEH5 | [62] | 30 | 1 | 6 | LLNCENS | 0 | 297 | 1 | 1.0370 | 1 |
|  |  |  |  |  |  |  | 10 | ENSCGSS | 0 | 313 | 1 | 1.0515 | 1 |
|  |  |  |  |  |  |  | 19 | ESDCCVA | 0 | 295 | 1 | 0.9738 | 0 |
|  |  |  |  |  |  |  | 20 | SDCCVAM | 0 | 291 | 0 | 1.0168 | 1 |
|  |  |  |  |  |  |  | 27 | ASSCSAV | 0 | 310 | 1 | 0.9533 | 0 |
|  |  |  |  |  |  |  | 70 | KYECPIC | 0 | 277 | 0 | 1.1633 | 1 |
|  |  |  |  |  |  |  | 73 | CPICLMA | 0 | 296 | 1 | 0.9875 | 0 |
|  |  |  |  |  |  |  | 85 | QTPCGHR | 0 | 306 | 1 | 1.0930 | 1 |
|  |  |  |  |  |  |  | 90 | HRFCKAC | 0 | 277 | 0 | 0.8708 | 0 |
|  |  |  |  |  |  |  | 93 | CKACIIK | 0 | 299 | 1 | 1.1328 | 1 |
|  |  |  |  |  |  |  | 105 | GHKCPVD | 0 | 298 | 1 | 1.0890 | 1 |
|  |  |  |  |  |  |  | 134 | MVKCPNE | 0 | 294 | 0 | 1.0315 | 1 |
|  |  |  |  |  |  |  | 139 | NEGCLHK | 0 | 308 | 1 | 1.0358 | 1 |
|  |  |  |  |  |  |  | 155 | QAHCEFA | 0 | 289 | 0 | 1.0495 | 1 |
|  |  |  |  |  |  |  | 162 | LMDCPQC | 0 | 285 | 0 | 1.1900 | 1 |
|  |  |  |  |  |  |  | 165 | CPQCQRP | 0 | 272 | 0 | 0.9833 | 0 |
|  |  |  |  |  |  |  | 182 | LKDCPRR | 0 | 307 | 1 | 1.2550 | 1 |
|  |  |  |  |  |  |  | 189 | QVSCDNC | 0 | 279 | 0 | 0.9955 | 0 |
|  |  |  |  |  |  |  | 192 | CDNCAAS | 0 | 290 | 0 | 1.1818 | 1 |
|  |  |  |  |  |  |  | 208 | DQNCPLA | 0 | 298 | 1 | 1.1208 | 1 |
|  |  |  |  |  |  |  | 215 | NVICEYC | 0 | 280 | 0 | 1.0900 | 1 |
|  |  |  |  |  |  |  | 218 | CEYCNTI | 0 | 271 | 0 | 1.0975 | 1 |
|  |  |  |  |  |  |  | 235 | DLDCPTA | 0 | 314 | 1 | 1.2285 | 1 |
|  |  |  |  |  |  |  | 242 | PIPCTFS | 0 | 287 | 0 | 1.0960 | 1 |
|  |  |  |  |  |  |  | 249 | TFGCHEK | 0 | 300 | 1 | 1.0178 | 1 |
|  |  |  |  |  |  |  | 349 | AQQCNGI | 0 | 274 | 0 | 1.0213 | 1 |
|  |  |  |  |  |  |  | 366 | HLKCQEE | 0 | 295 | 1 | 0.9133 | 0 |
|  |  |  |  |  |  |  | **390** | **YKLCMRL** | 1 | 318 | 1 | 0.9855 | 0 |
|  |  |  |  |  |  |  | 403 | AQRCANY | 0 | 278 | 0 | 0.8495 | 0 |
|  |  |  |  |  |  |  | 497 | LVRCEVS | 0 | 302 | 1 | 1.0060 | 1 |
| **70** | Mitochondrial uncoupling protein 2 | Mus musculus | P70406; O88285 | [63] | 5 | 2 | **25** | **TAACIAD** | 1 | 313 | 1 | 1.1658 | 1 |
|  |  |  |  |  |  |  | 191 | IVNCAEL | 0 | 305 | 1 | 1.1303 | 1 |
|  |  |  |  |  |  |  | 216 | DLPCHFT | 0 | 278 | 0 | 0.9268 | 0 |
|  |  |  |  |  |  |  | 227 | AGFCTTV | 0 | 303 | 1 | 1.0348 | 1 |
|  |  |  |  |  |  |  | **256** | **AGHCALT** | 1 | 310 | 1 | 0.9175 | 0 |
| **71** | Mitochondrial uncoupling protein 3 | Mus musculus | P56501; O88293 | [63] | **7** | **2** | **25** | **TAACFAD** | 1 | 316 | 1 | 1.1468 | 1 |
|  |  |  |  |  |  |  | 121 | LAGCTTG | 0 | 315 | 1 | 1.2175 | 1 |
|  |  |  |  |  |  |  | 130 | AVTCAQP | 0 | 303 | 1 | 1.0580 | 1 |
|  |  |  |  |  |  |  | 190 | IVNCAEM | 0 | 292 | 0 | 1.1303 | 1 |
|  |  |  |  |  |  |  | 215 | NFPCHFV | 0 | 266 | 0 | 0.9365 | 0 |
|  |  |  |  |  |  |  | 226 | AGFCATV | 0 | 304 | 1 | 1.0208 | 1 |
|  |  |  |  |  |  |  | **255** | **PLHCMLK** | 1 | 303 | 1 | 0.8420 | 0 |
| **72** | Carbonyl reductase [NADPH] 1 | Homo sapiens | P16152; B2RBZ7; B4DFK7; Q3LHW8 | [64] | 5 | 1 | 26 | RDLCRLF | 0 | 305 | 1 | 0.9933 | 0 |
|  |  |  |  |  |  |  | 122 | RDVCTEL | 0 | 306 | 1 | 1.1838 | 1 |
|  |  |  |  |  |  |  | 150 | LKSCSPE | 0 | 321 | 1 | 1.0308 | 1 |
|  |  |  |  |  |  |  | 226 | LNACCPG | 0 | 305 | 1 | 0.9870 | 0 |
|  |  |  |  |  |  |  | **227** | **NACCPGW** | 1 | 280 | 0 | 1.0518 | 1 |
| **73** | 5'-AMP-activated protein kinase catalytic subunit alpha-1 | Mus musculus | Q5EG47 | [65] | 11 | 2 | 117 | DYICKNG | 0 | 291 | 0 | 0.9208 | 0 |
|  |  |  |  |  |  |  | 141 | VDYCHRH | 0 | 271 | 0 | 1.0335 | 1 |
|  |  |  |  |  |  |  | 185 | RTSCGSP | 0 | 322 | 1 | 1.0660 | 1 |
|  |  |  |  |  |  |  | 220 | ALLCGTL | 0 | 346 | 1 | 1.0370 | 1 |
|  |  |  |  |  |  |  | 238 | KKICDGI | 0 | 301 | 1 | 1.0930 | 1 |
|  |  |  |  |  |  |  | **308** | **KEVCEKF** | 1 | 294 | 0 | 1.0758 | 1 |
|  |  |  |  |  |  |  | **313** | **KFECSEE** | 1 | 309 | 1 | 1.0348 | 1 |
|  |  |  |  |  |  |  | 321 | VLSCLYN | 0 | 302 | 1 | 0.9213 | 0 |
|  |  |  |  |  |  |  | 425 | AEVCRAI | 0 | 303 | 1 | 0.9868 | 0 |
|  |  |  |  |  |  |  | 503 | YRSCQRS | 0 | 286 | 0 | 0.8775 | 0 |
|  |  |  |  |  |  |  | 550 | FEMCANL | 0 | 289 | 0 | 0.9845 | 0 |
| **74** | Stromal interaction molecule 1 | Homo sapiens | Q13586; E9PQJ4; Q8N382 | [66] | 5 | 1 | 4 | MDVCVRL | 0 | 293 | 0 | 1.1605 | 1 |
|  |  |  |  |  |  |  | 49 | AEFCRID | 0 | 288 | 0 | 0.9613 | 0 |
|  |  |  |  |  |  |  | **56** | **KPLCHSE** | 1 | 318 | 1 | 0.8975 | 0 |
|  |  |  |  |  |  |  | 227 | VGGCWFA | 0 | 298 | 1 | 1.0488 | 1 |
|  |  |  |  |  |  |  | 437 | EILCGFQ | 0 | 312 | 1 | 1.0448 | 1 |
| **75** | Calcium/calmodulin-dependent protein kinase type 1 | Mus musculus | Q91YS8 | [67] [68] | 10 | 1 | 50 | AIKCIAK | 0 | 310 | 1 | 1.0008 | 1 |
|  |  |  |  |  |  |  | **179** | **STACGTP** | 1 | 342 | 1 | 1.1855 | 1 |
|  |  |  |  |  |  |  | 201 | AVDCWSI | 0 | 290 | 0 | 1.0708 | 1 |
|  |  |  |  |  |  |  | 213 | ILLCGYP | 0 | 318 | 1 | 0.9770 | 0 |
|  |  |  |  |  |  |  | 267 | RFTCEQA | 0 | 296 | 1 | 1.0733 | 1 |
|  |  |  |  |  |  |  | 349 | AAGCCCR | 0 | 294 | 0 | 0.7895 | 0 |
|  |  |  |  |  |  |  | 350 | AGCCCRD | 0 | 291 | 0 | 0.7220 | 0 |
|  |  |  |  |  |  |  | 351 | GCCCRDC | 0 | 270 | 0 | 0.6113 | 0 |
|  |  |  |  |  |  |  | 354 | CRDCCVE | 0 | 281 | 0 | 0.9070 | 0 |
|  |  |  |  |  |  |  | 355 | RDCCVEP | 0 | 290 | 0 | 1.0498 | 1 |
| **76** | Isocitrate lyase | Chlamydomonas reinhardtii | A8J244 | [69] [70] | 4 | 2 | 165 | GASCVHF | 0 | 291 | 0 | 1.1040 | 1 |
|  |  |  |  |  |  |  | **178** | **AKKCGHL** | 1 | 326 | 1 | 1.0093 | 1 |
|  |  |  |  |  |  |  | **247** | **GFYCVRG** | 1 | 292 | 0 | 1.0568 | 1 |
|  |  |  |  |  |  |  | 301 | AYNCSPS | 0 | 309 | 1 | 1.0053 | 1 |
| **77** | Glutathione S-transferase P | Homo sapiens | P09211; O00460; Q15690; Q5TZY3 | [1]  [71] [72] | 4 | 2 | 15 | RGRCAAL | 0 | 314 | 1 | 0.9338 | 0 |
|  |  |  |  |  |  |  | **48** | **KASCLYG** | 1 | 301 | 1 | 1.0170 | 1 |
|  |  |  |  |  |  |  | **102** | **DLRCKYI** | 1 | 284 | 0 | 0.8205 | 0 |
|  |  |  |  |  |  |  | 170 | APGCLDA | 0 | 327 | 1 | 1.0573 | 1 |
| **78** | Superoxide dismutase [Fe] | Pseudoalteromonas haloplanktis | P84612; Q3IKP4 | [73] | 1 | 1 | **57** | **EIVCSSD** | 1 | 305 | 1 | 0.9395 | 0 |
| **79** | Gamma-crystallin S | Homo sapiens | P22914; B2RAF8 | [74] | 7 | 4 | **23** | **RYDCDCD** | 1 | 286 | 0 | 0.8898 | 0 |
|  |  |  |  |  |  |  | **25** | **DCDCDCA** | 1 | 285 | 0 | 0.7480 | 0 |
|  |  |  |  |  |  |  | **27** | **DCDCADF** | 1 | 281 | 0 | 0.9390 | 0 |
|  |  |  |  |  |  |  | 37 | LSRCNSI | 0 | 280 | 0 | 0.8848 | 0 |
|  |  |  |  |  |  |  | **83** | **LSSCRAV** | 1 | 295 | 1 | 0.9555 | 0 |
|  |  |  |  |  |  |  | 115 | TEDCPSI | 0 | 297 | 1 | 1.2225 | 1 |
|  |  |  |  |  |  |  | 130 | IHSCKVL | 0 | 300 | 1 | 1.0040 | 1 |
| **80** | DNA-binding transcriptional regulator OxyR | Haemophilus influenzae | E4QV51 | [75] | 3 | 1 | **49** | **TPTCSSS** | 1 | 312 | 1 | 0.9713 | 0 |
|  |  |  |  |  |  |  | 180 | KPGCPFC | 0 | 299 | 1 | 1.2178 | 1 |
|  |  |  |  |  |  |  | 182 | CPFCAKA | 0 | 293 | 0 | 0.9985 | 0 |
| **81** | Tyrosine 3-monooxygenase | Rattus norvegicus | P04177 | [76] | 7 | 6 | **177** | **LDKCHHL** | 1 | 293 | 0 | 0.9438 | 0 |
|  |  |  |  |  |  |  | **249** | **THACREH** | 1 | 297 | 1 | 1.0470 | 1 |
|  |  |  |  |  |  |  | **263** | **ERYCGYR** | 1 | 291 | 0 | 0.9578 | 0 |
|  |  |  |  |  |  |  | 311 | VFQCTQY | 0 | 278 | 0 | 1.0295 | 1 |
|  |  |  |  |  |  |  | **329** | **EPDCCHE** | 1 | 301 | 1 | 0.9618 | 0 |
|  |  |  |  |  |  |  | **330** | **PDCCHEL** | 1 | 287 | 0 | 0.9153 | 0 |
|  |  |  |  |  |  |  | **380** | **FGLCKQN** | 1 | 311 | 1 | 0.9538 | 0 |
| **82** | Myosin-binding protein C, cardiac-type | Mus musculus | O70468; O88997 | [56] [57] [77] | 22 | 5 | 237 | GYRCEVS | 0 | 298 | 1 | 0.9540 | 0 |
|  |  |  |  |  |  |  | 248 | FDSCNFN | 0 | 277 | 0 | 1.1320 | 1 |
|  |  |  |  |  |  |  | 341 | VEACHRP | 0 | 307 | 1 | 1.0270 | 1 |
|  |  |  |  |  |  |  | 422 | ISQCSLA | 0 | 307 | 1 | 0.9113 | 0 |
|  |  |  |  |  |  |  | **432** | **AYQCVVG** | 1 | 296 | 1 | 1.0438 | 1 |
|  |  |  |  |  |  |  | 439 | GEKCSTE | 0 | 322 | 1 | 0.9630 | 0 |
|  |  |  |  |  |  |  | 471 | EFECEVS | 0 | 299 | 1 | 1.1463 | 1 |
|  |  |  |  |  |  |  | **562** | **VFKCEVS** | 1 | 301 | 1 | 1.0445 | 1 |
|  |  |  |  |  |  |  | 619 | GFACNLS | 0 | 319 | 1 | 1.0625 | 1 |
|  |  |  |  |  |  |  | 647 | HLDCPGS | 0 | 305 | 1 | 1.2030 | 1 |
|  |  |  |  |  |  |  | **715** | **KLLCETE** | 1 | 328 | 1 | 1.0508 | 1 |
|  |  |  |  |  |  |  | 784 | EDSCTVQ | 0 | 292 | 0 | 1.1788 | 1 |
|  |  |  |  |  |  |  | 905 | VEYCQEG | 0 | 288 | 0 | 1.0150 | 1 |
|  |  |  |  |  |  |  | 909 | QEGCSEW | 0 | 302 | 1 | 1.0885 | 1 |
|  |  |  |  |  |  |  | 1120 | RTHCVVS | 0 | 310 | 1 | 1.0190 | 1 |
|  |  |  |  |  |  |  | 1197 | AILCCAV | 0 | 308 | 1 | 0.8535 | 0 |
|  |  |  |  |  |  |  | 1198 | ILCCAVR | 0 | 292 | 0 | 0.8925 | 0 |
|  |  |  |  |  |  |  | 1227 | RMFCKQG | 0 | 286 | 0 | 0.9080 | 0 |
|  |  |  |  |  |  |  | 1240 | RKPCPYD | 0 | 294 | 0 | 1.1483 | 1 |
|  |  |  |  |  |  |  | 1249 | VYVCRAT | 0 | 294 | 0 | 0.9195 | 0 |
|  |  |  |  |  |  |  | **1260** | **EAQCECR** | 1 | 284 | 0 | 0.9395 | 0 |
|  |  |  |  |  |  |  | **1262** | **QCECRLE** | 1 | 295 | 1 | 0.8023 | 0 |
| **83** | Sodium/potassium-transporting ATPase subunit beta-1 | Mus musculus | P14094 | [56] [7] | 7 | 2 | **45** | **FYGCLAG** | 1 | 311 | 1 | 1.0073 | 1 |
|  |  |  |  |  |  |  | 126 | FEDCGNV | 0 | 297 | 1 | 1.1343 | 1 |
|  |  |  |  |  |  |  | 149 | RKVCRFK | 0 | 299 | 1 | 0.9668 | 0 |
|  |  |  |  |  |  |  | 159 | LGNCSGL | 0 | 314 | 1 | 0.9990 | 0 |
|  |  |  |  |  |  |  | 175 | GKPCIII | 0 | 281 | 0 | 1.1000 | 1 |
|  |  |  |  |  |  |  | **214** | **PVQCTGK** | 1 | 295 | 1 | 1.0630 | 1 |
|  |  |  |  |  |  |  | 277 | RVECKAY | 0 | 296 | 1 | 1.0630 | 1 |
| **84** | Doa1p | Saccharomyces cerevisiae | E7NJR2 | [78] [31] | 8 | 1 | 61 | NSVCYDS | 0 | 290 | 0 | 0.9518 | 0 |
|  |  |  |  |  |  |  | 103 | GNVCSLS | 0 | 316 | 1 | 0.9338 | 0 |
|  |  |  |  |  |  |  | **196** | **FISCSND** | 1 | 290 | 0 | 0.9190 | 0 |
|  |  |  |  |  |  |  | 225 | FVYCIKL | 0 | 291 | 0 | 1.0100 | 1 |
|  |  |  |  |  |  |  | 237 | IVSCGED | 0 | 312 | 1 | 1.1220 | 1 |
|  |  |  |  |  |  |  | 269 | SVDCMSN | 0 | 299 | 1 | 1.0700 | 1 |
|  |  |  |  |  |  |  | 579 | LVNCFNN | 0 | 287 | 0 | 0.9965 | 0 |
|  |  |  |  |  |  |  | 663 | YQECEEA | 0 | 293 | 0 | 1.1573 | 1 |
| **85** | S-adenosylmethionine synthase 1 | Saccharomyces cerevisiae | P10659; D6VYI4 | [78] [31] | 5 | 1 | 21 | DKICDQV | 0 | 297 | 1 | 1.0863 | 1 |
|  |  |  |  |  |  |  | 32 | LDACLAE | 0 | 330 | 1 | 1.1430 | 1 |
|  |  |  |  |  |  |  | 43 | KVACETA | 0 | 325 | 1 | 1.1855 | 1 |
|  |  |  |  |  |  |  | **91** | **YKTCNVL** | 1 | 300 | 1 | 1.1003 | 1 |
|  |  |  |  |  |  |  | 298 | AGLCKRV | 0 | 318 | 1 | 0.9660 | 0 |
| **86** | Homocitrate dehydratase, mitochondrial | Saccharomyces cerevisiae | P39533; D6VVZ3 | [78] | 9 | 4 | **66** | **SHLCDPE** | 1 | 317 | 1 | 1.0400 | 1 |
|  |  |  |  |  |  |  | 123 | SIHCDHL | 0 | 295 | 1 | 0.9318 | 0 |
|  |  |  |  |  |  |  | **164** | **LESCAKR** | 1 | 302 | 1 | 1.0423 | 1 |
|  |  |  |  |  |  |  | 274 | TLSCTGM | 0 | 297 | 1 | 1.0338 | 1 |
|  |  |  |  |  |  |  | 281 | ATICNMG | 0 | 305 | 1 | 1.0450 | 1 |
|  |  |  |  |  |  |  | 285 | IGSCTNS | 0 | 300 | 1 | 0.9983 | 0 |
|  |  |  |  |  |  |  | **448** | **ANACGPC** | 1 | 318 | 1 | 1.1930 | 1 |
|  |  |  |  |  |  |  | **451** | **CGPCIGQ** | 1 | 278 | 0 | 1.0585 | 1 |
|  |  |  |  |  |  |  | 595 | EGKCTTD | 0 | 310 | 1 | 1.0340 | 1 |
| **87** | Carbamoyl-phosphate synthase arginine-specific small chain | Saccharomyces cerevisiae | P07258; D6W302 | [78] [31] | 8 | 1 | **11** | **ATFCIQN** | 1 | 305 | 1 | 0.9970 | 0 |
|  |  |  |  |  |  |  | 15 | AQWCQRE | 0 | 279 | 0 | 0.8360 | 0 |
|  |  |  |  |  |  |  | 191 | LIDCGVK | 0 | 314 | 1 | 1.2113 | 1 |
|  |  |  |  |  |  |  | 200 | IRCLVK | 0 | 290 | 0 | 0.8073 | 0 |
|  |  |  |  |  |  |  | 239 | PELCQAT | 0 | 302 | 1 | 0.9465 | 0 |
|  |  |  |  |  |  |  | 257 | VYDCIPI | 0 | 287 | 0 | 1.1315 | 1 |
|  |  |  |  |  |  |  | 264 | FGICLGH | 0 | 299 | 1 | 1.0078 | 1 |
|  |  |  |  |  |  |  | 300 | TGQCHIT | 0 | 272 | 0 | 0.9473 | 0 |
| **88** | 60S ribosomal protein L23-A | Saccharomyces cerevisiae | P0CX41; D3DM23; P04451 | [78] [31] | 2 | 2 | **25** | **IMNCADN** | 1 | 284 | 0 | 1.0308 | 1 |
|  |  |  |  |  |  |  | **122** | **GKECADL** | 1 | 312 | 1 | 1.0978 | 1 |
| **89** | 1,3-beta-glucanosyltransferase GAS5 | Saccharomyces cerevisiae | Q08193; D6W235 | [78] [31] | 8 | 1 | 71 | ASVCDRD | 0 | 301 | 1 | 1.0293 | 1 |
|  |  |  |  |  |  |  | 100 | HSHCMKL | 0 | 285 | 0 | 0.8550 | 0 |
|  |  |  |  |  |  |  | 129 | DPACSYN | 0 | 316 | 1 | 1.0223 | 1 |
|  |  |  |  |  |  |  | 215 | YFNCGDE | 0 | 305 | 1 | 1.0750 | 1 |
|  |  |  |  |  |  |  | 234 | YSWCGES | 0 | 302 | 1 | 0.9940 | 0 |
|  |  |  |  |  |  |  | **265** | **EFGCNQV** | 1 | 290 | 0 | 1.0868 | 1 |
|  |  |  |  |  |  |  | 347 | YSTCPDY | 0 | 287 | 0 | 1.1085 | 1 |
|  |  |  |  |  |  |  | 391 | QQSCDAK | 0 | 296 | 1 | 1.0320 | 1 |
| **90** | 1,3-beta-glucanosyltransferase GAS1 | Saccharomyces cerevisiae | P22146; D6W0D4; P23151 | [78] [31] | 14 | 3 | 74 | YESCSRD | 0 | 304 | 1 | 0.9940 | 0 |
|  |  |  |  |  |  |  | 103 | HSECMKA | 0 | 285 | 0 | 1.0035 | 1 |
|  |  |  |  |  |  |  | **216** | **YFACGDD** | 1 | 318 | 1 | 1.1003 | 1 |
|  |  |  |  |  |  |  | **234** | **YEWCGKS** | 1 | 304 | 1 | 0.9670 | 0 |
|  |  |  |  |  |  |  | 265 | EYGCNEV | 0 | 295 | 1 | 1.1058 | 1 |
|  |  |  |  |  |  |  | 347 | DVACPAT | 0 | 318 | 1 | 1.2100 | 1 |
|  |  |  |  |  |  |  | 370 | GGLCSCM | 0 | 311 | 1 | 0.7153 | 0 |
|  |  |  |  |  |  |  | 372 | LCSCMNA | 0 | 285 | 0 | 0.7515 | 0 |
|  |  |  |  |  |  |  | 379 | ANSCVVS | 0 | 305 | 1 | 1.1023 | 1 |
|  |  |  |  |  |  |  | 398 | NWICNEV | 0 | 275 | 0 | 1.0093 | 1 |
|  |  |  |  |  |  |  | 403 | EVDCSGI | 0 | 304 | 1 | 1.0690 | 1 |
|  |  |  |  |  |  |  | **421** | **YSFCTPK** | 1 | 296 | 1 | 1.0430 | 1 |
|  |  |  |  |  |  |  | 445 | KSDCSFS | 0 | 303 | 1 | 1.0343 | 1 |
|  |  |  |  |  |  |  | 462 | QASCSSA | 0 | 308 | 1 | 1.0003 | 1 |
| **91** | Homoaconitase, mitochondrial | Saccharomyces cerevisiae | P49367; D6VSL5; Q66RF2; Q7LH77 | [78] [31] [32] | 9 | 2 | 54 | PAHCMSH | 0 | 279 | 0 | 0.9358 | 0 |
|  |  |  |  |  |  |  | 202 | VALCGLF | 0 | 326 | 1 | 1.0770 | 1 |
|  |  |  |  |  |  |  | **340** | **LVSCTNS** | 1 | 300 | 1 | 1.0090 | 1 |
|  |  |  |  |  |  |  | **366** | **DVVCPTG** | 1 | 307 | 1 | 1.1608 | 1 |
|  |  |  |  |  |  |  | 400 | KAGCIPL | 0 | 313 | 1 | 1.2095 | 1 |
|  |  |  |  |  |  |  | 407 | PSGCGPC | 0 | 303 | 1 | 1.1700 | 1 |
|  |  |  |  |  |  |  | 410 | CGPCIGL | 0 | 291 | 0 | 1.0585 | 1 |
|  |  |  |  |  |  |  | 519 | LVLCDAD | 0 | 320 | 1 | 1.0248 | 1 |
|  |  |  |  |  |  |  | 549 | AQVCMEN | 0 | 294 | 0 | 1.0063 | 1 |
| **92** | Tryptophan synthase | Saccharomyces cerevisiae | P00931; D6VUB2 | [78] [31] [32] | 7 | 2 | 242 | VTLCGDA | 0 | 340 | 1 | 1.0425 | 1 |
|  |  |  |  |  |  |  | 321 | LHACLRE | 0 | 321 | 1 | 1.0318 | 1 |
|  |  |  |  |  |  |  | 365 | TEHCQGA | 0 | 288 | 0 | 0.8808 | 0 |
|  |  |  |  |  |  |  | **420** | **ATACAKF** | 1 | 326 | 1 | 1.1025 | 1 |
|  |  |  |  |  |  |  | 427 | GLTCTVF | 0 | 295 | 1 | 1.0663 | 1 |
|  |  |  |  |  |  |  | **528** | **VVACVGG** | 1 | 320 | 1 | 1.1408 | 1 |
|  |  |  |  |  |  |  | 657 | YGACELA | 0 | 331 | 1 | 1.1190 | 1 |
| **93** | Probable family 17 glucosidase SCW4 | Saccharomyces cerevisiae | P53334; D6VV56 | [78] [31] | 5 | 2 | **147** | **SGACKSA** | 1 | 327 | 1 | 1.0265 | 1 |
|  |  |  |  |  |  |  | **173** | **GTDCNQV** | 1 | 300 | 1 | 1.1605 | 1 |
|  |  |  |  |  |  |  | 275 | PELCDYS | 0 | 309 | 1 | 1.0010 | 1 |
|  |  |  |  |  |  |  | 313 | WTACDGK | 0 | 323 | 1 | 1.1323 | 1 |
|  |  |  |  |  |  |  | 354 | TSSCGAD | 0 | 310 | 1 | 1.0645 | 1 |
| **94** | Aminopeptidase Y | Saccharomyces cerevisiae | P37302; D6VQT1; P38154 | [78] [31] [32] | 4 | 4 | **187** | **NLGCEEK** | 1 | 313 | 1 | 1.1618 | 1 |
|  |  |  |  |  |  |  | **213** | **RGKCPFG** | 1 | 303 | 1 | 1.0718 | 1 |
|  |  |  |  |  |  |  | **470** | **LDRCYHQ** | 1 | 278 | 0 | 0.9380 | 0 |
|  |  |  |  |  |  |  | **475** | **HQLCDDV** | 1 | 288 | 0 | 0.9718 | 0 |
| **95** | Vacuolar fusion protein CCZ1 | Saccharomyces cerevisiae | P38273; D6VQC8 | [31] | 10 | 1 | 59 | SSCVNK | 0 | 284 | 0 | 0.8528 | 0 |
|  |  |  |  |  |  |  | 80 | IIFCIKV | 0 | 279 | 0 | 0.9678 | 0 |
|  |  |  |  |  |  |  | 261 | HLHCTYG | 0 | 284 | 0 | 0.9015 | 0 |
|  |  |  |  |  |  |  | 447 | FYNCLFW | 0 | 273 | 0 | 0.9665 | 0 |
|  |  |  |  |  |  |  | 461 | VIVCDPD | 0 | 298 | 1 | 1.1028 | 1 |
|  |  |  |  |  |  |  | 469 | NKICERD | 0 | 299 | 1 | 1.1400 | 1 |
|  |  |  |  |  |  |  | 482 | FQLCQSM | 0 | 283 | 0 | 0.8653 | 0 |
|  |  |  |  |  |  |  | 487 | SMECLNN | 0 | 288 | 0 | 0.9390 | 0 |
|  |  |  |  |  |  |  | 498 | SQNCDNV | 0 | 273 | 0 | 1.0028 | 1 |
|  |  |  |  |  |  |  | **645** | **GVLCYIK** | 1 | 307 | 1 | 0.9883 | 0 |
| **96** | Transketolase | Saccharomyces cerevisiae | E7KIQ8 | [31] | 2 | 1 | 159 | GDGCLQE | 0 | 315 | 1 | 1.1325 | 1 |
|  |  |  |  |  |  |  | **622** | **ATTCWGK** | 1 | 310 | 1 | 1.0295 | 1 |
| **97** | Zrt1p | Saccharomyces cerevisiae | E7Q3J7 | [31] | 6 | 1 | **31** | **WKTCVLQ** | 1 | 298 | 1 | 1.0673 | 1 |
|  |  |  |  |  |  |  | 116 | GNTCVGQ | 0 | 291 | 0 | 1.0868 | 1 |
|  |  |  |  |  |  |  | 129 | YSWCPAI | 0 | 285 | 0 | 1.0203 | 1 |
|  |  |  |  |  |  |  | 284 | WALCVAY | 0 | 303 | 1 | 1.1063 | 1 |
|  |  |  |  |  |  |  | 294 | TPICVAI | 0 | 304 | 1 | 1.1315 | 1 |
|  |  |  |  |  |  |  | 359 | NVICTLF | 0 | 298 | 1 | 1.0663 | 1 |
| **98** | Ssb1p | Saccharomyces cerevisiae | E7NFG0 | [31] | 3 | 1 | 20 | TYSCVAT | 0 | 296 | 1 | 1.0315 | 1 |
|  |  |  |  |  |  |  | **435** | **FTTCADN** | 1 | 305 | 1 | 1.0593 | 1 |
|  |  |  |  |  |  |  | 464 | RVNCKEN | 0 | 298 | 1 | 1.0818 | 1 |
| **99** | Dna2p | Saccharomyces cerevisiae | C7GJX6 | [31] | 25 | 1 | 305 | QESCGKN | 0 | 308 | 1 | 1.0705 | 1 |
|  |  |  |  |  |  |  | 342 | DSNCQRI | 0 | 269 | 0 | 0.9808 | 0 |
|  |  |  |  |  |  |  | 443 | ILECIDG | 0 | 295 | 1 | 1.0353 | 1 |
|  |  |  |  |  |  |  | 519 | SVGCLRR | 0 | 311 | 1 | 1.0603 | 1 |
|  |  |  |  |  |  |  | 586 | IIICNEE | 0 | 299 | 1 | 1.1285 | 1 |
|  |  |  |  |  |  |  | 620 | NYGCYTS | 0 | 301 | 1 | 1.0400 | 1 |
|  |  |  |  |  |  |  | 768 | DSSCDSC | 0 | 285 | 0 | 1.0000 | 0 |
|  |  |  |  |  |  |  | 771 | CDSCFIK | 0 | 278 | 0 | 1.1265 | 1 |
|  |  |  |  |  |  |  | 777 | KESCMVL | 0 | 304 | 1 | 1.0458 | 1 |
|  |  |  |  |  |  |  | 833 | SITCVNK | 0 | 295 | 1 | 1.0333 | 1 |
|  |  |  |  |  |  |  | 852 | SGRCLSG | 0 | 309 | 1 | 0.8330 | 0 |
|  |  |  |  |  |  |  | 875 | YIYCFSR | 0 | 284 | 0 | 0.9950 | 0 |
|  |  |  |  |  |  |  | 909 | GHFCLCQ | 0 | 281 | 0 | 0.7035 | 0 |
|  |  |  |  |  |  |  | 911 | FCLCQGR | 0 | 287 | 0 | 0.7330 | 0 |
|  |  |  |  |  |  |  | 1008 | SKLCRKT | 0 | 311 | 1 | 0.9198 | 0 |
|  |  |  |  |  |  |  | 1165 | ATTCLGI | 0 | 315 | 1 | 1.0145 | 1 |
|  |  |  |  |  |  |  | 1238 | KTFCEKH | 0 | 298 | 1 | 1.0325 | 1 |
|  |  |  |  |  |  |  | 1255 | YRMCGDI | 0 | 286 | 0 | 0.9483 | 0 |
|  |  |  |  |  |  |  | 1272 | KLKCGNN | 0 | 309 | 1 | 0.9358 | 0 |
|  |  |  |  |  |  |  | 1323 | YDNCPDI | 0 | 283 | 0 | 1.2455 | 1 |
|  |  |  |  |  |  |  | 1347 | TLQCVEG | 0 | 299 | 1 | 1.0728 | 1 |
|  |  |  |  |  |  |  | 1358 | GVPCEDI | 0 | 290 | 0 | 1.1080 | 1 |
|  |  |  |  |  |  |  | 1401 | DKKCIII | 0 | 282 | 0 | 1.0200 | 1 |
|  |  |  |  |  |  |  | **1468** | **YTMCKDA** | 1 | 305 | 1 | 0.9765 | 0 |
|  |  |  |  |  |  |  | 1491 | RKGCGKR | 0 | 320 | 1 | 1.1278 | 1 |
| **100** | Plasma membrane ATPase | Saccharomyces cerevisiae | E7Q3Y4 | [31] | 10 | 2 | 148 | GVICGLL | 0 | 333 | 1 | 1.0805 | 1 |
|  |  |  |  |  |  |  | **221** | **TEDCFLQ** | 1 | 302 | 1 | 1.1020 | 1 |
|  |  |  |  |  |  |  | 312 | WTACFYR | 0 | 306 | 1 | 1.0623 | 1 |
|  |  |  |  |  |  |  | 376 | EILCSDK | 0 | 322 | 1 | 0.9245 | 0 |
|  |  |  |  |  |  |  | **409** | **LTACLAA** | 1 | 344 | 1 | 1.0723 | 1 |
|  |  |  |  |  |  |  | 472 | RIVCVKG | 0 | 299 | 1 | 1.0483 | 1 |
|  |  |  |  |  |  |  | 532 | VMPCMDP | 0 | 280 | 0 | 0.9735 | 0 |
|  |  |  |  |  |  |  | 569 | KETCRQL | 0 | 302 | 1 | 0.9925 | 0 |
|  |  |  |  |  |  |  | 828 | LAGCRLR | 0 | 312 | 1 | 1.0670 | 1 |
|  |  |  |  |  |  |  | 832 | RLRCRHH | 0 | 282 | 0 | 0.7885 | 0 |
| **101** | Ski2p | Saccharomyces cerevisiae | E7Q7B5 | [31] | 12 | 1 | 82 | PEDCSGK | 0 | 311 | 1 | 1.0843 | 1 |
|  |  |  |  |  |  |  | 416 | DANCLIM | 0 | 283 | 0 | 1.1088 | 1 |
|  |  |  |  |  |  |  | 639 | KKRCEEY | 0 | 296 | 1 | 1.0295 | 1 |
|  |  |  |  |  |  |  | 652 | INFCNNK | 0 | 276 | 0 | 0.9455 | 0 |
|  |  |  |  |  |  |  | 861 | YKSCEIC | 0 | 284 | 0 | 1.1290 | 1 |
|  |  |  |  |  |  |  | 864 | CEICDND | 0 | 284 | 0 | 1.0370 | 1 |
|  |  |  |  |  |  |  | **913** | **PNDCLKL** | 1 | 304 | 1 | 1.0580 | 1 |
|  |  |  |  |  |  |  | 929 | DAVCVIM | 0 | 285 | 0 | 1.1505 | 1 |
|  |  |  |  |  |  |  | 1054 | SIKCPNL | 0 | 303 | 1 | 1.0385 | 1 |
|  |  |  |  |  |  |  | 1119 | RVACEIN | 0 | 306 | 1 | 1.1808 | 1 |
|  |  |  |  |  |  |  | 1187 | KMLCVFN | 0 | 300 | 1 | 0.9895 | 0 |
|  |  |  |  |  |  |  | 1249 | DEICREV | 0 | 295 | 1 | 1.0545 | 1 |
| **102** | Polyadenylate-binding protein, cytoplasmic and nuclear | Saccharomyces cerevisiae | P04147; D3DM73 | [31] | 3 | 1 | 70 | IRVCRDA | 0 | 293 | 0 | 0.8565 | 0 |
|  |  |  |  |  |  |  | 109 | GRLCRIM | 0 | 292 | 0 | 0.8685 | 0 |
|  |  |  |  |  |  |  | **368** | **GFVCFST** | 1 | 293 | 0 | 0.9538 | 0 |
| **103** | Lys9p | Saccharomyces cerevisiae | E7KHL0 | [31] | 4 | 1 | 33 | TVACRTL | 0 | 321 | 1 | 1.0628 | 1 |
|  |  |  |  |  |  |  | **154** | **LSYCGGL** | 1 | 310 | 1 | 1.0590 | 1 |
|  |  |  |  |  |  |  | 219 | AFVCYPN | 0 | 299 | 1 | 1.0163 | 1 |
|  |  |  |  |  |  |  | 340 | DTLCARL | 0 | 330 | 1 | 1.0390 | 1 |
| **104** | Ilv3p | Saccharomyces cerevisiae | E7Q5M5 | [31] | 10 | 1 | 18 | TTRCVAK | 0 | 317 | 1 | 0.9810 | 0 |
|  |  |  |  |  |  |  | 63 | VGSCWWS | 0 | 285 | 0 | 0.9198 | 0 |
|  |  |  |  |  |  |  | 70 | GNPCNMH | 0 | 266 | 0 | 1.0333 | 1 |
|  |  |  |  |  |  |  | 81 | NNRCSQS | 0 | 293 | 0 | 0.8495 | 0 |
|  |  |  |  |  |  |  | **143** | **IPSCDKN** | 1 | 303 | 1 | 1.0493 | 1 |
|  |  |  |  |  |  |  | 176 | PGSCGGM | 0 | 307 | 1 | 1.0738 | 1 |
|  |  |  |  |  |  |  | 215 | EHACPGP | 0 | 314 | 1 | 1.1778 | 1 |
|  |  |  |  |  |  |  | 221 | PGSCGGM | 0 | 307 | 1 | 1.0738 | 1 |
|  |  |  |  |  |  |  | 258 | LAECDNI | 0 | 285 | 0 | 1.1055 | 1 |
|  |  |  |  |  |  |  | 581 | SNGCVLD | 0 | 311 | 1 | 1.1105 | 1 |
| **105** | Asc1p | Saccharomyces cerevisiae | E7Q7T5 | [31] | 3 | 1 | 71 | VQDCTLT | 0 | 296 | 1 | 1.1065 | 1 |
|  |  |  |  |  |  |  | **140** | **KGQCLAT** | 1 | 303 | 1 | 0.9788 | 0 |
|  |  |  |  |  |  |  | 181 | VKVCDIF | 0 | 288 | 0 | 1.0790 | 1 |
| **106** | Rpl10p | Saccharomyces cerevisiae | E7Q6J6 | [31] | 4 | 1 | 8 | PARCYRY | 0 | 279 | 0 | 0.9525 | 0 |
|  |  |  |  |  |  |  | **49** | **FPLCVHL** | 1 | 317 | 1 | 1.0300 | 1 |
|  |  |  |  |  |  |  | 71 | ARICANK | 0 | 302 | 1 | 0.9300 | 0 |
|  |  |  |  |  |  |  | 105 | MLSCAGA | 0 | 300 | 1 | 1.0198 | 1 |
| **107** | Pro2p | Saccharomyces cerevisiae | E7Q9K3 | [31] | 3 | 1 | 230 | DGICSIY | 0 | 292 | 0 | 1.0095 | 1 |
|  |  |  |  |  |  |  | **258** | **PAGCNAM** | 1 | 297 | 1 | 1.1840 | 1 |
|  |  |  |  |  |  |  | 314 | AIQCKTV | 0 | 295 | 1 | 1.0330 | 1 |
| **108** | Csf1p | Saccharomyces cerevisiae | C8ZD57 | [31] | 30 | 1 | 33 | VVVCLTM | 0 | 305 | 1 | 1.0093 | 1 |
|  |  |  |  |  |  |  | 108 | LLNCRKA | 0 | 300 | 1 | 0.9463 | 0 |
|  |  |  |  |  |  |  | 128 | KLPCKI | 0 | 282 | 0 | 1.0210 | 1 |
|  |  |  |  |  |  |  | 135 | SVECEGL | 0 | 308 | 1 | 1.1490 | 1 |
|  |  |  |  |  |  |  | 382 | ILKCPKV | 0 | 301 | 1 | 1.0270 | 1 |
|  |  |  |  |  |  |  | 434 | GSICYGP | 0 | 296 | 1 | 1.0058 | 1 |
|  |  |  |  |  |  |  | 522 | LRFCKDT | 0 | 286 | 0 | 0.8470 | 0 |
|  |  |  |  |  |  |  | 534 | ISVCPTV | 0 | 297 | 1 | 1.1363 | 1 |
|  |  |  |  |  |  |  | 661 | NENCYLS | 0 | 299 | 1 | 1.0255 | 1 |
|  |  |  |  |  |  |  | 748 | VIECNSK | 0 | 292 | 0 | 1.0833 | 1 |
|  |  |  |  |  |  |  | 757 | VLHCYGF | 0 | 286 | 0 | 0.8698 | 0 |
|  |  |  |  |  |  |  | 866 | FDPCIAL | 0 | 295 | 1 | 1.1583 | 1 |
|  |  |  |  |  |  |  | 881 | FRSCNYY | 0 | 267 | 0 | 0.9320 | 0 |
|  |  |  |  |  |  |  | 947 | TYFCQWD | 0 | 263 | 0 | 0.7843 | 0 |
|  |  |  |  |  |  |  | 957 | GDLCIDS | 0 | 310 | 1 | 1.0690 | 1 |
|  |  |  |  |  |  |  | 1055 | SLNCVMG | 0 | 293 | 0 | 1.0263 | 1 |
|  |  |  |  |  |  |  | 1104 | YHRCPFL | 0 | 293 | 0 | 0.9895 | 0 |
|  |  |  |  |  |  |  | 1388 | FEYCSDQ | 0 | 289 | 0 | 0.9683 | 0 |
|  |  |  |  |  |  |  | 1399 | QEVCTSF | 0 | 290 | 0 | 1.0448 | 1 |
|  |  |  |  |  |  |  | 1539 | LLKCTMG | 0 | 299 | 1 | 0.9300 | 0 |
|  |  |  |  |  |  |  | 1726 | NNQCADL | 0 | 288 | 0 | 1.0290 | 1 |
|  |  |  |  |  |  |  | 1781 | VLSCYFS | 0 | 298 | 1 | 0.9573 | 0 |
|  |  |  |  |  |  |  | 2022 | FRVCLSP | 0 | 295 | 1 | 0.8603 | 0 |
|  |  |  |  |  |  |  | 2084 | YKFCIGW | 0 | 282 | 0 | 1.0000 | 0 |
|  |  |  |  |  |  |  | 2162 | FKRCGEL | 0 | 318 | 1 | 1.0158 | 1 |
|  |  |  |  |  |  |  | 2281 | TINCTYK | 0 | 286 | 0 | 1.0793 | 1 |
|  |  |  |  |  |  |  | 2312 | YAGCAPL | 0 | 317 | 1 | 1.2255 | 1 |
|  |  |  |  |  |  |  | **2372** | **SLSCEPK** | 1 | 312 | 1 | 1.1093 | 1 |
|  |  |  |  |  |  |  | 2513 | IPWCFTL | 0 | 301 | 1 | 0.9650 | 0 |
|  |  |  |  |  |  |  | 2831 | LFACSFS | 0 | 317 | 1 | 0.9980 | 0 |
| **109** | Adh3p | Saccharomyces cerevisiae | E7Q7R5 | [31] | 6 | 2 | **71** | **SGVCHTD** | 1 | 300 | 1 | 0.9508 | 0 |
|  |  |  |  |  |  |  | 125 | GSCMTC | 0 | 281 | 0 | 0.6838 | 0 |
|  |  |  |  |  |  |  | 128 | CMTCEFC | 0 | 262 | 0 | 1.0538 | 1 |
|  |  |  |  |  |  |  | 131 | CEFCESG | 0 | 287 | 0 | 1.0310 | 1 |
|  |  |  |  |  |  |  | **181** | **PILCAGV** | 1 | 303 | 1 | 1.0268 | 1 |
|  |  |  |  |  |  |  | 290 | VRPCGTV | 0 | 300 | 1 | 1.0478 | 1 |
| **110** | Ymr295cp | Saccharomyces cerevisiae | G2WL04 | [31] | 1 | 1 | **82** | **HVLCDVF** | 1 | 296 | 1 | 1.0443 | 1 |
| **111** | Peptidyl-prolyl cis-trans isomerase | Saccharomyces cerevisiae | P14832; D6VSD6 | [31] [32] | 2 | 2 | **48** | **RALCTGE** | 1 | 321 | 1 | 1.0930 | 1 |
|  |  |  |  |  |  |  | **117** | **TVPCPWL** | 1 | 292 | 0 | 1.0960 | 1 |
| **112** | Aro4p | Saccharomyces cerevisiae | E7Q1A1 | [31] | 3 | 1 | **76** | **VGPCSIH** | 1 | 288 | 0 | 1.0123 | 1 |
|  |  |  |  |  |  |  | 215 | VDACQAA | 0 | 311 | 1 | 1.1140 | 1 |
|  |  |  |  |  |  |  | 244 | NEHCFVI | 0 | 280 | 0 | 0.9628 | 0 |
| **113** | Dse3p | Saccharomyces cerevisiae | E7Q9E5 | [31] | 8 | 2 | 108 | GPVCPQS | 0 | 313 | 1 | 1.1503 | 1 |
|  |  |  |  |  |  |  | **190** | **TQDCCRK** | 1 | 283 | 0 | 0.9505 | 0 |
|  |  |  |  |  |  |  | **191** | **QDCCRKN** | 1 | 279 | 0 | 0.8795 | 0 |
|  |  |  |  |  |  |  | 234 | IDICASK | 0 | 303 | 1 | 1.1348 | 1 |
|  |  |  |  |  |  |  | 253 | YVVCPDD | 0 | 300 | 1 | 1.1160 | 1 |
|  |  |  |  |  |  |  | 290 | TSGCSTY | 0 | 303 | 1 | 1.0368 | 1 |
|  |  |  |  |  |  |  | 333 | EGYCTAA | 0 | 309 | 1 | 1.0630 | 1 |
|  |  |  |  |  |  |  | 363 | TSSCNTL | 0 | 298 | 1 | 1.0588 | 1 |
| **114** | Eno2p | Saccharomyces cerevisiae | E7Q4V3 | [31] | 1 | 1 | **248** | **GLDCASS** | 1 | 314 | 1 | 1.0833 | 1 |
| **115** | Rpl35ap | Saccharomyces cerevisiae | C8Z6B2 | [31] | 1 | 1 | **53** | **SIACVLT** | 1 | 319 | 1 | 1.1175 | 1 |
| **116** | Tfa1p | Saccharomyces cerevisiae | E7LWX3 | [31] | 4 | 1 | 2 | --MCPIC | 0 | 249 | 0 | 1.1690 | 1 |
|  |  |  |  |  |  |  | 5 | CPICLTK | 0 | 309 | 1 | 1.0658 | 1 |
|  |  |  |  |  |  |  | **27** | **EFLCSLC** | 1 | 316 | 1 | 0.8798 | 0 |
|  |  |  |  |  |  |  | 30 | CSLCDEP | 0 | 300 | 1 | 1.0333 | 1 |
| **117** | Protein HRB1 | Saccharomyces cerevisiae | P38922; A2TBM5; D6W1H3; Q06HN4 | [31] | 4 | 1 | 284 | FKECGNV | 0 | 296 | 1 | 1.0840 | 1 |
|  |  |  |  |  |  |  | 380 | LIYCSNL | 0 | 297 | 1 | 0.9180 | 0 |
|  |  |  |  |  |  |  | 432 | ADVCIER | 0 | 298 | 1 | 1.1538 | 1 |
|  |  |  |  |  |  |  | **444** | **YGGCDLD** | 1 | 317 | 1 | 1.0783 | 1 |
| **118** | Carboxypeptidase | Saccharomyces cerevisiae | E7KT38 | [31] | 12 | 2 | 9 | SLLCGLG | 0 | 340 | 1 | 0.9813 | 0 |
|  |  |  |  |  |  |  | 167 | GPGCSSL | 0 | 329 | 1 | 1.0253 | 1 |
|  |  |  |  |  |  |  | 304 | PMACGEG | 0 | 319 | 1 | 1.1410 | 1 |
|  |  |  |  |  |  |  | 318 | SEECSAM | 0 | 307 | 1 | 1.0438 | 1 |
|  |  |  |  |  |  |  | 328 | LERCLGL | 0 | 313 | 1 | 0.8913 | 0 |
|  |  |  |  |  |  |  | 335 | IESCYDS | 0 | 299 | 1 | 1.0000 | 0 |
|  |  |  |  |  |  |  | 344 | VWSCVPA | 0 | 297 | 1 | 1.0143 | 1 |
|  |  |  |  |  |  |  | 351 | YESCNFD | 0 | 285 | 0 | 1.0628 | 1 |
|  |  |  |  |  |  |  | **373** | **RKDCEGG** | 1 | 308 | 1 | 1.2040 | 1 |
|  |  |  |  |  |  |  | **379** | **GNLCYPT** | 1 | 302 | 1 | 1.0143 | 1 |
|  |  |  |  |  |  |  | 409 | YESCNFD | 0 | 285 | 0 | 1.0628 | 1 |
|  |  |  |  |  |  |  | 452 | DFICNWL | 0 | 283 | 0 | 0.9805 | 0 |
| **119** | Ubiquitin-activating enzyme E1 1 | Saccharomyces cerevisiae | P22515; D6VWZ3 | [31] [32] | 10 | 2 | 144 | NEFCHSS | 0 | 288 | 0 | 0.8773 | 0 |
|  |  |  |  |  |  |  | 378 | LKACSGK | 0 | 333 | 1 | 1.0450 | 1 |
|  |  |  |  |  |  |  | **447** | **AIGCEML** | 1 | 309 | 1 | 1.1150 | 1 |
|  |  |  |  |  |  |  | 503 | EAVCAMN | 0 | 295 | 1 | 1.0543 | 1 |
|  |  |  |  |  |  |  | 556 | DRRCVFY | 0 | 275 | 0 | 0.8613 | 0 |
|  |  |  |  |  |  |  | **600** | **IPLCTLR** | 1 | 323 | 1 | 1.0253 | 1 |
|  |  |  |  |  |  |  | 672 | FEDCIKW | 0 | 282 | 0 | 1.1220 | 1 |
|  |  |  |  |  |  |  | 837 | ITACSNC | 0 | 314 | 1 | 0.9875 | 0 |
|  |  |  |  |  |  |  | 840 | CSNCRAQ | 0 | 276 | 0 | 0.9920 | 0 |
|  |  |  |  |  |  |  | 1006 | LEICADD | 0 | 304 | 1 | 1.0785 | 1 |
| **120** | NADH dehydrogenase [ubiquinone] flavoprotein 1, mitochondrial | Rattus norvegicus | Q5XIH3 | [37] [79] [7] | 12 | 1 | 125 | PGTCKDR | 0 | 296 | 1 | 0.9863 | 0 |
|  |  |  |  |  |  |  | 142 | VEGCLVG | 0 | 315 | 1 | 1.0940 | 1 |
|  |  |  |  |  |  |  | 187 | NHPCTVE | 0 | 289 | 0 | 1.0955 | 1 |
|  |  |  |  |  |  |  | **206** | **AYICGEE** | 1 | 325 | 1 | 1.0963 | 1 |
|  |  |  |  |  |  |  | 238 | VFGCPTT | 0 | 306 | 1 | 1.2050 | 1 |
|  |  |  |  |  |  |  | 255 | PTICRRG | 0 | 307 | 1 | 1.0210 | 1 |
|  |  |  |  |  |  |  | 286 | NHPCTVE | 0 | 289 | 0 | 1.0955 | 1 |
|  |  |  |  |  |  |  | 332 | KSVCETV | 0 | 296 | 1 | 1.0908 | 1 |
|  |  |  |  |  |  |  | 379 | HESCGQC | 0 | 292 | 0 | 1.0930 | 1 |
|  |  |  |  |  |  |  | 382 | CGQCTPC | 0 | 276 | 0 | 1.0998 | 1 |
|  |  |  |  |  |  |  | 385 | CTPCREG | 0 | 296 | 1 | 1.0558 | 1 |
|  |  |  |  |  |  |  | 425 | HTICALG | 0 | 317 | 1 | 1.0660 | 1 |
| **121** | - | - | F8WG21 | [17] [80] [7] | 18 | 1 | **89** | **NTACLTK** | 1 | 335 | 1 | 1.0933 | 1 |
|  |  |  |  |  |  |  | 189 | AHRCCCV | 0 | 273 | 0 | 0.5238 | 0 |
|  |  |  |  |  |  |  | 190 | HRCCCVA | 0 | 273 | 0 | 0.6600 | 0 |
|  |  |  |  |  |  |  | 191 | RCCCVAD | 0 | 288 | 0 | 0.7385 | 0 |
|  |  |  |  |  |  |  | 231 | NGECRGV | 0 | 287 | 0 | 1.0155 | 1 |
|  |  |  |  |  |  |  | 238 | IALCIED | 0 | 309 | 1 | 1.1005 | 1 |
|  |  |  |  |  |  |  | 266 | YFSCTSA | 0 | 300 | 1 | 1.0113 | 1 |
|  |  |  |  |  |  |  | 287 | GLPCQDL | 0 | 297 | 1 | 0.9365 | 0 |
|  |  |  |  |  |  |  | 305 | GAGCLIT | 0 | 302 | 1 | 1.1348 | 1 |
|  |  |  |  |  |  |  | 311 | TEGCRGE | 0 | 310 | 1 | 1.0533 | 1 |
|  |  |  |  |  |  |  | 357 | GRGCGPE | 0 | 324 | 1 | 1.1033 | 1 |
|  |  |  |  |  |  |  | 438 | LYACGEA | 0 | 331 | 1 | 1.1318 | 1 |
|  |  |  |  |  |  |  | 443 | EAACASV | 0 | 315 | 1 | 1.1450 | 1 |
|  |  |  |  |  |  |  | 467 | GRACALS | 0 | 327 | 1 | 0.9965 | 0 |
|  |  |  |  |  |  |  | 475 | AESCRPG | 0 | 308 | 1 | 1.0383 | 1 |
|  |  |  |  |  |  |  | 536 | QEGCEKV | 0 | 299 | 1 | 1.1468 | 1 |
|  |  |  |  |  |  |  | 574 | LMLCALQ | 0 | 311 | 1 | 0.9468 | 0 |
|  |  |  |  |  |  |  | 654 | EADCATV | 0 | 304 | 1 | 1.2368 | 1 |
| **122** | Ryanodine receptor 2 | Mus musculus | E9Q401; O70181; Q62174; Q62197; Q9ERN6 | [81] [82] [83] [84] [85] [86] [7] | 91 | 7 | 24 | VLQCTAT | 0 | 298 | 1 | 1.0310 | 1 |
|  |  |  |  |  |  |  | **36** | **QKLCLAA** | 1 | 329 | 1 | 0.9703 | 0 |
|  |  |  |  |  |  |  | 47 | NRLCFLE | 0 | 316 | 1 | 0.8633 | 0 |
|  |  |  |  |  |  |  | 65 | LSICTFV | 0 | 289 | 0 | 1.0618 | 1 |
|  |  |  |  |  |  |  | 131 | MYLCCLS | 0 | 303 | 1 | 0.7598 | 0 |
|  |  |  |  |  |  |  | 132 | YLCCLST | 0 | 296 | 1 | 0.7723 | 0 |
|  |  |  |  |  |  |  | 158 | GEACWWT | 0 | 291 | 0 | 1.0075 | 1 |
|  |  |  |  |  |  |  | 244 | MDECLTV | 0 | 286 | 0 | 1.1530 | 1 |
|  |  |  |  |  |  |  | 361 | DSICYIQ | 0 | 277 | 0 | 1.0265 | 1 |
|  |  |  |  |  |  |  | 501 | VLECIDR | 0 | 293 | 0 | 1.0353 | 1 |
|  |  |  |  |  |  |  | 548 | RKNCAQF | 0 | 289 | 0 | 1.0960 | 1 |
|  |  |  |  |  |  |  | 577 | VLHCVLV | 0 | 303 | 1 | 0.9145 | 0 |
|  |  |  |  |  |  |  | 615 | DVLCSLC | 0 | 316 | 1 | 0.9065 | 0 |
|  |  |  |  |  |  |  | 618 | CSLCVCH | 0 | 282 | 0 | 0.8073 | 0 |
|  |  |  |  |  |  |  | 620 | LCVCHGV | 0 | 280 | 0 | 0.7423 | 0 |
|  |  |  |  |  |  |  | 633 | HLICDNL | 0 | 298 | 1 | 0.9853 | 0 |
|  |  |  |  |  |  |  | 736 | WSGCIAR | 0 | 293 | 0 | 1.0828 | 1 |
|  |  |  |  |  |  |  | 757 | VISCCLD | 0 | 296 | 1 | 0.8553 | 0 |
|  |  |  |  |  |  |  | 758 | ISCCLDL | 0 | 299 | 1 | 0.7973 | 0 |
|  |  |  |  |  |  |  | **822** | **YAACYEA** | 1 | 317 | 1 | 1.1625 | 1 |
|  |  |  |  |  |  |  | **917** | **QHPCLVE** | 1 | 295 | 1 | 1.0175 | 1 |
|  |  |  |  |  |  |  | 922 | VEFCKLP | 0 | 305 | 1 | 0.9425 | 0 |
|  |  |  |  |  |  |  | 948 | ALGCHVG | 0 | 310 | 1 | 0.9945 | 0 |
|  |  |  |  |  |  |  | 1078 | AEVCSGT | 0 | 311 | 1 | 0.9800 | 0 |
|  |  |  |  |  |  |  | 1122 | RPGCQPD | 0 | 301 | 1 | 1.0950 | 1 |
|  |  |  |  |  |  |  | 1164 | VVGCMVF | 0 | 294 | 0 | 1.0930 | 1 |
|  |  |  |  |  |  |  | 1215 | IPVCSLG | 0 | 320 | 1 | 0.9563 | 0 |
|  |  |  |  |  |  |  | 1230 | FTICGLQ | 0 | 324 | 1 | 1.0943 | 1 |
|  |  |  |  |  |  |  | 1282 | SSPCLKV | 0 | 293 | 0 | 0.9675 | 0 |
|  |  |  |  |  |  |  | 1310 | PIECAEV | 0 | 289 | 0 | 1.1515 | 1 |
|  |  |  |  |  |  |  | 1485 | RSNCYMV | 0 | 271 | 0 | 0.9633 | 0 |
|  |  |  |  |  |  |  | 1489 | YMVCAGE | 0 | 299 | 1 | 1.0050 | 1 |
|  |  |  |  |  |  |  | 1510 | EIGCVVD | 0 | 305 | 1 | 1.1725 | 1 |
|  |  |  |  |  |  |  | **1583** | **VPQCPPR** | 1 | 300 | 1 | 1.2058 | 1 |
|  |  |  |  |  |  |  | 1622 | LVQCLDP | 0 | 297 | 1 | 0.9658 | 0 |
|  |  |  |  |  |  |  | 1666 | SAVCALG | 0 | 314 | 1 | 1.0768 | 1 |
|  |  |  |  |  |  |  | 1678 | HALCSHV | 0 | 301 | 1 | 0.9618 | 0 |
|  |  |  |  |  |  |  | 1776 | SNDCYQY | 0 | 281 | 0 | 1.0923 | 1 |
|  |  |  |  |  |  |  | 1906 | LQMCLLL | 0 | 303 | 1 | 0.9070 | 0 |
|  |  |  |  |  |  |  | 1913 | QYLCDCQ | 0 | 290 | 0 | 0.7575 | 0 |
|  |  |  |  |  |  |  | 1915 | LCDCQVR | 0 | 279 | 0 | 0.8893 | 0 |
|  |  |  |  |  |  |  | 1985 | KSECPCP | 0 | 287 | 0 | 0.9593 | 0 |
|  |  |  |  |  |  |  | 1977 | ECPCPEE | 0 | 301 | 1 | 1.0165 | 1 |
|  |  |  |  |  |  |  | 2006 | MTHCGIE | 0 | 304 | 1 | 1.0213 | 1 |
|  |  |  |  |  |  |  | 2054 | SRKCSSL | 0 | 312 | 1 | 0.7988 | 0 |
|  |  |  |  |  |  |  | 2195 | VANCCRF | 0 | 277 | 0 | 0.9798 | 0 |
|  |  |  |  |  |  |  | 2196 | ANCCRFL | 0 | 290 | 0 | 0.8133 | 0 |
|  |  |  |  |  |  |  | 2000 | RFLCYFC | 0 | 287 | 0 | 0.9305 | 0 |
|  |  |  |  |  |  |  | 2203 | NYFCRIS | 0 | 276 | 0 | 0.8940 | 0 |
|  |  |  |  |  |  |  | 2271 | LAGCGLQ | 0 | 324 | 1 | 1.1760 | 1 |
|  |  |  |  |  |  |  | 2276 | LQSCQML | 0 | 279 | 0 | 0.8813 | 0 |
|  |  |  |  |  |  |  | 2307 | AVFCNGE | 0 | 301 | 1 | 1.0075 | 1 |
|  |  |  |  |  |  |  | **2329** | **RPECFGP** | 1 | 299 | 1 | 1.0938 | 1 |
|  |  |  |  |  |  |  | 2401 | LGRCAPE | 0 | 311 | 1 | 0.9768 | 0 |
|  |  |  |  |  |  |  | 2460 | AGFCPDH | 0 | 298 | 1 | 1.0635 | 1 |
|  |  |  |  |  |  |  | 2520 | RYLCTAV | 0 | 309 | 1 | 0.9863 | 0 |
|  |  |  |  |  |  |  | 2530 | LTRCAPL | 0 | 320 | 1 | 1.0013 | 1 |
|  |  |  |  |  |  |  | 2558 | SKGCSLT | 0 | 324 | 1 | 1.0155 | 1 |
|  |  |  |  |  |  |  | 2571 | IEVCLLS | 0 | 319 | 1 | 0.9688 | 0 |
|  |  |  |  |  |  |  | 2576 | LSICGQL | 0 | 317 | 1 | 1.0795 | 1 |
|  |  |  |  |  |  |  | 2616 | YERCWKY | 0 | 276 | 0 | 0.8770 | 0 |
|  |  |  |  |  |  |  | 2621 | KYYCLPG | 0 | 297 | 1 | 0.9868 | 0 |
|  |  |  |  |  |  |  | 2667 | ALPCLSA | 0 | 316 | 1 | 0.9525 | 0 |
|  |  |  |  |  |  |  | 2990 | RPLCTGG | 0 | 320 | 1 | 1.0555 | 1 |
|  |  |  |  |  |  |  | 3008 | SLFCKLG | 0 | 311 | 1 | 0.8908 | 0 |
|  |  |  |  |  |  |  | 3031 | IVNCLHI | 0 | 286 | 0 | 0.9945 | 0 |
|  |  |  |  |  |  |  | 3129 | QVSCYRI | 0 | 283 | 0 | 1.0095 | 1 |
|  |  |  |  |  |  |  | **3157** | **LGECLAA** | 1 | 318 | 1 | 1.0368 | 1 |
|  |  |  |  |  |  |  | 3204 | EDVCPNI | 0 | 287 | 0 | 1.1585 | 1 |
|  |  |  |  |  |  |  | 3242 | PMLCSYM | 0 | 295 | 1 | 0.8595 | 0 |
|  |  |  |  |  |  |  | 3263 | AEMCCTA | 0 | 298 | 1 | 0.8935 | 0 |
|  |  |  |  |  |  |  | 3264 | EMCCTAL | 0 | 300 | 1 | 0.8808 | 0 |
|  |  |  |  |  |  |  | 3480 | LNICAPG | 0 | 302 | 1 | 1.1293 | 1 |
|  |  |  |  |  |  |  | **3601** | **VVACFRM** | 1 | 308 | 1 | 1.0885 | 1 |
|  |  |  |  |  |  |  | 3679 | TEKCKLE | 0 | 317 | 1 | 0.9418 | 0 |
|  |  |  |  |  |  |  | 3698 | AKSCHDE | 0 | 309 | 1 | 0.9353 | 0 |
|  |  |  |  |  |  |  | 3799 | MQSCSVL | 0 | 292 | 0 | 0.9680 | 0 |
|  |  |  |  |  |  |  | 3836 | EFTCDLF | 0 | 297 | 1 | 1.0083 | 1 |
|  |  |  |  |  |  |  | 3846 | QLLCEGH | 0 | 305 | 1 | 1.0253 | 1 |
|  |  |  |  |  |  |  | 3926 | QGPCTGN | 0 | 295 | 1 | 1.0885 | 1 |
|  |  |  |  |  |  |  | 4068 | LLSCAET | 0 | 305 | 1 | 1.0573 | 1 |
|  |  |  |  |  |  |  | 4192 | VNFCEDT | 0 | 283 | 0 | 1.0280 | 1 |
|  |  |  |  |  |  |  | 4301 | ASVCRGF | 0 | 292 | 0 | 0.9425 | 0 |
|  |  |  |  |  |  |  | 4585 | SFFCIIG | 0 | 279 | 0 | 0.9840 | 0 |
|  |  |  |  |  |  |  | 4591 | GYYCLKV | 0 | 290 | 0 | 0.9100 | 0 |
|  |  |  |  |  |  |  | 4805 | DMKCDDM | 0 | 284 | 0 | 0.9438 | 0 |
|  |  |  |  |  |  |  | 4811 | MLTCYMF | 0 | 269 | 0 | 0.9233 | 0 |
|  |  |  |  |  |  |  | 4887 | ETKCFIC | 0 | 294 | 0 | 1.0048 | 1 |
|  |  |  |  |  |  |  | 4890 | CFICGIG | 0 | 290 | 0 | 1.1048 | 1 |
|  |  |  |  |  |  |  | 4947 | QERCWEF | 0 | 276 | 0 | 0.9438 | 0 |
|  |  |  |  |  |  |  | 4956 | AGDCFRK | 0 | 313 | 1 | 1.1118 | 1 |
| **123** | Thymidine kinase 2, mitochondrial | Homo sapiens | O00142; B4DGJ7; B4DZK7; B7ZAB1; E5KNQ5; E9PH08; O15238 | [87] | 6 | 2 | 16 | ALRCFGP | 0 | 304 | 1 | 0.8685 | 0 |
|  |  |  |  |  |  |  | 54 | SVICVEG | 0 | 310 | 1 | 1.1428 | 1 |
|  |  |  |  |  |  |  | 66 | KTTCLEF | 0 | 310 | 1 | 1.0520 | 1 |
|  |  |  |  |  |  |  | **189** | **PETCYQR** | 1 | 287 | 0 | 1.0210 | 1 |
|  |  |  |  |  |  |  | 187 | KKRCREE | 0 | 307 | 1 | 0.9068 | 0 |
|  |  |  |  |  |  |  | **264** | **RKHCP--** | 1 | 283 | 0 | 1.0600 | 1 |
| **124** | Thioredoxin reductase | Pseudoalteromonas haloplanktis | Q3IH37 | [88] | 5 | 1 | 7 | AKHCKLL | 0 | 323 | 1 | 0.8898 | 0 |
|  |  |  |  |  |  |  | 106 | TYTCDAL | 0 | 305 | 1 | 1.0178 | 1 |
|  |  |  |  |  |  |  | 136 | VSACATC | 0 | 307 | 1 | 1.1190 | 1 |
|  |  |  |  |  |  |  | 139 | CATCDGF | 0 | 280 | 0 | 1.1245 | 1 |
|  |  |  |  |  |  |  | **303** | **GTGCMAA** | 1 | 327 | 1 | 1.0873 | 1 |
| **125** | Sodium/potassium-transporting ATPase subunit beta-1 | Sus scrofa | P05027 | [89] | 6 | 1 | **45** | **FYGCLAG** | 1 | 311 | 1 | 1.0073 | 1 |
|  |  |  |  |  |  |  | 126 | FEDCGNV | 0 | 297 | 1 | 1.1343 | 1 |
|  |  |  |  |  |  |  | 149 | RKVCRFR | 0 | 292 | 0 | 0.9668 | 0 |
|  |  |  |  |  |  |  | 175 | GKPCVII | 0 | 287 | 0 | 1.1388 | 1 |
|  |  |  |  |  |  |  | 213 | PVHCTGK | 0 | 297 | 1 | 0.9725 | 0 |
|  |  |  |  |  |  |  | 276 | RIECKAY | 0 | 292 | 0 | 1.0700 | 1 |
| **126** | 1-cys-glutaredoxin-like protein-1 | Plasmodium falciparum | Q7K5X2 | [90] | 1 | 1 | **99** | **KPLCGFS** | 1 | 328 | 1 | 1.0595 | 1 |
| **127** | ATP synthase subunit alpha, mitochondrial | Homo sapiens | P25705; A8K092; B4DY56; K7ENP3; Q53XX6; Q8IXV2; Q96FB4; Q96HW2; Q96IR6; Q9BTV8 | [91] | 2 | 1 | 244 | KLYCIYV | 0 | 279 | 0 | 0.9683 | 0 |
|  |  |  |  |  |  |  | **294** | **YSGCSMG** | 1 | 301 | 1 | 0.9585 | 0 |
| **128** | Ascorbate peroxidase | Galdieria partita | Q8GT26 | [92] | 3 | 1 | 38 | LYICYIC | 0 | 275 | 0 | 0.9990 | 0 |
|  |  |  |  |  |  |  | **41** | **CYICPFA** | 1 | 288 | 0 | 1.1078 | 1 |
|  |  |  |  |  |  |  | 53 | TRNCKGL | 0 | 296 | 1 | 0.9530 | 0 |
| **129** | Putative In2.1 protein | Triticum aestivum | O82071 | [93] | 3 | 2 | **25** | **QTPCMPI** | 1 | 292 | 0 | 1.0968 | 1 |
|  |  |  |  |  |  |  | **121** | **PDACPEE** | 1 | 320 | 1 | 1.3275 | 1 |
|  |  |  |  |  |  |  | 163 | LGRCHKE | 0 | 300 | 1 | 0.7883 | 0 |
| **130** | B9GRA5 | Populus trichocarpa | B9GRA5 | [94] | 4 | 1 | **29** | **LPICSTP** | 1 | 317 | 1 | 1.0468 | 1 |
|  |  |  |  |  |  |  | 47 | TSTCSRI | 0 | 290 | 0 | 0.9628 | 0 |
|  |  |  |  |  |  |  | 101 | KTWCSYS | 0 | 304 | 1 | 0.8490 | 0 |
|  |  |  |  |  |  |  | 165 | LAWCLSL | 0 | 304 | 1 | 0.9158 | 0 |
| **131** | HIV-1 protease | Human immunodeficiency virus | Q9YQ30 | [27] [95] | 2 | 2 | **67** | **IEICGKK** | 1 | 318 | 1 | 1.0968 | 1 |
|  |  |  |  |  |  |  | **95** | **QIGCTLN** | 1 | 308 | 1 | 1.1095 | 1 |
| **132** | Fatty acid-binding protein, liver | Bos taurus | P80425; Q2M2U0 | [96] | 1 | 1 | **69** | **GEECEME** | 1 | 299 | 1 | 1.1115 | 1 |
| **133** | Alpha-crystallin A chain | Homo sapiens | P02489; Q53X53 | [97] [98] | 2 | 2 | **131** | **ALSCSLS** | 1 | 330 | 1 | 0.9065 | 0 |
|  |  |  |  |  |  |  | **142** | **LTFCGPK** | 1 | 325 | 1 | 1.0955 | 1 |
| **134** | Glutathione S-transferase GST-6.0 | Proteus mirabilis | P15214 | [99] | 3 | 1 | **10** | **PGSCSLS** | 1 | 320 | 1 | 0.9323 | 0 |
|  |  |  |  |  |  |  | 145 | KQKCVCG | 0 | 285 | 0 | 0.7880 | 0 |
|  |  |  |  |  |  |  | 147 | KCVCGDH | 0 | 293 | 0 | 0.8630 | 0 |
| **135** | Beta-crystallin A3 | Homo sapiens | P05813; Q13633; Q14CM9 | [100] | 5 | 2 | 52 | TSSCPNV | 0 | 285 | 0 | 1.0580 | 1 |
|  |  |  |  |  |  |  | **82** | **TSFCGQQ** | 1 | 292 | 0 | 1.0030 | 1 |
|  |  |  |  |  |  |  | **117** | **RPICSAN** | 1 | 318 | 1 | 1.0258 | 1 |
|  |  |  |  |  |  |  | 170 | AWVCYQY | 0 | 280 | 0 | 0.8765 | 0 |
|  |  |  |  |  |  |  | 185 | ILECDHH | 0 | 286 | 0 | 1.0368 | 1 |
| **136** | Superoxide dismutase [Cu-Zn] | Gallus gallus | P80566; Q92059 | [101]  [101] | 5 | 1 | 8 | KAVCVMK | 0 | 298 | 1 | 1.0770 | 1 |
|  |  |  |  |  |  |  | 58 | TNGCTSA | 0 | 304 | 1 | 1.0998 | 1 |
|  |  |  |  |  |  |  | 111 | GPHCIIG | 0 | 289 | 0 | 0.9850 | 0 |
|  |  |  |  |  |  |  | 146 | RLACGVI | 0 | 321 | 1 | 1.1338 | 1 |
|  |  |  |  |  |  |  | **154** | **IAKC---** | 1 | 271 | 0 | 1.0510 | 1 |
| **136** | Selenoprotein W | Macaca mulatta | P63303; O15532; O19096; Q86TI9; Q96KM5 | [102] | 2 | 1 | 10 | VVYCGAU | 0 | 306 | 1 | 1.0880 | 1 |
|  |  |  |  |  |  |  | **37** | **LDICGEG** | 1 | 315 | 1 | 1.2328 | 1 |
| **138** | Acylphosphatase-2 | Equus caballus | P00818 | [103] | 1 | 1 | **22** | **QGVCFRM** | 1 | 291 | 0 | 1.0075 | 1 |
| **139** | Cellular tumor antigen p53 | Homo sapiens | P04637; Q15086; Q15087; Q15088; Q16535; Q16807; Q16808; Q16809; Q16810; Q16811; Q16848; Q2XN98; Q3LRW1; Q3LRW2; Q3LRW3; Q3LRW4; Q3LRW5; Q86UG1; Q8J016; Q99659; Q9BTM4; Q9HAQ8; Q9NP68; Q9NPJ2; Q9NZD0; Q9UBI2; Q9UQ61 | [1]  [104] [105] [106] [107] [108] | 10 | 3 | **124** | **SVTCTYS** | 1 | 298 | 1 | 1.0443 | 1 |
|  |  |  |  |  |  |  | 135 | KMFCQLA | 0 | 289 | 0 | 0.8400 | 0 |
|  |  |  |  |  |  |  | **141** | **AKTCPVQ** | 1 | 306 | 1 | 1.1863 | 1 |
|  |  |  |  |  |  |  | 176 | VRRCPHH | 0 | 281 | 0 | 0.9020 | 0 |
|  |  |  |  |  |  |  | **182** | **HERCSDS** | 1 | 299 | 1 | 0.8530 | 0 |
|  |  |  |  |  |  |  | 229 | GSDCTTI | 0 | 297 | 1 | 1.1670 | 1 |
|  |  |  |  |  |  |  | 238 | NYMCNSS | 0 | 278 | 0 | 0.9478 | 0 |
|  |  |  |  |  |  |  | 242 | NSSCMGG | 0 | 289 | 0 | 0.9820 | 0 |
|  |  |  |  |  |  |  | 275 | VRVCACP | 0 | 286 | 0 | 0.7458 | 0 |
|  |  |  |  |  |  |  | 277 | VCACPGR | 0 | 308 | 1 | 1.0118 | 1 |
| 140 | Glyceraldehyde-3-phosphate dehydrogenase  GAPDH | Homo sapiens (Human) | P04406 | [1] | 3 | 2 | **152** | **NASCTTN** | 1 | 299 | 1 | 1.1550 | 1 |
|  |  |  |  |  |  |  | **156** | **TTNCLAP** | 1 | 313 | 1 | 1.0470 | 1 |
|  |  |  |  |  |  |  | 247 | DLTCRLE | 0 | 311 | 1 | 0.9173 | 0 |

**References**

[1] J.J. Mieyal, M.M. Gallogly, S. Qanungo, E.A. Sabens, M.D. Shelton, Molecular Mechanisms and Clinical Implications of Reversible Protein S-Glutathionylation, Antioxid. Redox Signal. 10 (2008) 1941–1988. https://doi.org/10.1089/ars.2008.2089.

[2] Y. Hamnell-Pamment, C. Lind, C. Palmberg, T. Bergman, I.A. Cotgreave, Determination of site-specificity of S-glutathionylated cellular proteins, Biochem. Biophys. Res. Commun. 332 (2005) 362–369. https://doi.org/10.1016/j.bbrc.2005.04.130.

[3] H. Murata, Y. Ihara, H. Nakamura, J. Yodoi, K. Sumikawa, T. Kondo, Glutaredoxin exerts an antiapoptotic effect by regulating the redox state of Akt, J. Biol. Chem. 278 (2003) 50226–50233. https://doi.org/10.1074/jbc.M310171200.

[4] L. Regazzoni, A. Panusa, K.-J. Yeum, M. Carini, G. Aldini, Hemoglobin glutathionylation can occur through cysteine sulfenic acid intermediate: electrospray ionization LTQ-Orbitrap hybrid mass spectrometry studies, J. Chromatogr. B Analyt. Technol. Biomed. Life. Sci. 877 (2009) 3456–3461. https://doi.org/10.1016/j.jchromb.2009.05.020.

[5] O. Gorelenkova Miller, J.J. Mieyal, Sulfhydryl-mediated redox signaling in inflammation: role in neurodegenerative diseases, Arch. Toxicol. 89 (2015) 1439–1467. https://doi.org/10.1007/s00204-015-1496-7.

[6] F. Meissner, K. Molawi, A. Zychlinsky, Superoxide dismutase 1 regulates caspase-1 and endotoxic shock, Nat. Immunol. 9 (2008) 866–872. https://doi.org/10.1038/ni.1633.

[7] B.G. Hill, A. Bhatnagar, Protein S-glutathiolation: redox-sensitive regulation of protein function, J. Mol. Cell. Cardiol. 52 (2012) 559–567. https://doi.org/10.1016/j.yjmcc.2011.07.009.

[8] E.M.G. Allen, J.J. Mieyal, Protein-thiol oxidation and cell death: regulatory role of glutaredoxins, Antioxid. Redox Signal. 17 (2012) 1748–1763. https://doi.org/10.1089/ars.2012.4644.

[9] V. Anathy, S.W. Aesif, A.S. Guala, M. Havermans, N.L. Reynaert, Y.-S. Ho, R.C. Budd, Y.M.W. Janssen-Heininger, Redox amplification of apoptosis by caspase-dependent cleavage of glutaredoxin 1 and S-glutathionylation of Fas, J. Cell Biol. 184 (2009) 241–252. https://doi.org/10.1083/jcb.200807019.

[10] G. Manao, G. Camici, G. Cappugi, M. Stefani, G. Liguri, A. Berti, G. Ramponi, Rabbit skeletal muscle acylphosphatase: the amino acid sequence of form Ra1, Arch. Biochem. Biophys. 241 (1985) 418–424.

[11] S.J. Harrop, M.Z. DeMaere, W.D. Fairlie, T. Reztsova, S.M. Valenzuela, M. Mazzanti, R. Tonini, M.R. Qiu, L. Jankova, K. Warton, A.R. Bauskin, W.M. Wu, S. Pankhurst, T.J. Campbell, S.N. Breit, P.M. Curmi, Crystal structure of a soluble form of the intracellular chloride ion channel CLIC1 (NCC27) at 1.4-A resolution, J. Biol. Chem. 276 (2001) 44993–45000. https://doi.org/10.1074/jbc.M107804200.

[12] S.O. Kim, K. Merchant, R. Nudelman, W.F. Beyer, T. Keng, J. DeAngelo, A. Hausladen, J.S. Stamler, OxyR: a molecular code for redox-related signaling, Cell. 109 (2002) 383–396.

[13] E. Cabiscol, R.L. Levine, The phosphatase activity of carbonic anhydrase III is reversibly regulated by glutathiolation, Proc. Natl. Acad. Sci. U. S. A. 93 (1996) 4170–4174.

[14] J.F. Caplan, N.R. Filipenko, S.L. Fitzpatrick, D.M. Waisman, Regulation of annexin A2 by reversible glutathionylation, J. Biol. Chem. 279 (2004) 7740–7750. https://doi.org/10.1074/jbc.M313049200.

[15] C.-A. Chen, T.-Y. Wang, S. Varadharaj, L.A. Reyes, C. Hemann, M.A.H. Talukder, Y.-R. Chen, L.J. Druhan, J.L. Zweier, S-glutathionylation uncouples eNOS and regulates its cellular and vascular function, Nature. 468 (2010) 1115–1118. https://doi.org/10.1038/nature09599.

[16] P.C. Chong, R.S. Hodges, Proximity of sulfhydryl groups to the sites of interaction between components of the troponin complex from rabbit skeletal muscle, J. Biol. Chem. 257 (1982) 2549–2555.

[17] Y.-R. Chen, C.-L. Chen, D.R. Pfeiffer, J.L. Zweier, Mitochondrial complex II in the post-ischemic heart: oxidative injury and the role of protein S-glutathionylation, J. Biol. Chem. 282 (2007) 32640–32654. https://doi.org/10.1074/jbc.M702294200.

[18] R.S. Zee, C.B. Yoo, D.R. Pimentel, D.H. Perlman, J.R. Burgoyne, X. Hou, M.E. McComb, C.E. Costello, R.A. Cohen, M.M. Bachschmid, Redox regulation of sirtuin-1 by S-glutathiolation, Antioxid. Redox Signal. 13 (2010) 1023–1032. https://doi.org/10.1089/ars.2010.3251.

[19] A.B. Makar, K.E. McMartin, M. Palese, T.R. Tephly, Formate assay in body fluids: application in methanol poisoning, Biochem. Med. 13 (1975) 117–126.

[20] S. Casagrande, V. Bonetto, M. Fratelli, E. Gianazza, I. Eberini, T. Massignan, M. Salmona, G. Chang, A. Holmgren, P. Ghezzi, Glutathionylation of human thioredoxin: a possible crosstalk between the glutathione and thioredoxin systems, Proc. Natl. Acad. Sci. U. S. A. 99 (2002) 9745–9749. https://doi.org/10.1073/pnas.152168599.

[21] J. Li, F.L. Huang, K.P. Huang, Glutathiolation of proteins by glutathione disulfide S-oxide derived from S-nitrosoglutathione. Modifications of rat brain neurogranin/RC3 and neuromodulin/GAP-43, J. Biol. Chem. 276 (2001) 3098–3105. https://doi.org/10.1074/jbc.M008260200.

[22] E. Truppo, C.T. Supuran, A. Sandomenico, D. Vullo, A. Innocenti, A. Di Fiore, V. Alterio, G. De Simone, S.M. Monti, Carbonic anhydrase VII is S-glutathionylated without loss of catalytic activity and affinity for sulfonamide inhibitors, Bioorg. Med. Chem. Lett. 22 (2012) 1560–1564. https://doi.org/10.1016/j.bmcl.2011.12.134.

[23] W.C. Barrett, J.P. DeGnore, S. König, H.M. Fales, Y.F. Keng, Z.Y. Zhang, M.B. Yim, P.B. Chock, Regulation of PTP1B via glutathionylation of the active site cysteine 215, Biochemistry. 38 (1999) 6699–6705. https://doi.org/10.1021/bi990240v.

[24] I.Y. Petrushanko, S. Yakushev, V.A. Mitkevich, Y.V. Kamanina, R.H. Ziganshin, X. Meng, A.A. Anashkina, A. Makhro, O.D. Lopina, M. Gassmann, S-Glutathionylation of the Na, k-atpase catalytic α subunit is a determinant of the enzyme redox sensitivity, J. Biol. Chem. 287 (2012) 32195–32205.

[25] E.A. Dergousova, I.Y. Petrushanko, E.A. Klimanova, V.A. Mitkevich, R.H. Ziganshin, O.D. Lopina, A.A. Makarov, Effect of Reduction of Redox Modifications of Cys-Residues in the Na,K-ATPase α1-Subunit on Its Activity, Biomolecules. 7 (2017). https://doi.org/10.3390/biom7010018.

[26] K.M. Humphries, C. Juliano, S.S. Taylor, Regulation of cAMP-dependent protein kinase activity by glutathionylation, J. Biol. Chem. 277 (2002) 43505–43511. https://doi.org/10.1074/jbc.M207088200.

[27] D.A. Davis, F.M. Newcomb, D.W. Starke, D.E. Ott, J.J. Mieyal, R. Yarchoan, Thioltransferase (glutaredoxin) is detected within HIV-1 and can regulate the activity of glutathionylated HIV-1 protease in vitro, J. Biol. Chem. 272 (1997) 25935–25940.

[28] K. Yang, M. Wang, Y. Zhao, X. Sun, Y. Yang, X. Li, A. Zhou, H. Chu, H. Zhou, J. Xu, M. Wu, J. Yang, J. Yi, A redox mechanism underlying nucleolar stress sensing by nucleophosmin, Nat. Commun. 7 (2016) 13599. https://doi.org/10.1038/ncomms13599.

[29] J.C. Klein, R.J. Moen, E.A. Smith, M.A. Titus, D.D. Thomas, Structural and Functional Impact of Site-Directed Methionine Oxidation in Myosin, Biochemistry. 50 (2011) 10318–10327. https://doi.org/10.1021/bi201279u.

[30] B. McDonagh, C.A. Padilla, J.R. Pedrajas, J.A. Bárcena, Biosynthetic and iron metabolism is regulated by thiol proteome changes dependent on glutaredoxin-2 and mitochondrial peroxiredoxin-1 in Saccharomyces cerevisiae, J. Biol. Chem. 286 (2011) 15565–15576. https://doi.org/10.1074/jbc.M110.193102.

[31] B. McDonagh, R. Requejo, C.A. Fuentes-Almagro, S. Ogueta, J.A. Bárcena, C.A. Padilla, Thiol redox proteomics identifies differential targets of cytosolic and mitochondrial glutaredoxin-2 isoforms in Saccharomyces cerevisiae. Reversible S-glutathionylation of DHBP synthase (RIB3), J. Proteomics. 74 (2011) 2487–2497. https://doi.org/10.1016/j.jprot.2011.04.018.

[32] N. Brandes, D. Reichmann, H. Tienson, L.I. Leichert, U. Jakob, Using quantitative redox proteomics to dissect the yeast redoxome, J. Biol. Chem. 286 (2011) 41893–41903. https://doi.org/10.1074/jbc.M111.296236.

[33] Y. Yang, W. Shi, N. Cui, Z. Wu, C. Jiang, Oxidative stress inhibits vascular K(ATP) channels by S-glutathionylation, J. Biol. Chem. 285 (2010) 38641–38648. https://doi.org/10.1074/jbc.M110.162578.

[34] Y. Yang, W. Shi, X. Chen, N. Cui, A.S. Konduru, Y. Shi, T.C. Trower, S. Zhang, C. Jiang, Molecular basis and structural insight of vascular K(ATP) channel gating by S-glutathionylation, J. Biol. Chem. 286 (2011) 9298–9307. https://doi.org/10.1074/jbc.M110.195123.

[35] W.-W. Shi, Y. Yang, Y. Shi, C. Jiang, K(ATP) channel action in vascular tone regulation: from genetics to diseases, Sheng Li Xue Bao. 64 (2012) 1–13.

[36] L. Wobbe, O. Blifernez, C. Schwarz, J.H. Mussgnug, J. Nickelsen, O. Kruse, Cysteine modification of a specific repressor protein controls the translational status of nucleus-encoded LHCII mRNAs in Chlamydomonas, Proc. Natl. Acad. Sci. U. S. A. 106 (2009) 13290–13295. https://doi.org/10.1073/pnas.0900670106.

[37] C.-L. Chen, L. Zhang, A. Yeh, C.-A. Chen, K.B. Green-Church, J.L. Zweier, Y.-R. Chen, Site-specific S-glutathiolation of mitochondrial NADH ubiquinone reductase, Biochemistry. 46 (2007) 5754–5765. https://doi.org/10.1021/bi602580c.

[38] S. Maiti, J. Zhang, G. Chen, Redox regulation of human estrogen sulfotransferase (hSULT1E1), Biochem. Pharmacol. 73 (2007) 1474–1481. https://doi.org/10.1016/j.bcp.2006.12.026.

[39] D.R. Pimentel, T. Adachi, Y. Ido, T. Heibeck, B. Jiang, Y. Lee, J.A. Melendez, R.A. Cohen, W.S. Colucci, Strain-stimulated hypertrophy in cardiac myocytes is mediated by reactive oxygen species-dependent Ras S-glutathiolation, J. Mol. Cell. Cardiol. 41 (2006) 613–622. https://doi.org/10.1016/j.yjmcc.2006.05.009.

[40] X. Cao, F. Kambe, S. Ohmori, H. Seo, Oxidoreductive modification of two cysteine residues in paired domain by Ref-1 regulates DNA-binding activity of Pax-8, Biochem. Biophys. Res. Commun. 297 (2002) 288–293.

[41] X. Cao, F. Kambe, X. Lu, N. Kobayashi, S. Ohmori, H. Seo, Glutathionylation of two cysteine residues in paired domain regulates DNA binding activity of Pax-8, J. Biol. Chem. 280 (2005) 25901–25906. https://doi.org/10.1074/jbc.M411443200.

[42] L. Codutti, H. van Ingen, C. Vascotto, F. Fogolari, A. Corazza, G. Tell, F. Quadrifoglio, P. Viglino, R. Boelens, G. Esposito, The solution structure of DNA-free Pax-8 paired box domain accounts for redox regulation of transcriptional activity in the pax protein family, J. Biol. Chem. 283 (2008) 33321–33328. https://doi.org/10.1074/jbc.M805717200.

[43] D.A. Davis, K. Dorsey, P.T. Wingfield, S.J. Stahl, J. Kaufman, H.M. Fales, R.L. Levine, Regulation of HIV-1 protease activity through cysteine modification, Biochemistry. 35 (1996) 2482–2488. https://doi.org/10.1021/bi951525k.

[44] D.A. Davis, C.A. Brown, F.M. Newcomb, E.S. Boja, H.M. Fales, J. Kaufman, S.J. Stahl, P. Wingfield, R. Yarchoan, Reversible oxidative modification as a mechanism for regulating retroviral protease dimerization and activation, J. Virol. 77 (2003) 3319–3325.

[45] H. Cheng, T. Tchaikovskaya, Y.S. Tu, J. Chapman, B. Qian, W.M. Ching, M. Tien, J.D. Rowe, Y.V. Patskovsky, I. Listowsky, C.P. Tu, Rat glutathione S-transferase M4-4: an isoenzyme with unique structural features including a redox-reactive cysteine-115 residue that forms mixed disulphides with glutathione, Biochem. J. 356 (2001) 403–414.

[46] M.E. Martin, Y. Chinenov, M. Yu, T.K. Schmidt, X.Y. Yang, Redox regulation of GA-binding protein-alpha DNA binding activity, J. Biol. Chem. 271 (1996) 25617–25623.

[47] Y. Chinenov, T. Schmidt, X.Y. Yang, M.E. Martin, Identification of redox-sensitive cysteines in GA-binding protein-alpha that regulate DNA binding and heterodimerization, J. Biol. Chem. 273 (1998) 6203–6209.

[48] G. Kim, J. Selengut, R.L. Levine, Carbonic anhydrase III: the phosphatase activity is extrinsic, Arch. Biochem. Biophys. 377 (2000) 334–340. https://doi.org/10.1006/abbi.2000.1793.

[49] R.J. Mallis, B.W. Poland, T.K. Chatterjee, R.A. Fisher, S. Darmawan, R.B. Honzatko, J.A. Thomas, Crystal structure of S-glutathiolated carbonic anhydrase III, FEBS Lett. 482 (2000) 237–241.

[50] G. Kim, R.L. Levine, Molecular determinants of S-glutathionylation of carbonic anhydrase 3, Antioxid. Redox Signal. 7 (2005) 849–854. https://doi.org/10.1089/ars.2005.7.849.

[51] K. Konishi, M. Fujioka, Reversible inactivation of recombinant rat liver guanidinoacetate methyltransferase by glutathione disulfide, Arch. Biochem. Biophys. 289 (1991) 90–96.

[52] I.S. Kil, S.Y. Kim, J.-W. Park, Glutathionylation regulates IkappaB, Biochem. Biophys. Res. Commun. 373 (2008) 169–173. https://doi.org/10.1016/j.bbrc.2008.06.007.

[53] J.V. Cross, D.J. Templeton, Oxidative stress inhibits MEKK1 by site-specific glutathionylation in the ATP-binding domain, Biochem. J. 381 (2004) 675–683. https://doi.org/10.1042/BJ20040591.

[54] H.M.S. Shahul, S.P. Sarma, The structure of the thioredoxin-triosephosphate isomerase complex provides insights into the reversible glutathione-mediated regulation of triosephosphate isomerase, Biochemistry. 51 (2012) 533–544. https://doi.org/10.1021/bi201224s.

[55] I.S. Kil, J.-W. Park, Regulation of mitochondrial NADP+-dependent isocitrate dehydrogenase activity by glutathionylation, J. Biol. Chem. 280 (2005) 10846–10854. https://doi.org/10.1074/jbc.M411306200.

[56] C. Fu, J. Hu, T. Liu, T. Ago, J. Sadoshima, H. Li, Quantitative analysis of redox-sensitive proteome with DIGE and ICAT, J. Proteome Res. 7 (2008) 3789–3802. https://doi.org/10.1021/pr800233r.

[57] C. Fu, C. Wu, T. Liu, T. Ago, P. Zhai, J. Sadoshima, H. Li, Elucidation of thioredoxin target protein networks in mouse, Mol. Cell. Proteomics MCP. 8 (2009) 1674–1687. https://doi.org/10.1074/mcp.M800580-MCP200.

[58] C.H. Lillig, A. Potamitou, J.-D. Schwenn, A. Vlamis-Gardikas, A. Holmgren, Redox regulation of 3’-phosphoadenylylsulfate reductase from Escherichia coli by glutathione and glutaredoxins, J. Biol. Chem. 278 (2003) 22325–22330. https://doi.org/10.1074/jbc.M302304200.

[59] W. Wang, C. Oliva, G. Li, A. Holmgren, C.H. Lillig, K.L. Kirk, Reversible silencing of CFTR chloride channels by glutathionylation, J. Gen. Physiol. 125 (2005) 127–141. https://doi.org/10.1085/jgp.200409115.

[60] L.V. Papp, J. Lu, F. Striebel, D. Kennedy, A. Holmgren, K.K. Khanna, The redox state of SECIS binding protein 2 controls its localization and selenocysteine incorporation function, Mol. Cell. Biol. 26 (2006) 4895–4910. https://doi.org/10.1128/MCB.02284-05.

[61] E.R. Hondorp, R.G. Matthews, Oxidative stress inactivates cobalamin-independent methionine synthase (MetE) in Escherichia coli, PLoS Biol. 2 (2004) e336. https://doi.org/10.1371/journal.pbio.0020336.

[62] R. Gonzalez-Dosal, K.A. Horan, S.H. Rahbek, H. Ichijo, Z.J. Chen, J.J. Mieyal, R. Hartmann, S.R. Paludan, HSV infection induces production of ROS, which potentiate signaling from pattern recognition receptors: role for S-glutathionylation of TRAF3 and 6, PLoS Pathog. 7 (2011) e1002250. https://doi.org/10.1371/journal.ppat.1002250.

[63] R.J. Mailloux, E.L. Seifert, F. Bouillaud, C. Aguer, S. Collins, M.-E. Harper, Glutathionylation acts as a control switch for uncoupling proteins UCP2 and UCP3, J. Biol. Chem. 286 (2011) 21865–21875. https://doi.org/10.1074/jbc.M111.240242.

[64] C.A. Staab, T. Hartmanová, Y. El-Hawari, B. Ebert, M. Kisiela, V. Wsol, H.-J. Martin, E. Maser, Studies on reduction of S-nitrosoglutathione by human carbonyl reductases 1 and 3, Chem. Biol. Interact. 191 (2011) 95–103. https://doi.org/10.1016/j.cbi.2011.01.016.

[65] J.W. Zmijewski, S. Banerjee, H. Bae, A. Friggeri, E.R. Lazarowski, E. Abraham, Exposure to hydrogen peroxide induces oxidation and activation of AMP-activated protein kinase, J. Biol. Chem. 285 (2010) 33154–33164. https://doi.org/10.1074/jbc.M110.143685.

[66] B.J. Hawkins, K.M. Irrinki, K. Mallilankaraman, Y.-C. Lien, Y. Wang, C.D. Bhanumathy, R. Subbiah, M.F. Ritchie, J. Soboloff, Y. Baba, T. Kurosaki, S.K. Joseph, D.L. Gill, M. Madesh, S-glutathionylation activates STIM1 and alters mitochondrial homeostasis, J. Cell Biol. 190 (2010) 391–405. https://doi.org/10.1083/jcb.201004152.

[67] T. Kambe, T. Song, T. Takata, N. Hatano, Y. Miyamoto, N. Nozaki, Y. Naito, H. Tokumitsu, Y. Watanabe, Inactivation of Ca2+/calmodulin-dependent protein kinase I by S-glutathionylation of the active-site cysteine residue, FEBS Lett. 584 (2010) 2478–2484. https://doi.org/10.1016/j.febslet.2010.04.059.

[68] T. Takata, J. Kimura, Y. Tsuchiya, Y. Naito, Y. Watanabe, Calcium/calmodulin-dependent protein kinases as potential targets of nitric oxide, Nitric Oxide Biol. Chem. 25 (2011) 145–152. https://doi.org/10.1016/j.niox.2011.01.004.

[69] M. Bedhomme, M. Zaffagnini, C.H. Marchand, X.-H. Gao, M. Moslonka-Lefebvre, L. Michelet, P. Decottignies, S.D. Lemaire, Regulation by glutathionylation of isocitrate lyase from Chlamydomonas reinhardtii, J. Biol. Chem. 284 (2009) 36282–36291. https://doi.org/10.1074/jbc.M109.064428.

[70] L. Michelet, M. Zaffagnini, H. Vanacker, P. Le Maréchal, C. Marchand, M. Schroda, S.D. Lemaire, P. Decottignies, In vivo targets of S-thiolation in Chlamydomonas reinhardtii, J. Biol. Chem. 283 (2008) 21571–21578. https://doi.org/10.1074/jbc.M802331200.

[71] D.M. Townsend, Y. Manevich, L. He, S. Hutchens, C.J. Pazoles, K.D. Tew, Novel role for glutathione S-transferase pi. Regulator of protein S-Glutathionylation following oxidative and nitrosative stress, J. Biol. Chem. 284 (2009) 436–445. https://doi.org/10.1074/jbc.M805586200.

[72] J.W. Baty, M.B. Hampton, C.C. Winterbourn, Proteomic detection of hydrogen peroxide-sensitive thiol proteins in Jurkat cells, Biochem. J. 389 (2005) 785–795. https://doi.org/10.1042/BJ20050337.

[73] I. Castellano, M.R. Ruocco, F. Cecere, A. Di Maro, A. Chambery, A. Michniewicz, G. Parlato, M. Masullo, E. De Vendittis, Glutathionylation of the iron superoxide dismutase from the psychrophilic eubacterium Pseudoalteromonas haloplanktis, Biochim. Biophys. Acta. 1784 (2008) 816–826. https://doi.org/10.1016/j.bbapap.2008.02.003.

[74] J. Craghill, A.D. Cronshaw, J.J. Harding, The identification of a reaction site of glutathione mixed-disulphide formation on gammaS-crystallin in human lens, Biochem. J. 379 (2004) 595–600. https://doi.org/10.1042/BJ20031367.

[75] F. Pauwels, B. Vergauwen, F. Vanrobaeys, B. Devreese, J.J. Van Beeumen, Purification and characterization of a chimeric enzyme from Haemophilus influenzae Rd that exhibits glutathione-dependent peroxidase activity, J. Biol. Chem. 278 (2003) 16658–16666. https://doi.org/10.1074/jbc.M300157200.

[76] C.R. Borges, T. Geddes, J.T. Watson, D.M. Kuhn, Dopamine biosynthesis is regulated by S-glutathionylation. Potential mechanism of tyrosine hydroxylast inhibition during oxidative stress, J. Biol. Chem. 277 (2002) 48295–48302. https://doi.org/10.1074/jbc.M209042200.

[77] J.P. Brennan, J.I.A. Miller, W. Fuller, R. Wait, S. Begum, M.J. Dunn, P. Eaton, The utility of N,N-biotinyl glutathione disulfide in the study of protein S-glutathiolation, Mol. Cell. Proteomics MCP. 5 (2006) 215–225. https://doi.org/10.1074/mcp.M500212-MCP200.

[78] B. McDonagh, S. Ogueta, G. Lasarte, C.A. Padilla, J.A. Bárcena, Shotgun redox proteomics identifies specifically modified cysteines in key metabolic enzymes under oxidative stress in Saccharomyces cerevisiae, J. Proteomics. 72 (2009) 677–689.

[79] J. Chen, C.-L. Chen, S. Rawale, C.-A. Chen, J.L. Zweier, P.T.P. Kaumaya, Y.-R. Chen, Peptide-based antibodies against glutathione-binding domains suppress superoxide production mediated by mitochondrial complex I, J. Biol. Chem. 285 (2010) 3168–3180. https://doi.org/10.1074/jbc.M109.056846.

[80] C.-L. Chen, J. Chen, S. Rawale, S. Varadharaj, P.P.T. Kaumaya, J.L. Zweier, Y.-R. Chen, Protein tyrosine nitration of the flavin subunit is associated with oxidative modification of mitochondrial complex II in the post-ischemic myocardium, J. Biol. Chem. 283 (2008) 27991–28003. https://doi.org/10.1074/jbc.M802691200.

[81] P. Aracena, G. Sánchez, P. Donoso, S.L. Hamilton, C. Hidalgo, S-glutathionylation decreases Mg2+ inhibition and S-nitrosylation enhances Ca2+ activation of RyR1 channels, J. Biol. Chem. 278 (2003) 42927–42935. https://doi.org/10.1074/jbc.M306969200.

[82] P. Aracena, W. Tang, S.L. Hamilton, C. Hidalgo, Effects of S-glutathionylation and S-nitrosylation on calmodulin binding to triads and FKBP12 binding to type 1 calcium release channels, Antioxid. Redox Signal. 7 (2005) 870–881. https://doi.org/10.1089/ars.2005.7.870.

[83] G. Sánchez, Z. Pedrozo, R.J. Domenech, C. Hidalgo, P. Donoso, Tachycardia increases NADPH oxidase activity and RyR2 S-glutathionylation in ventricular muscle, J. Mol. Cell. Cardiol. 39 (2005) 982–991. https://doi.org/10.1016/j.yjmcc.2005.08.010.

[84] C. Hidalgo, G. Sánchez, G. Barrientos, P. Aracena-Parks, A transverse tubule NADPH oxidase activity stimulates calcium release from isolated triads via ryanodine receptor type 1 S -glutathionylation, J. Biol. Chem. 281 (2006) 26473–26482. https://doi.org/10.1074/jbc.M600451200.

[85] P. Aracena-Parks, S.A. Goonasekera, C.P. Gilman, R.T. Dirksen, C. Hidalgo, S.L. Hamilton, Identification of cysteines involved in S-nitrosylation, S-glutathionylation, and oxidation to disulfides in ryanodine receptor type 1, J. Biol. Chem. 281 (2006) 40354–40368. https://doi.org/10.1074/jbc.M600876200.

[86] G. Sánchez, M. Escobar, Z. Pedrozo, P. Macho, R. Domenech, S. Härtel, C. Hidalgo, P. Donoso, Exercise and tachycardia increase NADPH oxidase and ryanodine receptor-2 activity: possible role in cardioprotection, Cardiovasc. Res. 77 (2008) 380–386. https://doi.org/10.1093/cvr/cvm011.

[87] R. Sun, S. Eriksson, L. Wang, Oxidative stress induced S-glutathionylation and proteolytic degradation of mitochondrial thymidine kinase 2, J. Biol. Chem. 287 (2012) 24304–24312. https://doi.org/10.1074/jbc.M112.381996.

[88] P. Falasca, G. Evangelista, R. Cotugno, S. Marco, M. Masullo, E. De Vendittis, G. Raimo, Properties of the endogenous components of the thioredoxin system in the psychrophilic eubacterium Pseudoalteromonas haloplanktis TAC 125, Extrem. Life Extreme Cond. 16 (2012) 539–552. https://doi.org/10.1007/s00792-012-0453-0.

[89] C.-C. Liu, A. Garcia, Y.A. Mahmmoud, E.J. Hamilton, K.K. Galougahi, N.A.S. Fry, G.A. Figtree, F. Cornelius, R.J. Clarke, H.H. Rasmussen, Susceptibility of β1 Na+-K+ pump subunit to glutathionylation and oxidative inhibition depends on conformational state of pump, J. Biol. Chem. 287 (2012) 12353–12364. https://doi.org/10.1074/jbc.M112.340893.

[90] M. Deponte, K. Becker, S. Rahlfs, Plasmodium falciparum glutaredoxin-like proteins, Biol. Chem. 386 (2005) 33–40. https://doi.org/10.1515/BC.2005.005.

[91] S.-B. Wang, D.B. Foster, J. Rucker, B. O’Rourke, D.A. Kass, J.E. Van Eyk, Redox regulation of mitochondrial ATP synthase: implications for cardiac resynchronization therapy, Circ. Res. 109 (2011) 750–757. https://doi.org/10.1161/CIRCRESAHA.111.246124.

[92] S. Kitajima, M. Kurioka, T. Yoshimoto, M. Shindo, K. Kanaori, K. Tajima, K. Oda, A cysteine residue near the propionate side chain of heme is the radical site in ascorbate peroxidase, FEBS J. 275 (2008) 470–480. https://doi.org/10.1111/j.1742-4658.2007.06214.x.

[93] D.P. Dixon, R. Edwards, Roles for stress-inducible lambda glutathione transferases in flavonoid metabolism in plants as identified by ligand fishing, J. Biol. Chem. 285 (2010) 36322–36329. https://doi.org/10.1074/jbc.M110.164806.

[94] J. Couturier, C.S. Koh, M. Zaffagnini, A.M. Winger, J.M. Gualberto, C. Corbier, P. Decottignies, J.-P. Jacquot, S.D. Lemaire, C. Didierjean, N. Rouhier, Structure-function relationship of the chloroplastic glutaredoxin S12 with an atypical WCSYS active site, J. Biol. Chem. 284 (2009) 9299–9310. https://doi.org/10.1074/jbc.M807998200.

[95] T. Persichini, M. Colasanti, G.M. Lauro, P. Ascenzi, Cysteine nitrosylation inactivates the HIV-1 protease, Biochem. Biophys. Res. Commun. 250 (1998) 575–576. https://doi.org/10.1006/bbrc.1998.9350.

[96] P. Dörmann, T. Börchers, U. Korf, P. Højrup, P. Roepstorff, F. Spener, Amino acid exchange and covalent modification by cysteine and glutathione explain isoforms of fatty acid-binding protein occurring in bovine liver, J. Biol. Chem. 268 (1993) 16286–16292.

[97] M. Cherian, J.B. Smith, X.Y. Jiang, E.C. Abraham, Influence of protein-glutathione mixed disulfide on the chaperone-like function of alpha-crystallin, J. Biol. Chem. 272 (1997) 29099–29103.

[98] S.F. Newman, R. Sultana, M. Perluigi, R. Coccia, J. Cai, W.M. Pierce, J.B. Klein, D.M. Turner, D.A. Butterfield, An increase in S-glutathionylated proteins in the Alzheimer’s disease inferior parietal lobule, a proteomics approach, J. Neurosci. Res. 85 (2007) 1506–1514. https://doi.org/10.1002/jnr.21275.

[99] A.M. Caccuri, G. Antonini, N. Allocati, C. Di Ilio, F. De Maria, F. Innocenti, M.W. Parker, M. Masulli, M. Lo Bello, P. Turella, G. Federici, G. Ricci, GSTB1-1 from Proteus mirabilis: a snapshot of an enzyme in the evolutionary pathway from a redox enzyme to a conjugating enzyme, J. Biol. Chem. 277 (2002) 18777–18784. https://doi.org/10.1074/jbc.M201137200.

[100] V.N. Lapko, R.L. Cerny, D.L. Smith, J.B. Smith, Modifications of human betaA1/betaA3-crystallins include S-methylation, glutathiolation, and truncation, Protein Sci. Publ. Protein Soc. 14 (2005) 45–54. https://doi.org/10.1110/ps.04738505.

[101] M.E. Schininà, P. Carlini, F. Polticelli, F. Zappacosta, F. Bossa, L. Calabrese, Amino acid sequence of chicken Cu, Zn-containing superoxide dismutase and identification of glutathionyl adducts at exposed cysteine residues, Eur. J. Biochem. 237 (1996) 433–439.

[102] Q.P. Gu, M.A. Beilstein, E. Barofsky, W. Ream, P.D. Whanger, Purification, characterization, and glutathione binding to selenoprotein W from monkey muscle, Arch. Biochem. Biophys. 361 (1999) 25–33. https://doi.org/10.1006/abbi.1998.0949.

[103] G. Cappugi, G. Manao, G. Camici, G. Ramponi, The complete amino acid sequence of horse muscle acylphosphatase, J. Biol. Chem. 255 (1980) 6868–6874.

[104] X.Z. Sun, C. Vinci, L. Makmura, S. Han, D. Tran, J. Nguyen, M. Hamann, S. Grazziani, S. Sheppard, M. Gutova, F. Zhou, J. Thomas, J. Momand, Formation of disulfide bond in p53 correlates with inhibition of DNA binding and tetramerization, Antioxid. Redox Signal. 5 (2003) 655–665. https://doi.org/10.1089/152308603770310338.

[105] S. Biswas, A.S. Chida, I. Rahman, Redox modifications of protein-thiols: emerging roles in cell signaling, Biochem. Pharmacol. 71 (2006) 551–564. https://doi.org/10.1016/j.bcp.2005.10.044.

[106] C.S. Velu, S.K. Niture, C.E. Doneanu, N. Pattabiraman, K.S. Srivenugopal, Human p53 is inhibited by glutathionylation of cysteines present in the proximal DNA-binding domain during oxidative stress, Biochemistry. 46 (2007) 7765–7780. https://doi.org/10.1021/bi700425y.

[107] J.M. Held, S.R. Danielson, J.B. Behring, C. Atsriku, D.J. Britton, R.L. Puckett, B. Schilling, J. Campisi, C.C. Benz, B.W. Gibson, Targeted quantitation of site-specific cysteine oxidation in endogenous proteins using a differential alkylation and multiple reaction monitoring mass spectrometry approach, Mol. Cell. Proteomics MCP. 9 (2010) 1400–1410. https://doi.org/10.1074/mcp.M900643-MCP200.

[108] M.A. Yusuf, T. Chuang, G.J. Bhat, K.S. Srivenugopal, Cys-141 glutathionylation of human p53: Studies using specific polyclonal antibodies in cancer samples and cell lines, Free Radic. Biol. Med. 49 (2010) 908–917. https://doi.org/10.1016/j.freeradbiomed.2010.06.020.
